# Protein-Protein Interaction Site Predictions with Three-Dimensional Probability Distributions of Interacting Atoms on Protein Surfaces

**DOI:** 10.1371/journal.pone.0037706

**Published:** 2012-06-06

**Authors:** Ching-Tai Chen, Hung-Pin Peng, Jhih-Wei Jian, Keng-Chang Tsai, Jeng-Yih Chang, Ei-Wen Yang, Jun-Bo Chen, Shinn-Ying Ho, Wen-Lian Hsu, An-Suei Yang

**Affiliations:** 1 Genomics Research Center, Academia Sinica, Taipei, Taiwan; 2 Institute of Biomedical Informatics, National Yang-Ming University, Taipei, Taiwan; 3 Department of Computer Science, National Tsing-Hua University, Hsinchu, Taiwan; 4 Institute of Bioinformatics and Systems Biology, National Chiao-Tung University, Hsinchu, Taiwan; 5 Institute of Information Science, Academia Sinica, Taipei, Taiwan; 6 Bioinformatics Program, Taiwan International Graduate Program, Institute of Information Science, Academia Sinica, Taipei, Taiwan; Koc University, Turkey

## Abstract

Protein-protein interactions are key to many biological processes. Computational methodologies devised to predict protein-protein interaction (PPI) sites on protein surfaces are important tools in providing insights into the biological functions of proteins and in developing therapeutics targeting the protein-protein interaction sites. One of the general features of PPI sites is that the core regions from the two interacting protein surfaces are complementary to each other, similar to the interior of proteins in packing density and in the physicochemical nature of the amino acid composition. In this work, we simulated the physicochemical complementarities by constructing three-dimensional probability density maps of non-covalent interacting atoms on the protein surfaces. The interacting probabilities were derived from the interior of known structures. Machine learning algorithms were applied to learn the characteristic patterns of the probability density maps specific to the PPI sites. The trained predictors for PPI sites were cross-validated with the training cases (consisting of 432 proteins) and were tested on an independent dataset (consisting of 142 proteins). The residue-based Matthews correlation coefficient for the independent test set was 0.423; the accuracy, precision, sensitivity, specificity were 0.753, 0.519, 0.677, and 0.779 respectively. The benchmark results indicate that the optimized machine learning models are among the best predictors in identifying PPI sites on protein surfaces. In particular, the PPI site prediction accuracy increases with increasing size of the PPI site and with increasing hydrophobicity in amino acid composition of the PPI interface; the core interface regions are more likely to be recognized with high prediction confidence. The results indicate that the physicochemical complementarity patterns on protein surfaces are important determinants in PPIs, and a substantial portion of the PPI sites can be predicted correctly with the physicochemical complementarity features based on the non-covalent interaction data derived from protein interiors.

## Introduction

Proteins perform essential functions in biological systems through recognizing their protein partners and by forming permanent or transient protein complexes. Computational predictions of the protein-protein interaction (PPI) sites on protein surfaces can provide insights into the biological functions of the proteins at the proteomics level and into the sequence-function relationships critical in identifying key targets for therapeutics development. Works on PPI site prediction and analysis have been summarized in many recent reviews [1], [2], [3], [4], [5], [6], [7].

Protein-protein interactions have been perceived as a process driven in large part by hydrophobic interactions in the core interfaces and by polar interactions in the interface rims. The core interface regions are tightly packed as in protein interior with key residues that are mostly hydrophobic in nature (except for Arg, which is also frequently observed in PPI sites) [8], [9], [10], [11]. Energetically, only a few buried hot-spot residues in the PPI sites are responsible for the protein binding free energy (see review [12] and references therein). The rim regions surrounding the PPI core interfaces are integral parts of the PPI sites [8], [13], but the interface packing in these regions are loose with water molecules frequently observed bridging the interfaces [14]. The hydrophilic nature of the rim regions is largely indistinguishable from the hydrophilic property of the overall protein surfaces [10]. Although the trends in physicochemical and geometrical complementarity in the PPI interfaces have been demonstrated in many analyses [10], identifying clear determinants that correlate with the surface regions mediating PPIs remains challenging [3], [4]. This is particularly true for the protein surfaces mediating non-obligated protein-protein interactions [15].

Computational algorithms have been developed for PPI site predictions. A large portion of these methods are based on information embedded in amino acid sequences and on evolutionary information derived from multiple sequence alignments of homologues in the sequence databases [16], [17], [18], [19], [20], [21], [22]. In addition, prediction algorithms combining sequence and structure information have also shown successes in identifying PPI sites [3], [23], [24], [25], [26], [27]. Structural features are taken into account for better predictive capability as structure conservation is one of the important factors among interfaces [28]. Moreover, Murakami and Jones characterized surface patches with six physicochemical properties and then linearly combined the six values for a final score as PPI interface [29]. Negi and Braun used a clustering method on surface residues based on amino acid interface propensity scale for interface prediction [30]. Kufareva et al. devised 12 physical descriptors for surface patches along with a partial least square regression to predict PPI interfaces [31]. Overall, combining various sequence and structural features in training machine learning models has been succeeded to an extent in predicting PPI sites, but the PPI site predictions remain challenging with considerable difficulties [3].

The three-dimensional arrangement of amino acid residues in the PPI sites determines the affinity and specificity of the protein interactions, and hence the complementarities of surface geometry and physicochemical nature of the PPI interfaces are expected to be critical determinants in PPIs. Following this rationale, Sacquin-Mora et al. employed a rigid-body, coarse-grain docking method to detect interfaces within a small dataset [32]. A large scale PPI site prediction with docking algorithms has also been carried out recently by Wass et al., [33]. While the three-dimensional protein-protein complex model structures are likely to be predicted incorrectly, it has been found that the location of the PPI sites can be reasonably predicted with the docking algorithms [1]. The downsides of the docking algorithms are that exploring the large conformation space consumes huge computational resources and that binding geometry evaluations based on various ranking systems are not clearly effective in distinguishing the actual structures from a large set of possibilities. Template-based prediction approaches reduce the solution space of the docking approaches [2] on the premise that PPI sites are relatively conserved throughout proteins with similar sequence and structural features [28]. With the template-based approaches, high-throughput modeling of PPI sites based on protein docking have been shown with accuracy feasible for low to medium resolution models [34].

The successes of the current prediction methods, albeit limited in accuracy, have indicated that not only sequence and structural features of the query proteins are critical determinants for PPI sites, the physicochemical complementarities of the partner surfaces are also important factors in predicting the interface locations. But for most of the proteins, the complementarity information is unavailable without knowing the binding partners and the binding interfaces, which are the targets of the PPI site predictions in the first place. In this work, we circumvent the difficulty by simulating the binding surface physicochemical complementarity with three-dimensional probability density maps (PDMs), which were derived based on the distributions of non-covalent interacting atoms in protein interiors. The PDMs provide information of possible interacting atoms from the protein partners in the PPI interfaces, because the PPI interface cores share similar amino acid composition with protein interiors [10]. The PDMs were encoded into numerical features to train machine learning algorithms coupled with bootstrap aggregation (bagging) techniques [35]. One machine learning model was trained for each of the 30 protein atom types. The trained models were then used to predict PPI sites by integrating the prediction results for all the protein surface atoms on the query proteins. Five-fold cross validation was carried out with the training set composed of 432 non-redundant proteins. The cross validation yielded overall residue-based MCC (Matthews correlation coefficient) of 0.424. An independent group of 142 proteins was used as the test set. The residue-based MCC for the independent test set was 0.423, and the residue-based accuracy, precision, sensitivity, specificity were 0.753, 0.519, 0.677, and 0.779 respectively. The results are among the best predictions for PPI sites, indicating that the physicochemical complementarity derived from PDMs for protein interaction interfaces is a critical determinant for protein-protein interactions.

## Results and Discussion

### Statistical Analysis of Physicochemical Complementarities in Known PPI Interfaces

It has been well-established that geometrical and physicochemical complementarities are critical determinants in PPI interfaces [11]. The amino acid preferences and packing density for PPI core interfaces resemble those of protein interior [7], [10]. The physicochemical complementarities among interface residues are characterized by hydrophobic interactions in the core interface regions and polar interactions in the rim regions of the interfaces [8], [9], [10], [13], [36], [37]. Based on the general description of typical PPI interfaces, we hypothesized that the distribution patterns of the non-covalent interacting atoms on a PPI surface should provide abundant information in distinguishing PPI surface regions from non-PPI surface regions.

**Figure 1:**
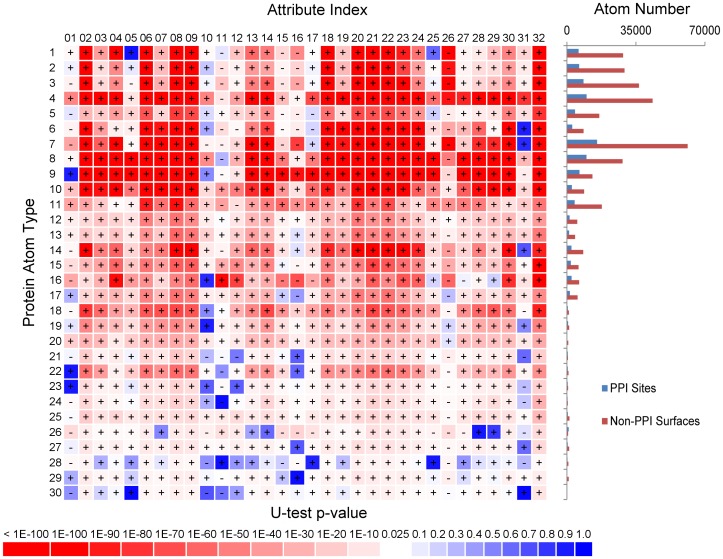
Mann-Whitney U-tests for the distributions of numerical attributes around protein surface atoms. The y-axis of matrix shows the atom type index (*i* = 30 protein atom types shown in Table 1) and the x-axis shows the *j* index for the 32 *A_i,j_* features, where *j* = 1,31 represents the 31 interacting atom types shown in Table 1 and the 32^nd^ feature reflects the local geometry of the protein surface. The matrix element (*j,i*) shows the Mann-Whitney U-test p-value in color-code for the two groups of *A_i,j_* : one group of *A_i,j_* was calculated for the attribute type *j* around the surface atom type *i* in the known PPI sites on proteins in the S432 dataset and the other group was calculated for the same attribute type around the non-PPI site atom type *i* in the same dataset. The p-values were calculated with the Mann-Whitney U-test implemented as the function ranksum in MATLAB. Two sets of data were input to the function and the output p-value is the probability for the two distributions of data to be statistically indistinguishable. The plus(+) sign in the matrix element indicates that the averaged feature value for the PPI site atoms is larger than the averaged feature value for the non-PPI site atoms and the negative(−) is the opposite. The panel on the right-hand-side of the matrix shows the distributions of protein surface atoms in PPI sites (blue) and non-PPI protein surfaces (red) against protein atom type. The data were derived from proteins in S432.

Figure 1 demonstrates the validity of the hypothesis above. The physicochemical complementarities around the protein surface atom *i* were simulated with the PDMs of non-covalent interacting atoms and were described with the 32 numerical features calculated with Equation (2) (i.e., *A_i,j_* for interacting atom type *j* = 1∼31 as shown in Table 1; *j* = 32 derived from protein surface geometry). The matrix element (*j,i*) in Figure 1 shows the Mann-Whitney U-test result for the two groups of *A_i,j_*: one group of *A_i,j_* was calculated for the interacting atom type *j* around the surface atom type *i* in the known PPI sites on proteins in the S432 dataset and the other group was calculated for the same interacting atom type around the non-PPI site atom type *i* in the same dataset. The matrix elements showing decreasing p-value substantially less than the statistical threshold of 0.025 are colored in red with increasing depth. These U-test p-values reflect the significant statistical differences in the attributes calculated from the PDMs or surface geometry between the protein surface atoms in known PPI sites and the atoms outside known PPI sites.

**Table 1:** Atom types for 20 natural amino acids in proteins.

ID #	Atom Type	Radius(Å)	Description
1	NH1	1.65	Backbone NH
2	C	1.76	Backbone C
3	CH1E	1.87	Backbone CA (exc. Gly)
4	O	1.40	Backbone O
5	CH0	1.76	Arg CZ, Asn CG, Asp CG, Gln CD, Glu CD
6	CH1S	1.87	Sidechain CH1: Ile CB, Leu CG, Thr CB, Val CB
7	CH2E	1.87	Tetrahedral CH2 (except CH2P,CH2G) All CB
8	CH3E	1.87	Tetrahedral CH3
9	CR1E	1.76	Aromatic CH (except CR1W, CRHH, CR1H)
10	OH1	1.40	Alcohol OH (Ser OG, Thr OG1, Tyr OH)
11	OC	1.40	Carboxyl O (Asp OD1, OD2, Glu OE1, OE2)
12	OS	1.40	Sidechain O: Asn OD1, Gln OE1
13	CH2G	1.87	Gly CA
14	CH2P	1.87	Pro CB, CG, CD
15	NH1S	1.65	Sidechain NH: Arg NE, His ND1, NE1, Trp NE1
16	NC2	1.65	Arg NH1, NH2
17	NH2	1.65	Asn ND2, Gln NE2
18	CR1W	1.76	Trp CZ2, CH2
19	CY2	1.76	Tyr CZ
20	SC	1.85	Cys S
21	CF	1.76	Phe CG
22	SM	1.85	Met S
23	CY	1.76	Tyr CG
24	CW	1.76	Trp CD2, CE2
25	CRHH	1.76	His CE1
26	NH3	1.50	Lys NZ
27	CR1H	1.76	His CD2
28	C5	1.76	His CG
29	N	1.65	Pro N
30	C5W	1.76	Trp CG
31	HOH	1.40	Water

The Table was derived from Laskowski et al [55] with modifications.

### Consistency of the U-tests of the Physicochemical Complementarity Features with Previous Statistical Observations

The U-test results shown in Figure 1 are comparable with general PPI site characteristics from previous statistical observations. Space around the main chain atoms (rows of y = 1∼4) in PPI sites are enriched with higher densities of interacting backbone carbonyl group (x = 2,4) and are neighbored by higher densities of interacting hydrophobic and aromatic carbons (x = 6∼9), while the interacting charged atoms (x = 11, 15∼16, 25∼28) are largely depleted near the main chain atoms in the PPI sites. This is in agreement with the observation that main chain atoms are frequently used in polar interactions in PPI [9]. In particular, the carbonyl oxygen (row of y = 4) is most frequently used in hydrogen bonding in PPI sites [9]. Aliphatic and aromatic carbons (rows of y = 6∼9) in PPI sites are surrounded with high density of interacting aliphatic carbons, aromatic carbons, and atoms from Met and His (x = 6∼9,18∼25, 27∼30), while charged interacting atoms (x = 11, in particular x = 26 for Lys Nz) are also depleted in the PPI sites. But, interestingly, Arg (x = 15,16) remains favorable in the PPI sites near the aromatic carbons (y = 9), in particularly with atoms from Trp (y = 18,24,30). Arg also interacts with carboxyl oxygen (y = 11) more in the PPI sites. This is largely in consistent with the knowledge-based pairwise potentials devised with protein-protein interaction datasets [11], [37]. The sulfur atom of Cys is highly enriched in the PPI sites as interacting atoms (column x = 20), in good agreement with the high interface propensity for Cys [38]. Interacting water molecules (column x = 31) are more dense in PPI sites near polar atoms (y = 1∼4,10∼13,16∼17). This is in consistent with the statistical survey by Rodier et al. [14], suggesting that water molecules in the PPI interfaces play important roles in protein complex formation. The results in the last column (column of x = 32) suggest that PPI sites are more flat or convex than non-PPI surfaces, which is in good agreement with the survey by Jones and Thornton [38]. Although the dataset did not provide enough statistical resolution for rows of y = 18∼30 (see the dataset distribution indicated by the histogram next to the U-test matrix), the consistencies listed above nevertheless suggest that the distribution patterns of the non-covalent interacting atoms predicted with the PDMs on PPI interfaces can provide statistical characteristics in distinguishing the known PPI sites from the other protein surface regions that have not been known to bind to proteins. Since the PDMs were derived from known protein structures, the correlation between the PPI interface features (Figure 1) predicted with the PDMs and those derived from surveys of PPI interfaces also implies that both protein folding and binding are governed by similar energetic principles.

### Atom-based PPI Site Predictions with Machine Learning Models Based on Physicochemical Complementarity Features

**Figure 2:**
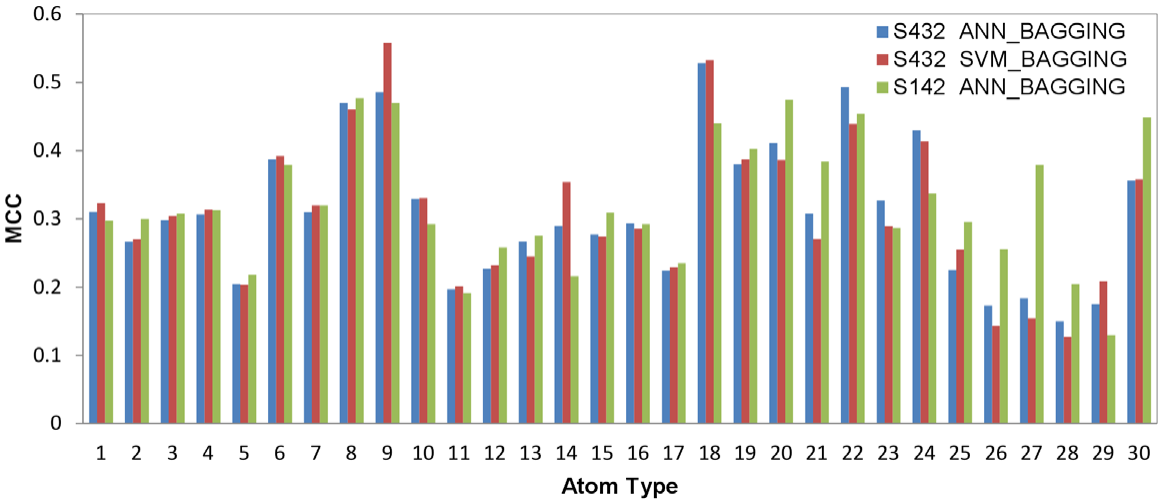
Atom-based prediction accuracies for each of the 30 protein atom types. The x-axis represents indexes for the 30 atom types shown in Table 1. The y-axis shows averaged two-class prediction MCCs from the 5-fold cross validation of the ANN_BAGGING and SVM_BAGGING predictors trained and tested for each of the specific protein atom type with the S432 dataset. The prediction MCCs for the independent test with ANN_BAGGING on the S142 dataset are also shown for comparison.

The results in Figure 1 indicate that the 31 features calculated with PDMs (a set of example PDMs on a protein are shown in Figure S1) and the 32^nd^ feature based on the surface atom local geometry for each of the 30 protein atom types can be used as effective attributes in training machine learning models for PPI site predictions. Machine learning algorithms ANN_BAGGING and SVM_BAGGING were trained for each of the 30 protein surface atom types with five-fold cross validation on the S432 dataset as described in the Methods section. The atom-based MCCs for the five-fold cross validation for each of the atom types are summarized in Figure 2. The benchmarks for the prediction models are shown in Table 2. The differences of the averaged performance for the two machine learning algorithms are essentially indistinguishable (Figure 2 and Table 2), and thus only the ANN_BAGGING models with the best performance were used to benchmark on the S142 dataset as an independent test. The benchmark results on the independent test are compared with the five-fold cross validation in Figure 2 and in Table 2. The benchmark results for the independent test were comparable with the five-fold cross validation results, indicating that the machine learning predictors can be generalized to predict PPI sites on protein surfaces of unknown interaction partners. Figure 2 shows that the prediction models for the atom types from hydrophobic residues with aliphatic and aromatic side chains (atom type index = 8,9,18∼24,30) were predicted with relatively higher accuracies than the atom types from main chain and hydrophilic side chains. This suggests that the core PPI interfaces composed of hot-spot residues (except Arg) are more distinguishable as PPI sites in comparison with the surrounding rim regions populated with higher percentage of polar groups.

**Table 2:** Benchmarks for atom-based PPI site predictions.

Dataset/method	Accuracy	Precision	Sensitivity	Specificity	MCC	F-score
S432/ANN_BAGGING	0.741	0.418	0.569	0.787	0.321	0.481
S432/SVM_BAGGING	0.753	0.434	0.552	0.807	0.330	0.486
S142/ANN_BAGGING	0.732	0.420	0.594	0.771	0.326	0.492

Five-fold Cross validation was performed on the S432 dataset with ANN_BAGGING and SVM_BAGGING. Independent test was performed on the S142 dataset with the best ANN_BAGGING predictors from the five-fold cross validation. The benchmark measurements are defined in Equations (6)∼(11).

**Figure 3:**
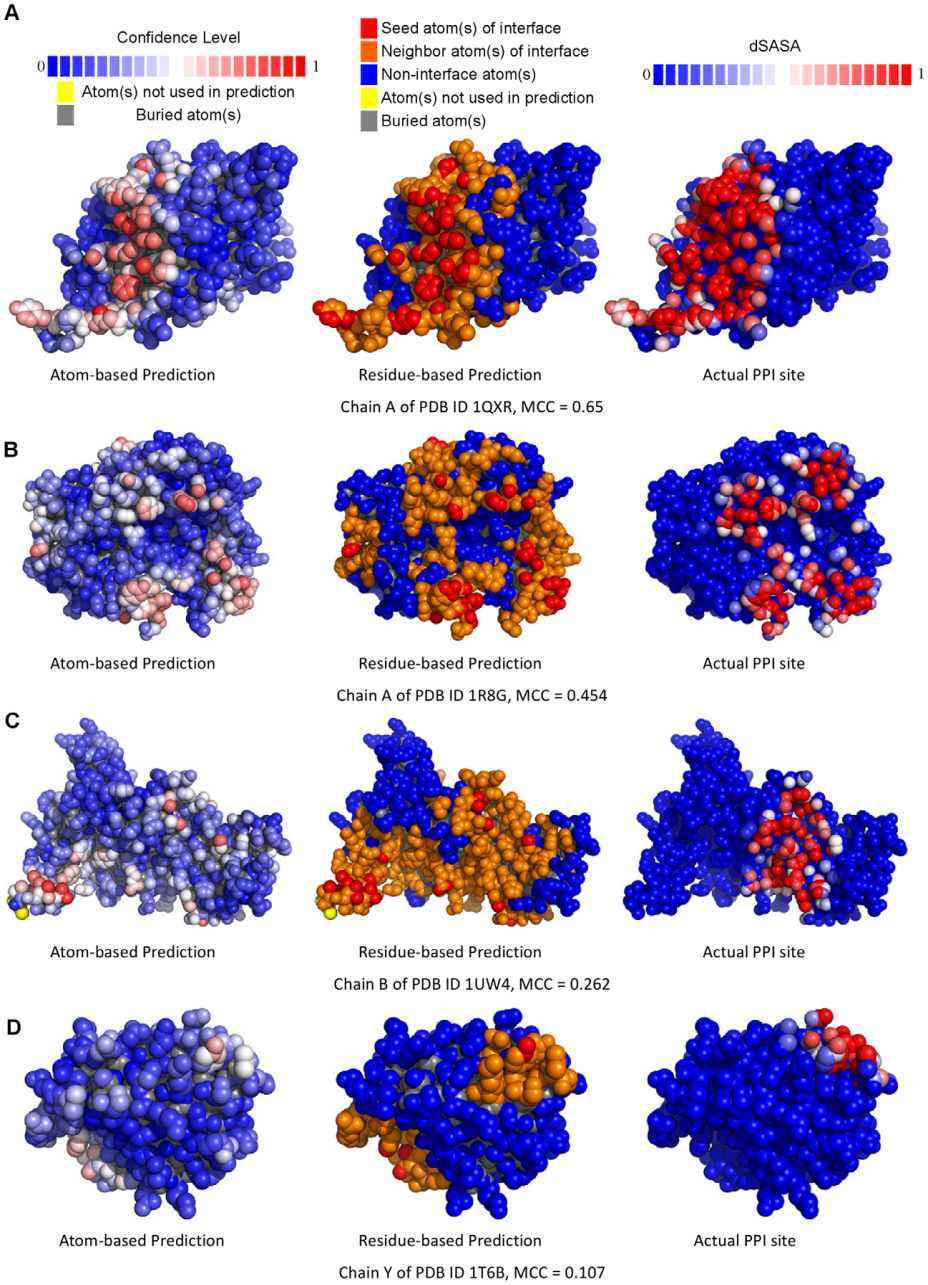
Visualization of prediction results for example protein targets with different prediction accuracy. Panels (A) to (D) demonstrate four proteins with two-class prediction MCC of 0.650, 0.454, 0.262, and 0.107, respectively. The target proteins were selected from the S142 dataset. The predictions were carried out with the best ANN_BAGGING model from the 5-fold cross validation on the S432 dataset. In each panel, the left structure shows the atom-based positive prediction confidence level from blue (confidence level of 0) to red (confidence level 1) for each of the surface atoms. The middle structure shows the residue-based predictions. The atoms colored in red were predicted with confidence level greater than 0.6; atoms in orange are the atoms belonging to the residues in the residue-based PPI site prediction but the prediction confidence levels are less than 0.6. The right-hand-side structure shows the actual PPI sites: the PPI surface atoms are colored according to dSASA (see Equation (4)) from blue (dSASA of 0 for atoms not involving in PPI) to red (dSASA of 1 for atoms completely buried in the protein complex). The color-codes are shown at the top of the figure. Atoms not used in prediction (colored in yellow) belong to residues with incomplete *phi* and *psi* angles, as in the N-termini or C-termini of proteins. The non-surface atoms are colored in gray. The complete prediction results can also be viewed in color-coded 3-D protein structures from the web server http://ismblab.genomics.sinica.edu.tw/> benchmark >protein-protein.

The PPI surface patches on protein surfaces were predicted by combining the machine learning predictions for each of the surface atoms. The activity (probability) outputs from the machine learning models were first converted into prediction confidence levels so that surface atoms with high confidence level predictions can be clustered into surface patches as PPI sites (see Methods). Figure 3 shows a few examples of protein surface PPI site predictions, compared side-by-side with actual PPI sites, with various prediction accuracies (residue-based MCC ranging from 0.7 to 0.1). The complete set of prediction results on the proteins from the training and test sets can be viewed with interactive 3-D structural presentation from the web server http://ismblab.genomics.sinica.edu.tw/> benchmark >protein-protein.

### Residue-based PPI Site Predictions with Machine Learning Models Based on Physicochemical Complementarity Features and the Comparison of the Prediction Benchmarks Among Comparable Predictors

**Figure 4:**
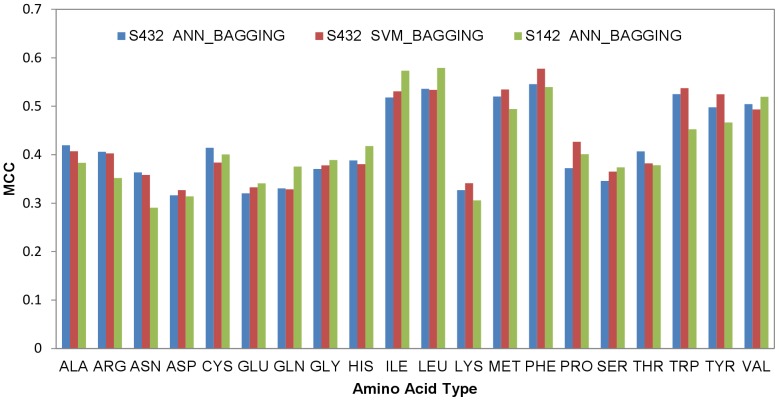
Residue-based two-class prediction MCCs for each of the 20 natural amino acid types. The MCCs were calculated as the average value from the 5-fold cross validation with the ANN_BAGGING and SVM_BAGGING predictors on the S432 dataset. The independent test MCCs with the best ANN_BAGGING predictors from the 5-fold cross validation on the S142 dataset are also shown for comparison.

Residues in the predicted PPI surface patches were predicted based on the atom-based PPI site predictions (see Methods) and were benchmarked with the residues in actual PPI sites. The example residue-based PPI site predictions are also compared side-by-side with the atom-based predictions and the actual PPI sites in Figure 3. The residue-based MCC for each of the amino acid types are shown in Figure 4. The accuracy benchmarks are summarized in Table 3. Again, the two machine learning algorithms are comparable in terms of the prediction performance (Table 3 and Figure 4). The generalized prediction capacity of the ANN_BAGGING models was demonstrated with the results of the independent test, for which the results were essentially indistinguishable from the results of the five-fold cross validation as shown in Figure 4 and Table 3. Accuracy benchmarks for each protein from the cross validation (with ANN_BAGGING and SVM_BAGGING) and from the independent test (with ANN_BAGGING) are listed in Table S2, S3, and S4 respectively. The prediction results can also be viewed in color-coded 3-D protein structures from the web server http://ismblab.genomics.sinica.edu.tw/> benchmark >protein-protein.

**Table 3:** Benchmarks for residue-based PPI site predictions.

Dataset/method	Accuracy	Precision	Sensitivity	Specificity	MCC	F-score	TP/TN	FP/FN
S432/ANN_BAGGING	0.759	0.512	0.662	0.791	0.420	0.578	13970/50458	13300/7118
S432/SVM_BAGGING	0.748	0.495	0.709	0.761	0.424	0.583	14953/48528	15230/6135
S142/ANN_BAGGING	0.753	0.519	0.677	0.779	0.423	0.588	4060/13298	3763/1934

Five-fold Cross validation was performed on the S432 dataset with ANN_BAGGING and SVM_BAGGING. Independent test was performed on the S142 dataset with the best ANN_BAGGING predictors from the five-fold cross validation. The benchmark measurements are defined in Equations (6)∼(11).

**Figure 5:**
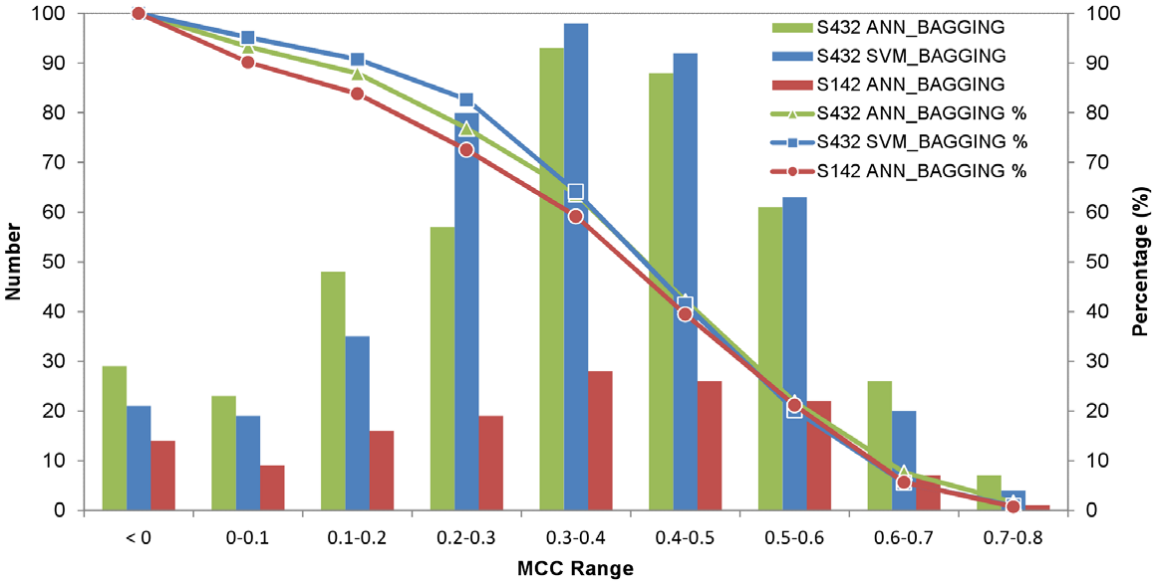
The distributions of the prediction accuracies on the 5-fold cross validations and on the independent test. The y-axis on the left-hand-side of the panel is associated with the histograms, showing the distributions of the number of proteins in the 5-fold cross validations or in the independent test that were predicted with the MCC within the MCC range shown in x-axis. The y-axis on the right-hand-side of the panel is associated with the curves connecting the dots representing the cumulative percentage of the proteins predicted with the residue-based MCC shown in the x-axis. The 5-fold cross validations were carried out with the ANN_BAGGING and SVM_BAGGING predictors on the S432 dataset; the independent test was carried out with the best ANN_BAGGING predictors from the 5-fold cross validation on the S142 dataset.

The distribution of prediction accuracy for proteins in the S432 and S142 dataset are shown in Figure 5, for which the overall benchmark results are summarized in Table 3. The independent test (MCC = 0.423) for the residue-based PPI site predictions, as shown in Table 3, can be compared with previous publications based on the same training and test datasets. Porollo et al. [27] developed SPPIDER predictor for PPI site residue predictions with essential the same training and test datasets based on a combination of structural and sequence features. Their residue-based prediction MCC for the independent dataset is 0.42. In another work, a detailed analysis of the sequence and structural attributes on the same training and test datasets has concluded that the best performance for independent PPI site residue-based predictions yielded MCC of 0.37 on the same test set [3]. By taking away the evolutionary information from the prediction inputs, the MCC dropped to 0.34. Hence, the PPI site predictions based on the physicochemical complementarities derived from the PDMs on the protein surfaces are currently the best structure-based predictors judging by the MCC of the residue-based predictions. The performance of the predictors developed in this work would be further improved if the evolutionary information of the query proteins is to be integrated into the prediction algorithms.

Table 4 compares the predictions results of a set of 17 test proteins with both bound and unbound structures. As expected, the predictions with the unbounded structures are less accurate than the bound structures. The PPI site predictions with unbound structures (MCC = 0.326) are about the same in prediction accuracy as those by Porollo et al. (MCC = 0.32), while the predictions with bound structures (MCC = 0.364) are also the same as those by Porollo et al. (MCC = 0.36) [27]. Accuracy benchmarks for each of the protein in S17a are shown in Table S5. The prediction results can also be viewed in color-coded 3-D protein structures from the web server http://ismblab.genomics.sinica.edu.tw/> benchmark >protein-protein.

**Table 4:** Residue-based benchmark comparison between the bound state and unbound state of the proteins in the S17a dataset.

Protein structure	Accuracy	Precision	Sensitivity	Specificity	MCC	F-score	TP/TN	FP/FN
Unbound state	0.767	0.327	0.626	0.790	0.326	0.430	275/2133	566/164
Bound state	0.777	0.402	0.613	0.811	0.364	0.486	322/2049	479/203

Unbound state performances are based on the prediction results with the best ANN_BAGGING predictors from the 5-fold cross validation. Bound state performances are based on corresponding protein structures from the S142 dataset. The benchmark measurements are defined in Equations (6)∼(11).

Furthermore, the prediction capacities of the predictors devised in this work have been compared with public domain servers using protein structures as input. The structures from the independent test set S58 (non-redundant protein complex structures from entries published in 2011, see Methods) were submitted to comparable public domain servers to predict PPI sites. The residue-based predictions were benchmarked. The overall MCC of 0.40 of the ANN_BAGGING prediction is consistent with the benchmark results shown in Tables 3 and 4. The detailed prediction results are shown in Table S6. The prediction results can also be viewed in color-coded 3-D protein structures from the web server http://ismblab.genomics.sinica.edu.tw/> benchmark >protein-protein. Table 5 shows the comparison of the prediction accuracies of the method in this work with those from the PredUs [28], [39] server, which had the best performance, judging by the prediction results of the test set S58, among the comparable prediction servers accessible in the public domain. The prediction accuracy benchmarks shown in Table 5 are comparable between the two methods.

**Table 5:** Benchmarks for residue-based PPI site prediction for proteins in the S58 dataset.

Method	Accuracy	Precision	Sensitivity	Specificity	MCC	F-score	TP/TN	FP/FN
PredUs	0.785	0.455	0.576	0.835	0.377	0.508	1321/8025	1584/974
ANN_BAGGING	0.777	0.446	0.654	0.806	0.403	0.530	1500/7744	1865/795

PredUs [28], [39] (http://bhapp.c2b2.columbia.edu/PredUs/) was unable to predict chain A of PDB ID 3myo and chain A of PDB ID 3ulc due to lack of “structural neighbors”. For the rest of the queries in PredUs predictions, the structural neighbor with PDB ID identical to the query protein was removed and the remaining structural neighbors were used for prediction. The PredUs predictions were compared with ANN_BAGGING prediction results as shown in the Table (detailed results are shown in Table S6). Only the prediction results for the protein surface residues (defined in Methods) were used for benchmarking. The benchmark measurements are defined in Equations (6)∼(11).

### Contribution of the Attributes to the Machine Learning Prediction Accuracy

**Figure 6:**
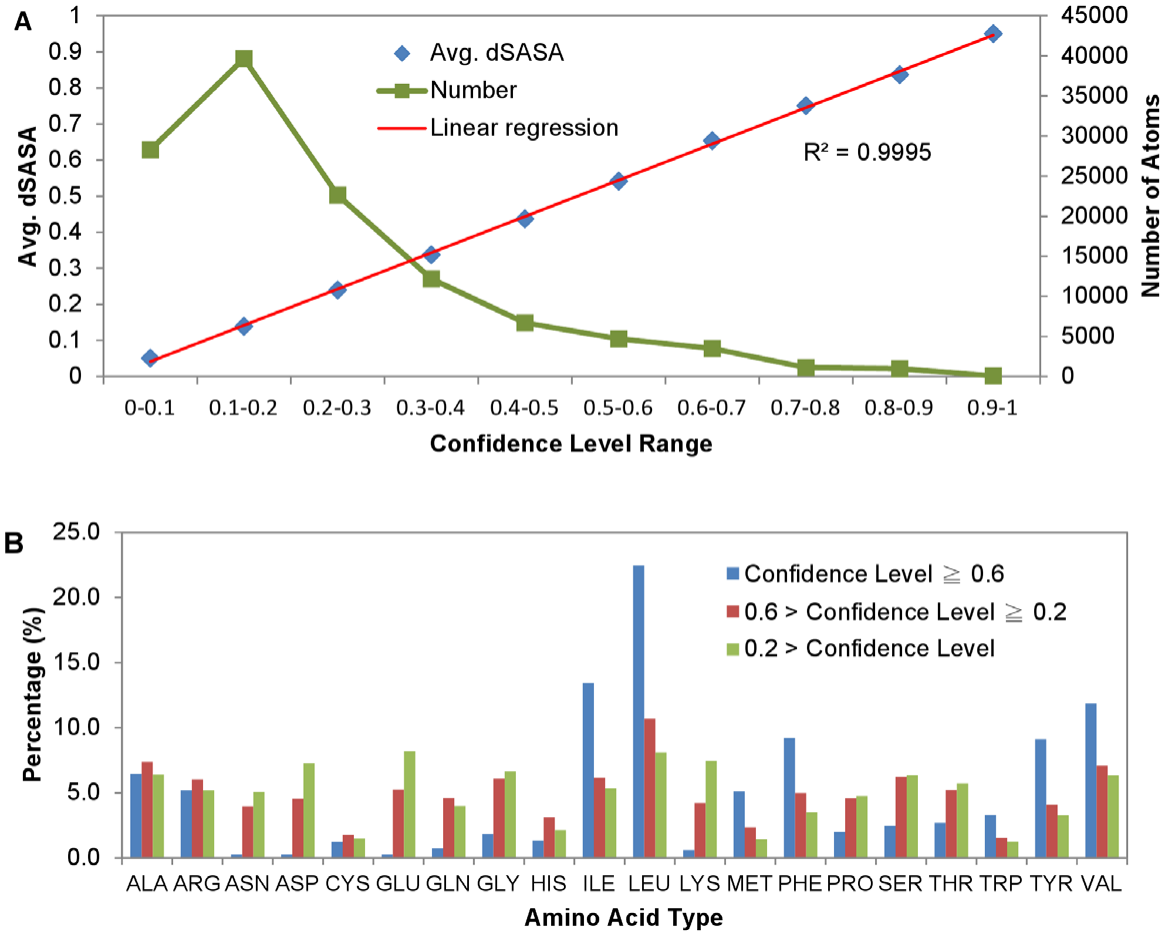
Correlations of PPI site prediction confidence level to atomic burial in protein complexes and to amino acid type. (A) Atom-based prediction confidence level range (shown in the x-axis of the panel) is correlated to the averaged burial level (measured by dSASA (Equation (4)) of the sub-group of atoms in the protein complexes predicted within the confidence level range. The correlation is shown by the diamond symbols, corresponding to the y-axis on the left-hand-side of the panel. The distribution of the atom-based predictions as shown by the curve, corresponding to the y-axis on the right-hand-side, is plotted against the prediction confidence level range in the x-axis. The data were derived from the independent test with the ANN_BAGGING predictors on the S142 dataset. (B) The histograms in this panel show the distributions of amino acid types in three groups of protein surface residues with various atom-based prediction confidence level ranges. The first group of residues contained atom-based prediction confidence level ≥ 0.6 for at least one atom in each of the residues. The second group of residues contained atom-based prediction confidence level between 0.6 and 0.2 for at least one atom in each of the residues. The third group of residues contained atom-based prediction confidence level less than 0.2 for at least one atom in each of the residues. The distribution of the percentage of the amino acid types in each of the three groups is shown by a histogram in the panel. The data were derived from the independent test of the best ANN_BAGGING predictors on the S142 dataset.

**Figure 7:**
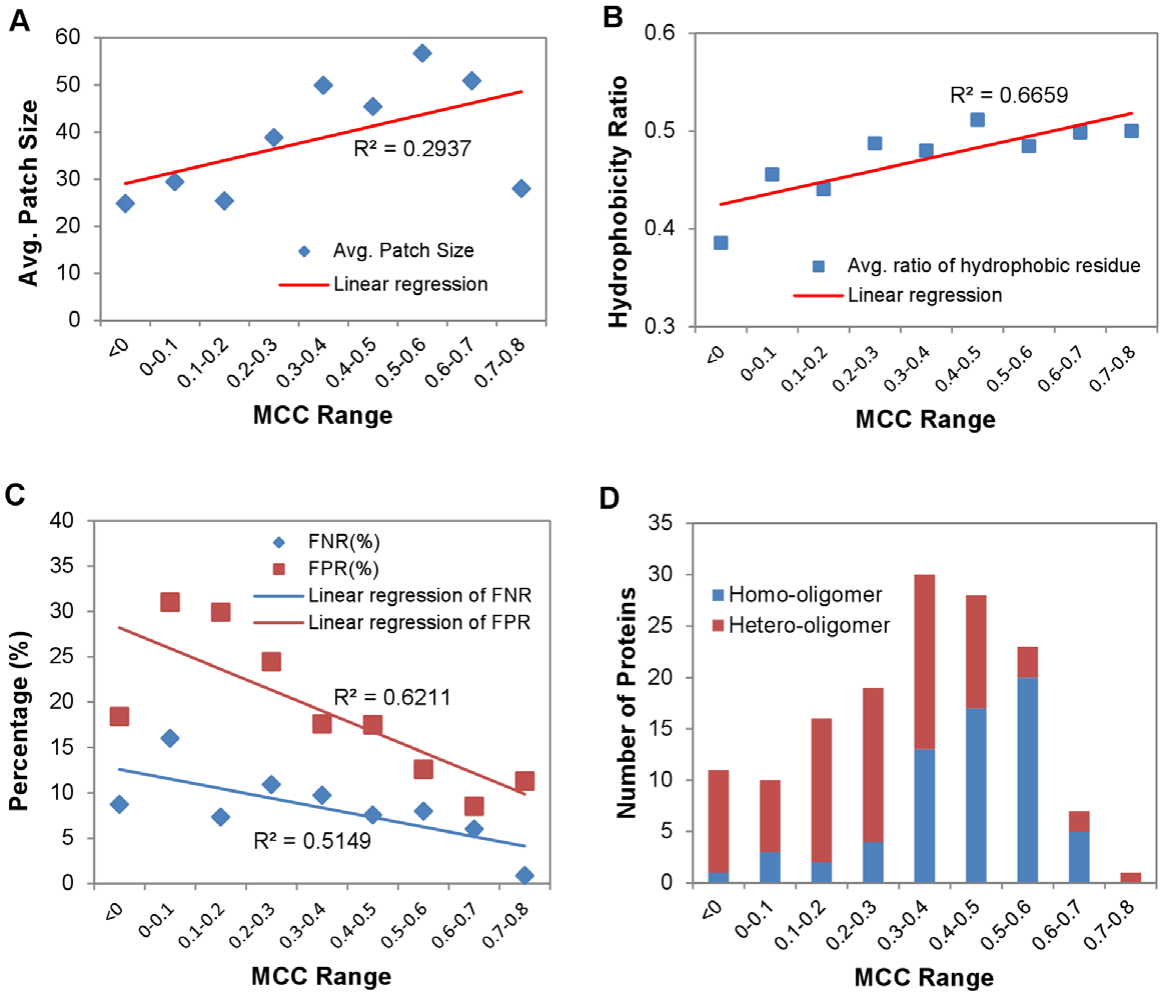
Correlations of PPI site prediction accuracy to PPI features. The data were derived from the independent test of the best ANN_BAGGING predictors on the S142 dataset. (A) PPI patch size averaged over the proteins predicted within the residue-based MCC range shown in the x-axis is plotted against the MCC range. Patch size is defined as the number of residues in the actual PPI-site. (B) PPI patch hydrophobicity ratio averaged over the proteins predicted within the residue-based MCC range shown in the x-axis is plotted against the MCC range. Hydrophobic residues include Ala, Cys, Ile, Leu, Met, Phe, Pro, Tyr, Trp, and Val. Ratio of hydrophobic residues was computed as the number of hydrophobic residues in the PPI-site divided by the total number of residues in the site. (C) False negative ratio (FNR) and false positive ratio (FPR) averaged over the proteins predicted within the reisude-based MCC range shown in the x-axis is plotted against the MCC range. FNR was calculated as (FN/(TP+TN+FP+FN))×100%, and FPR was calculated as (FP/(TP+TN+FP+FN))×100%. The TP (true positive), TN (true negative), FP (false positive), and FN (false negative) were derived from residue-based predictions. (D) Distributions of homo-oligomers and hetero-oligomers are plotted against the residue-based MCC range. The detailed assignments of the PPI type for the proteins in the S142 dataset are shown in Table S4. MCC was calculated based on residue-based predictions.

**Figure 8:**
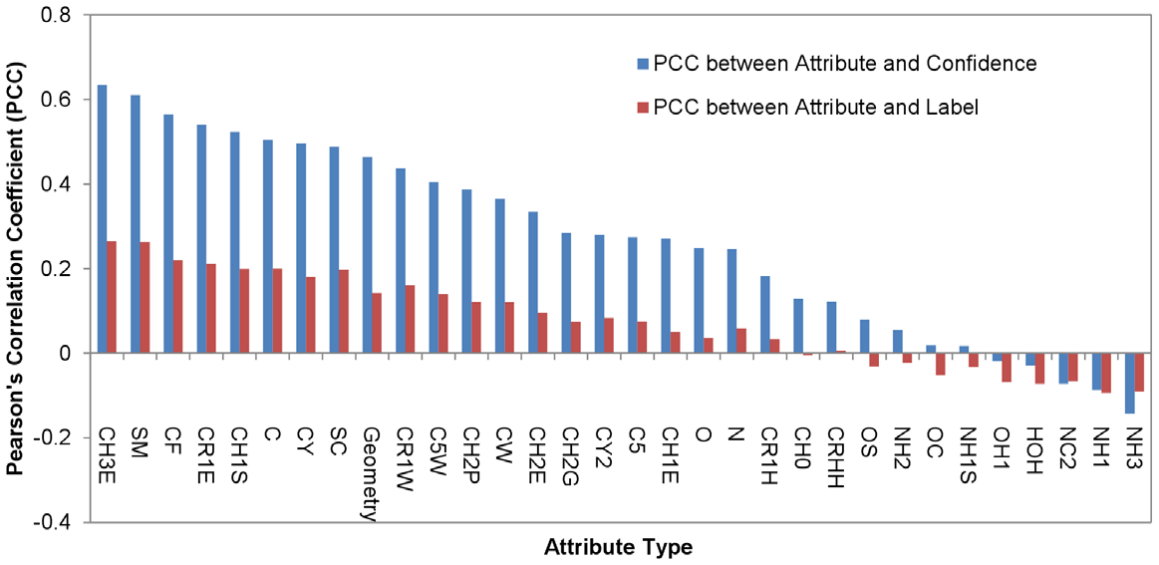
Ranking of the attributes derived from PDMs. Each of the surface atoms *i* in the S142 dataset has a confidence level on the prediction of the atom to be in a PPI site. This prediction confidence level is correlated to various extents with the 32 attributes (*a_i,j_* (*j* = 1∼32) as shown in Equation (3)), which were used as inputs for the machine learning predictors in making the predictions. The blue histogram shows the correlations between prediction confidence levels and attributes derived from concentrations of PDMs. The Pearson’s correlation coefficients, which are the measurements for the linear correlations between the prediction confidence level and the attributes, are shown in the y-axis. The x-axis shows the feature types (Table 1), each of which corresponds to one of the *a_i,j_*. The red histogram shows the Pearson’s correlation coefficients between the positive (1 for PPI site atoms) or negative (0 for non-PPI site atoms) assignments for protein surface atoms and the attribute values for the protein surface atoms.

**Figure 9:**
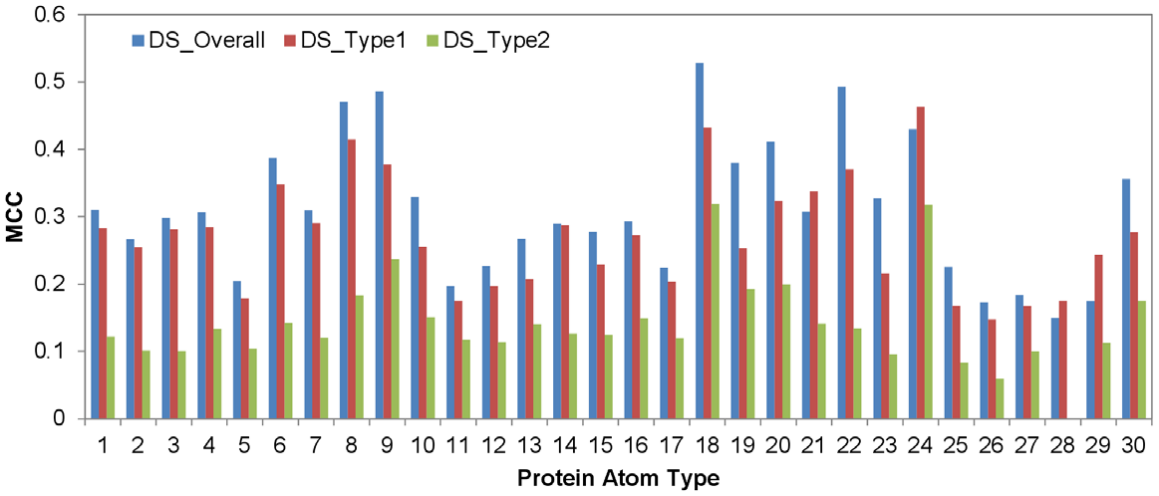
Atom-based MCC comparison among machine learning models trained with the DS_Overall, DS_Type1, and DS_Type2 dataset. DS_Type1 and DS_Type2 are variants of S432 dataset. The former has all type 1 PPI sites (44% hydrophobic, 47% hydrophilic, and 9% aromatic residues) labeled as positive and the rest labeled as negative; the latter has all type 2 PPI sites (25% hydrophobic, 66% hydrophilic, and 9% aromatic residues) labeled as positive and the rest labeled as negative. DS_Overall is the original version of S432 with all PPI sites (type 1 and type 2 PPI sites) labeled as positive. Five-fold cross validation was performed with DS_Type1 and DS_Type2 based on the same procedures described in Methods section. The parameters used for training remained the same, except for the increased bag number of 20 in an attempt to alleviate the class imbalanced problem since fewer positive cases were labeled in DS_Type1 and DS_Type2.

Figure 6 shows that the protein surface atoms predicted with high confidence level are more buried in the actual PPI sites and are mostly from hydrophobic and aromatic residues. Figure 6A shows the linear correlation between the prediction confidence level and the burial level – the higher the prediction confidence level for a surface atom to be in a PPI site, the more buried for the atom to be in an actual PPI interface. As expected, as shown in Figure 6B, the residues for which the atoms were predicted with confidence level ≥ 0.6 were mostly hydrophobic residues as Ile, Leu, Met, Phe, Tyr, and Val. The residue atoms predicted with modest confidence level between 0.2 and 0.6 are not as hydrophobic as those predicted with high confidence level (Figure 6B), and are not as hydrophilic as those predicted with confidence level less than 0.2 (Figure 6B). These results imply that the PPI sites with less prominent hydrophobic cores are less likely to be predicted with high accuracy. Indeed, this implication is validated in Figures 7, 8, and 9.

Figure 7 shows that the prediction accuracy deteriorates as the actual PPI sites become smaller in size (Figure 7A) and less hydrophobic in amino acid composition (Figure 7B). Figure 7C shows that the false positive ratios (FP/(TP+TN+FP+FN)) increases with greater rate than the false negative ratios (FN/(TP+TN+FP+FN)) as the MCC decreasing. This suggests that the decreasing accuracies of the PPI site predictions were resulted more from increasing false positive predictions. It is questionable as to whether the false positive predictions are truly false positives – these false positive PPI sites could be perceived as potential PPI sites that have not been validated experimentally. By comparing Figure 7D with Figure 7A∼7C, it is evident that homo-oligomers, each of which is formed with a single polypeptide chain, have larger PPI interfaces (Figure 7A) and with more hydrophobic residues in the PPI sites (Figure 7B), and thus were predicted with less false positives and false negatives (Figure 7C) and higher accuracy (Figure 7D). In contrast, interfaces in hetero-oligomers are relatively smaller and more hydrophilic and are more difficult to be predicted accurately than the interfaces in homo-oligomers.

The blue histogram in Figure 8 shows the Pearson’s correlation coefficients between the prediction confidence level and the attribute types (*j* = 1∼32) calculated in Equation (3). The prediction confidence-attribute correlations are strongly dependent on the attribute type. As shown in the histogram, increasing prediction confidence levels are linearly and positively correlated with increasing values of the attributes derived from the aliphatic and aromatic carbons, suggesting that the PDM concentrations of these interacting atoms are greater around the protein surface atoms that are predicted to be in the PPI sites with high prediction confidence level. This is in good agreement with the notion that PPI interface cores are similar to protein interiors in hydrophobic amino acid composition, and thus are predicted with higher accuracy and confidence level. Attributes of hydrophilic atoms (NH3, NH1, NC2, OH1, NH1S, OC, NH2, OS, see Table 1) are not correlated with prediction confidence level (blue histogram in Figure 8), suggesting that the patterns of the PDMs derived from these hydrophilic atoms are indistinguishable between the PPI sites and the non-PPI sites, and thus contribute little to the PPI prediction accuracy. This is in agreement with the notion that some regions of the PPI sites are as hydrophilic as the protein surface in general.

The red histogram in Figure 8 shows the Pearson’s correlation coefficients between the positive (1 for PPI site atoms) or negative (0 for non-PPI site atoms) assignments for protein surface atoms and the attribute values for the atoms on the protein surface. In theory, attributes (x-axis) correlated to the positive or negative assignments with higher correlation coefficients (y-axis) should contribute statistically more weight in prediction accuracy. This expectation has been validated by the almost identical trends in comparing the red histogram with the blue histogram shown in Figure 8, indicating that indeed the contributions of the attributes to the prediction accuracy as indicated in the blue histogram are in good agreement with the statistical expectations shown in the red histogram.

Moreover, comparison of Figure 1 and Figure 8 shows clearly the extent of contribution of the attributes to the prediction accuracy. As shown in Figure 1, the attributes (shown in the x-axis) with larger p-values from the U-tests (i.e., the columns for which the colors approach the blue end), such as attributes 1, 5, 10, 11, 12, 15, 16, 17, 27, 28, 31 (these attributes are denoted as NH1, CH0, OH1, OC, OS, NH1S, NC2, NH2, CR1H, C5, HOH respectively as defined in Table 1 and shown in Figure 8), are all correlated poorly with prediction confidence level (blue histogram in Figure 8) and PPI site assignment (red histogram in Figure 8). This result suggests that the U-tests shown in Figure 1 are strong predictors for the ranking of the contributions of the attributes to the machine learning prediction capability.

### Training of the Machine Learning Models with Subsets of Protein-protein Interaction Interfaces

The results above suggested a possibility that the prediction of PPI sites with more hydrophilic residues might be improved with a training set containing only the hydrophilic interfaces. This possibility was tested by clustering the PPI sites of the proteins in the training set into two groups with distinguishingly different residue compositions. Type 1 PPI sites are centered on a representative surface patch with equal distribution of the hydrophobic and hydrophilic residues (44% hydrophobic, 47% hydrophilic, and 9% aromatic residues) and type 2 PPI sites are centered on a representative surface patch with more hydrophilic residues (25% hydrophobic, 66% hydrophilic, and 9% aromatic residues). Hydrophobic residues are Ala, Pro, Leu, Ile, Met, Cys, and Val; aromatic residues are Phe, Tyr, and Trp. The rest of the amino acid types are hydrophilic. Two datasets derived from S432, named DS_Type1 and DS_Type2, were generated with atoms labeled as positive for only type 1 PPI sites and type 2 PPI sites, respectively. Cross validation benchmark procedures as described above were applied to the two datasets. Figure 9 shows that prediction models trained and tested with type 1 PPI sites were more accurate than those trained and tested with type 2 PPI sites, suggesting that PPI sites with hydrophobic or aromatic cores are predicted with substantially higher accuracy than the PPI sites composed of mostly hydrophilic residues. Figure 9 also suggests that training two sets of prediction models with two sets of PPI sites did not improve prediction accuracy. As shown in the Figure, the prediction models trained with the overall data set are not inferior to the predictions models trained by either of the datasets. Evidently, few rules can be learned statistically on the polar interactions in PPI sites to improve the PPI site prediction accuracy.

Taken together, the PPI sites in homo-oligomers are usually formed with large interface area with hydrophobic interface cores and hydrophilic peripheral areas. These PPI sites can be predicted with reasonable accuracy with the methodology developed in this work. As the PPI sites become smaller and more hydrophilic, as in the interfaces of some hetero-oligomers where hydrophobic cores become less prominent, the accuracy of the PPI site prediction deteriorates. In some of these interfaces, the rim regions make the dominant parts of the PPI sites and the interface cores become increasingly insignificant as the interface size decreases [10], [11], [15], [38]. The PPI sites in these complexes are increasingly indistinguishable from the non-PPI protein surfaces, and as a result, the machine learning algorithms are less effective in identifying these PPI sites. It seems that the polar interfaces in some transient PPIs emphasize a different set of energetic terms distinguishable from those for the homo-oligomers, and that the PDMs derived from protein interiors fall short to account for the polar interactions in the transient PPI sites. Increasing understanding of the polar interactions involving perhaps water-mediated terms [40] on protein surfaces could contribute in establishing a better prediction method for polar PPI sites predictions.

### Summary

In summary, PPI sites on proteins of known structures can be predicted with accuracy to an extent based on the physicochemical complementarity derived from PDMs on protein surfaces. Although the PDMs, which describe the three-dimensional distributions of non-covalent interacting atoms on protein surface, were derived from protein structures, the physicochemical complementarity in PPI interfaces can be faithfully reproduced with the numerical features derived from the PDMs, indicating that protein folding and binding are governed by similar energetic principles. The predictions based on these PDM-recreated physiochemical complementarity features on protein surfaces are among the best in PPI site predictions with known protein structures. In particular, the hydrophobic cores of the PPI sites are more likely to be correctly predicted. As the PPI sites become smaller in size and less hydrophobic in amino acid composition, the prediction of these PPI sites became increasingly difficult. The difficulties could not be overcome by training the predictors with the subset of PPI sites characterized with more hydrophilic residues in the PPI sites. The PPI site predictions are nevertheless likely to be further improved with additional understanding of polar and water-mediated interactions in protein-protein recognitions.

## Methods

### Constructing Three-dimensional Probability Density Maps (PDMs) for Non-covalent Interacting Atoms on Protein Surfaces

Probability density maps (PDM) constructed with protein non-covalent interacting atoms from known protein structures have been described previously [41]. The detailed method for the PDM construction is described in Text S1. The 31 atom types from proteins and crystal water are listed in Table 1. In order to keep PDM high in information content and low in noise from irrelevant interactions, non-interacting atomic pairs were eliminated with a filter system based on the work by McConkey et al. [42] (Table S1). A set of 31 PDMs on a protein as examples are shown in Figure S1. Interactive 3-D graphic presentation of the PDMs can be viewed from the web-server http://ismblab.genomics.sinica.edu.tw/> gallery.

### PDM-based Attributes as Inputs for Machine Learning Algorithms

One machine learning model was trained for each of the 30 protein atom types (atom types 1∼30 in Table 1). The input attributes for each of the machine learning models were calculated from the PDMs on the protein surface. For each protein atom *i*, the PDM values for interacting atom type *j* associated with the grids within 5 Å radius centered at the atom *i* were summed and associated with the center of the atom as *S_i,j_*:
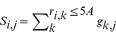
(1)where *r_i,k_* is the distance between atom *i* to a grid point *k*; *g_k,j_* is the PDM value of atom type *j* at grid point *k*.

The distance-weighted sum (*A_i,j_*; *j* = 1∼31 for the 31 interacting atom types 1∼31 in Table 1) over *S_k,j_* for atoms *k* within 10Å from atom *i* was calculated with Equation (2).
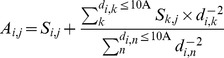
(2)where *S_i,j_* is defined in Equation (1); *d_i,k_* is the distance between atom *i* and atom *k; d_i,n_* is the distance between atom *i* and atom *n*. *A_i,j_* encodes complementarity information on interacting atom type *j* over a circular protein surface patch centered at atom *i* on the protein. The 32^nd^ attribute for the atom *i* was the fraction of the space not occupied by the van der Waals volume of the protein in the 10 Å sphere centered at the atom *i*.

The attributes *a_i,j_* (*j* = 1∼31 for the 31 interacting atom types in Table 1, and *j* = 32 for the geometry attribute) associated with protein atom *i* as inputs for the machine learning algorithms were scaled between 0 and 1. Equation (3) shows the calculation of *a_i,j_* from *A_i,j_* (*j* = 1∼32):if *A_i,j_* > *M_max,j_* then *a_i,j_* = 1; otherwise, if *A_i,j_* < *M_min,j_* then *a_i,j_* = 0; otherwise,
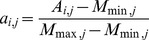
(3)where Mmax,j is the median of the distribution of the maximal Ai,j from each of the proteins in the S432 non-redundant protein data set (see below) and Mmin,j is the median of the distribution of the minimal Ai,j of the same dataset. Figure S2 shows the plots of Mmin,j and Mmax,j against the 32 attribute types.

### Datasets

Three datasets were downloaded from the SPPIDER website [27]. These data sets include a training set, S435, a test set, S149, and an unbound dataset, S21a. We made several modifications to the datasets as the following: Chain A of PDB ID 1GY9 was removed because the complex described in Elkins et al. [43] could not be found in the current PDB. Chain A and C of PDB ID 1DF9 were removed since the records were obsolete. By removing the three proteins from S435, we obtained a dataset named S432. For the independent test set, seven proteins were removed for the following reasons: Chain A and B of PDB ID 1NRJ were removed because they already existed in the training set. Chain K and L of PDB ID 1N13, chain D of PDB ID 1NF3, and chain D of PDB ID 1L9W were removed because they were identical to chain A and B of PDB ID 1N13, chain C of PDB ID 1NF3, and chain A of PDB ID 1L93 in the training set, respectively. Chain A of PDB ID 1PUG was removed because it was a hypothetical protein. By removing seven proteins from S149, we obtained the independent test set S142. For the unbound dataset, chain A of PDB ID 1GQN and chain A of PDB ID 1RZX were removed because they were identical to chain A of PDB ID 1L93 and chain C of PDB ID 1NF3 in the training set, respectively. Chain A of PDB ID 1J8B was removed because it was a hypothetical protein. Chain A of PDB ID 1NX6 was removed because its interface was engineered with two insertions compared to its bound state protein, chain A of PDB ID 1T4B. By removing the four proteins from S21a, we obtained the unbound dataset S17a.

In order to test the performance of the predictors devised in this work with other comparable predictors in the public domain, we downloaded protein complex structures released in 2011 from PDB website with the following criteria: 1) resolution is less than 3.0 Å, 2) chain length is greater than 100 amino acids, 3) entry has two subunits in biological ensemble, 4) entry does not have DNA, RNA, ligands, or modified residues, 5) there is no missing atom in the PDB files, and 6) pairwise sequence identity between any two proteins is less than 30%. The protein chains were further filtered to ensure none of them share greater than 30% sequence identity to proteins in S432, the training set used in this work as described in the previous paragraph. This set of 58 protein chains, denoted S58, was used as the test set for the comparison of prediction capabilities among different PPI site prediction servers.

### Determining Biologically Relevant PPI Sites

All PDB chain records in the three datasets above were checked with PQS (protein quaternary structure) server [44] to determine the biologically relevant PPI sites, so that crystal packing interfaces were removed and biological units were reassembled from asymmetric units. PPI sites at atomistic level were defined with the difference of solvent accessible surface area (*dSASA*) upon complex formation by NACCESS software [45] as below.
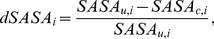
(4)where *SASA_u,i_* and *SASA_c,i_* are the SASA of atom *i* in the uncomplexed and complexed state, respectively. An atom *i* was defined as a PPI site atom when *dSASA_i_* is greater than 0.

### Artificial Neural Network (ANN)

The standard feed-forward back-propagation neural network [46] was used to learn the weight of the network by employing gradient descent to minimize the sum of squared error between the network output values and the target values. The input layer consisted of 32 nodes for the input attributes described in Equation (3). The only hidden layer contained 15 nodes. The output layer had a single node with the activity value between 0 and 1, matching the negative and positive cases respectively for the atoms in PPI sites as defined in Equation (4). Sigmoid function, denoted as *sf*, was used as the transfer function for the hidden and output layers of of the ANN network.
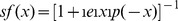
(5)


As an alternative to the more common Levenberg-Marquardt back-propagation training algorithm [47], the very high speed resilient back-propagation (RPROP) training technique was used [48], [49]. Resilient propagation is capable of automatic adjustment for learning rate and momentum. It has the advantage of faster convergence while requiring less manual determination of network parameters. Each of the ANN models was trained for 1000 iterations. During training, the model was tested on validation set after every ten training iterations. The number of training iteration which yielded the best MCC (see below for MCC definition) on the validation set was used to determine the predictors. The open source java-based neural network library ENCOG was used for the implementation.

### Support Vector Machines (SVM)

The details of the standard SVM methodology implemented with LIBSVM package has been described previously [35]. In brief, the SVM is a two-class classification approach with a maximized-margin hyperplane, where margin is the distance from the separating hyperplane to the closest data point [50], [51]. The cost (c) and gamma (γ) parameters of the SVM were optimized with grid searching for the optimal MCC using only the training dataset.

### Bootstrap Aggregation (BAGGING)

Since non-binding atoms in the training set greatly outnumbered binding atoms, ordinary machine learning algorithms would produce learning biases without suitable treatment. The methodology included multiple predictors to produce an ensemble of prediction results [52]. Each individual classifier in the predictor ensemble was trained with a different sampling (bag) of the training set, and the final prediction was calculated by averaging with equal weight the output values from the predictors [53]. In each bag, all of the positive cases were included, along with randomly sampled negative cases that were 1.5 times as many as positive cases. The bag number was set to ten, which balanced the need for effectiveness and training efficiency. All the ten bags were used to train either a set of ANN models (named ANN_BAGGING) or a set of SVM models (named SVM_BAGGING).

The machine learning parameters can be downloaded from the web-server http://ismblab.genomics.sinica.edu.tw/>Download. The attributes *a_i,j_* (*j* = 1∼31 for the 31 interacting atom types in Table 1, and *j* = 32 for the geometry attribute) associated with protein atom *i* for all proteins in the data sets S432, S142, S17a, S58 can be downloaded from the same web-server.

### Prediction Capacity Benchmarking

The prediction capabilities of the machine learning models were benchmarked by accuracy (Acc), precision (Pre), sensitivity (Sen), specificity (Spe), F-score, and Matthews correlation coefficient (MCC) [54].
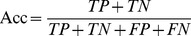
(6)

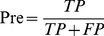
(7)

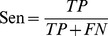
(8)

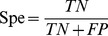
(9)

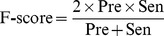
(10)

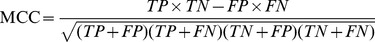
(11)where TP is the number of true positives; TN the number of true negatives; FP the number of false positives; and FN the number of false negatives. Sensitivity (also known as recall) can be viewed as a measurement of completeness, whereas precision is a measurement of exactness or fidelity. MCC, as a measurement of the quality of two class classifications (positive and negative), is generally regarded as a balanced measurement which can be used even if the classes are of very different sizes. Its value ranges between −1 and 1; random correlation gives MCC of zero while perfect correlation yields MCC of one.

### Prediction Confidence Level

Prediction activity (ANN_BAGGING) or probability (SVM_BAGGING) with value ranging from 0 to 1 from the output of the machine learning algorithm was normalized to prediction confidence level so that the prediction results from different machine learning models can be compared on a level ground. For each of the 30 protein atom types, the machine learning outputs from the validation sets were sorted into bins of interval 0.1. The prediction confidence level for each of the bins was calculated as the fraction of the true positives over the total number of predictions in the bin. In the end, lookup-tables for output-confidence relationships were constructed; the machine learning outputs can be converted to prediction confidence levels with these lookup tables. Figure S3 shows the relationships between machine learning outputs and the prediction confidence levels for each of the trained machine learning models.

### Five-fold Cross Validation and Independent Test

Five-fold cross validation was performed for each of the 30 protein atom types in the S432 dataset. Each dataset was randomly divided into 5 equal portions with similar distributions of positive and negative cases. One portion of the dataset was selected as test set, another one portion as validation set, and the rest as training set. The training set was used to train the models, and the validation set was used to optimize the prediction parameters so as to achieve the best predictive capability without over-fitting. The optimized models were then benchmarked by the test set. The process took turns to benchmark prediction accuracy on the 5 non-overlapping test sets with the predictors optimized with the corresponding training and validation set. The accuracy benchmarks were the averaged results from the 5-fold cross validation.

For each of the predictors, an optimal threshold for the output activity value was determined with the validation set. Positive predictions have the output activity values greater than or equal to the threshold; the negative predictions have the output activity values smaller than the threshold. With these thresholds, the TP, TN, FP, and FN in Equations (6)∼(11) were determined and the accuracy benchmarks were calculated. The thresholds for the predictors of all 30 atom types were determined to optimize the MCC for the predictions with the validation set.

Five predictors for each protein atom type were optimized after performing the 5-fold cross validation on the S432 dataset. The predictors which yielded the best testing performance were assessed in the independent test with S142, S17a, and S58 dataset.

### Prediction of Patches of Atoms as Protein-protein Binding Sites

A protein-protein binding site was predicted by a cluster of surface atoms predicted as positive cases with high prediction confidence level. Protein surface atoms in PPI sites with prediction confidence level greater than 60% were used as cluster centers to include neighboring surface atoms within radius of 11 Å. Within each of the surface patches, all the surface atoms with the confidence level for positive prediction greater than 20% were included in the tentative patch of atoms as a PPI site. If the pairwise distance of any two seeds was within 10 Å, the two corresponding patches were merged as one patch. The parameters were optimized for residue-based prediction accuracy with the validation set.

### Residue-based Predictions for the PPI Sites

To facilitate comparison of this work with previous methods predicting binding sites at the residue level, a heuristic procedure was used to transform the atom-based binding site predictions as described in the previous paragraph into binding site predictions at the residue level: only the residues with more than 30% of the surface atoms (*SASA_u_*>0) included in the atom-based binding patch were considered as positive residues of the residue-based patch. Similarly, actual PPI sites at the residue level were defined by patches of positive residues, each of which has more than 30% of the surface atoms (*SASA_u_*>0 in the uncomplexed structure) on the residue defined as PPI atoms (*dSASA*>0, as shown in Equation (4)). This definition enabled the comparison of prediction results with actual binding sites at the residue level. The percentage parameter was optimized for residue-based prediction accuracy with the validation set.

### Computational Efficiency for Predicting PPI Sites in a Typical Protein

The building of PDMs for a typical protein of 200 residues with Intel Xeon X5650 (2.67GHz) CPU is around 50 minutes with single thread and around 23 minutes with two threads. The following procedures for generating input attributes and for predicting with machine learning models take less than 20 seconds.

### Mann-Whitney U-test

Mann-Whitney U-test is a non-parametric statistical method to test whether two groups of numerical values come from identical continuous distributions of equal medians – increasing p-value indicates decreasing difference of the two distributions and p-value of 1 indicates that the two distributions are statistically indistinguishable. The Mann-Whitney U-tests were carried out with the statistic tool *ranksum* in MATLAB (http://www.mathworks.com/help/toolbox/stats/ranksum.html).

### Web Site

Predictions can be submitted to the webserver http://ismblab.genomics.sinica.edu.tw/. All the benchmark results can also be accessed in interactive graphic presentations from the same web address above.

## Supporting Information

Figure S1 **Probability density maps and encoded features of human vascular endothelial growth factor A (VEGF).** Structure of VEGF is extracted from PDB ID 2FJG chain V and W. Number 1 to 31 in each cell of the table corresponds to each of the interacting atom types defined in Table 1 of the main text. The PDMs are shown in contours colored according to the interacting atom type: cyan for nitrogen, black for carbon, and magenta for oxygen. The contour level is set to 0.0005. Color spectrum of protein atoms in each cell are based on the corresponding *a_i,j_* values (Equation (3) in the main text). Solvent inaccessible atoms are colored in gray. Interactive 3-D graphic presentation of the PDMs can be viewed from the web server http://ismblab.genomics.sinica.edu.tw/> gallery.
(DOCX)

Figure S2 ***M_min,j_*** **(in square symbols) and** ***M_max,j_*** **(in diamond symbols) against the 32 attribute types.** The maximum and minimum *A_i,j_* values were derived from each protein in S432 and the medians of the maximum (*M_max,j_ j* = 1∼32, shown in diamond symbols) and the minimum (*M_min,j_ j* = 1∼32, shown in square symbols) are plotted against the attribute index. These values were used for normalization of *A_i,j_* (Equation (3) in the main text).
(DOCX)

Figure S3 **Lookup charts converting output activity (probability) from the corresponding machine learning predictor to prediction confidence level.** For each of the 30 protein atom types, the machine learning outputs from the validation sets were sorted into bins of interval 0.1. The confidence level of each of the bins was calculated as the fraction of true positive over the total number of predictions in the bin. The panels (a) and (b) are derived from ANN_BAGGING and SVM_BAGGING predictions respectively. In each of the panel, two sets of curves are shown; one set for the prediction confidence level described as above (i.e., the positive prediction confidence); the other set for the negative prediction confidence. The sum of the positive prediction confidence level and the negative prediction confidence level equals to one.
(DOCX)

Table S1 **A filter system used to eliminate non-interacting atomic pairs based on the work by McConkey et al. with modifications.** During the construction of the PDMs, only the atom pairs with the matrix value less than −0.1 were included in the probability density maps. The atom pairs for which the matrix value colored in red were not included for PDM constructions.
(DOCX)

Table S2 **Five-fold cross validation of ANN_BAGGING prediction accuracy benchmarks on the S432 dataset.** The dataset, the 5-fold cross validation, and the benchmark measurements have been described in the main text. Matthews correlation coefficient (MCC), F-score(Fsc), Accuracy(Acc), Precision(Pre), Sensitivity(Sen) and Specificity(Spe) are shown in Equations (6)∼(11) in the main text. TP, FP, TN, and FN are true positive, false positive, true negative, and false negative respectively. The ratio of the number of predicted positive atoms against actual number of binding atoms for each protein is also listed. C1∼C4 represent PPI sites in each of the test proteins; different protein has different number of PPI sites. In these columns, the number of the predicted true positive atoms is shown over the actual number of atoms involving in the PPI site. Interactive examination of the prediction results for each of the proteins in the S432 dataset can be accessed from the web server: http://ismblab.genomics.sinica.edu.tw/> benchmark >protein-protein.
(DOCX)

Table S3 **Five-fold cross validation of SVM_BAGGING prediction accuracy benchmarks on the S432 dataset.** The dataset, the 5-fold cross validation, and the benchmark measurements have been described in the main text. Matthews correlation coefficient (MCC), F-score(Fsc), Accuracy(Acc), Precision(Pre), Sensitivity(Sen) and Specificity(Spe) are shown in Equations (6)∼(11) in the main text. TP, FP, TN, and FN are true positive, false positive, true negative, and false negative respectively. The ratio of the number of predicted positive atoms against actual number of binding atoms for each protein is also listed. C1∼C4 represent PPI sites in each of the test proteins; different protein has different number of PPI sites. In these columns, the number of the predicted true positive atoms is shown over the actual number of atoms involving in the PPI site. Interactive examination of the prediction results for each of the proteins in the S432 dataset can be accessed from the web server: http://ismblab.genomics.sinica.edu.tw/> benchmark >protein-protein.
(DOCX)

Table S4 **Independent test of ANN_BAGGING prediction accuracy benchmarks on the S142 dataset.** The dataset and the benchmark measurements have been described in the main text. Matthews correlation coefficient (MCC), F-score(Fsc), Accuracy(Acc), Precision(Pre), Sensitivity(Sen) and Specificity(Spe) are shown in Equations (6)∼(11) in the main text. TP, FP, TN, and FN are true positive, false positive, true negative, and false negative respectively. The ratio of the number of predicted positive atoms against actual number of binding atoms for each protein is also listed. C1∼C2 represent PPI sites in each of the test proteins. In these columns, the number of the predicted true positive atoms is shown over the actual number of atoms involving in the PPI site. In the annotation column, complex type (homo or hetero-oligomer) and the secondary structure element (SSE) in the PPI sites are listed for each protein. Interactive examination of the prediction results for each of the proteins in the S142 dataset can be accessed from the web server: http://ismblab.genomics.sinica.edu.tw/> benchmark >protein-protein.
(DOCX)

Table S5 **Independent test of ANN_BAGGING prediction accuracy benchmarks on the S17a dataset.** The dataset and the benchmark measurements have been described in the main text. Matthews correlation coefficient (MCC), F-score(Fsc), Accuracy(Acc), Precision(Pre), Sensitivity(Sen) and Specificity(Spe) are shown in Equations (6)∼(11) in the main text. TP, FP, TN, and FN are true positive, false positive, true negative, and false negative respectively. The ratio of the number of predicted positive atoms against actual number of binding atoms for each protein is also listed. C1∼C2 represent PPI sites in each of the test proteins. In these columns, the number of the predicted true positive atoms is shown over the actual number of atoms involving in the PPI site. Interactive examination of the prediction results for each of the proteins in the S17a dataset can be accessed from the web server: http://ismblab.genomics.sinica.edu.tw/> benchmark >protein-protein.
(DOCX)

Table S6 **Independent test of ANN_BAGGING prediction accuracy benchmarks on the S58 dataset.** The dataset and the benchmark measurements have been described in the main text. Matthews correlation coefficient (MCC), F-score(Fsc), Accuracy(Acc), Precision(Pre), Sensitivity(Sen) and Specificity(Spe) are shown in Equations (6)∼(11) in the main text. TP, FP, TN, and FN are true positive, false positive, true negative, and false negative respectively. The ratio of the number of predicted positive atoms against actual number of binding atoms for each protein is also listed. C1∼C2 represent PPI sites in each of the test proteins. In these columns, the number of the predicted true positive atoms is shown over the actual number of atoms involving in the PPI site. Interactive examination of the prediction results for each of the proteins in the S58 dataset can be accessed from the web server: http://ismblab.genomics.sinica.edu.tw/> benchmark >protein-protein.
(DOCX)

Text S1 **Supplemental methods.**
(DOCX)
