# Supplementary material for: Protein-Protein Interaction Site Predictions with Three-Dimensional Probability Distributions of Interacting Atoms on Protein Surfaces
Source: PLoS One. 2012 Jun 6;7(6):e37706. doi: 10.1371/journal.pone.0037706 (PMC3368894; doi:10.1371/journal.pone.0037706)
Supplement: Figure S1 — Probability density maps and encoded features of human vascular endothelial growth factor A (VEGF). Structure of VEGF is extracted from PDB ID 2FJG chain V and W. Number 1 to 31 in each cell of the table corresponds to each of the interacting atom types defined in Table 1 of the main text. The PDMs are shown in contours colored according to the interacting atom type: cyan for nitrogen, black for carbon, and magenta for oxygen. The contour level is set to 0.0005. Color spectrum of protein atoms in each cell are based on the corresponding ai,j values (Equation (3) in the main text). Solvent inaccessible atoms are colored in gray. Interactive 3-D graphic presentation of the PDMs can be viewed from the web server http://ismblab.genomics.sinica.edu.tw/> gallery. (DOCX) [file pone.0037706.s001.docx]

**Figure S1.**

| 1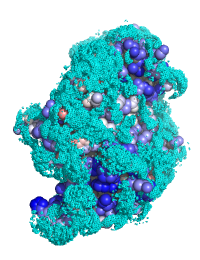  Contour cutoff = 0.0005 | 2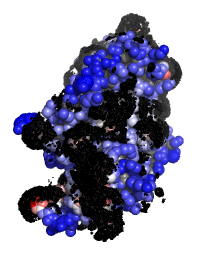 Contour cutoff = 0.0005 | 3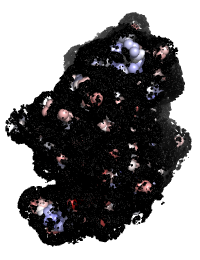 Contour cutoff = 0.0005 |
| --- | --- | --- |
| 4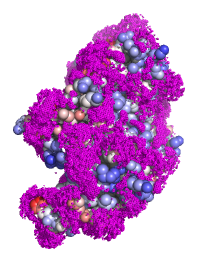 Contour cutoff = 0.0005 | 5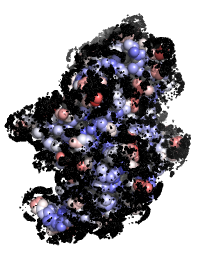 Contour cutoff = 0.0005 | 6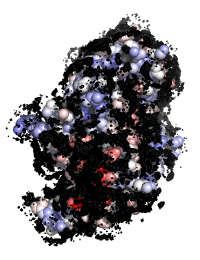 Contour cutoff = 0.0005 |
| 7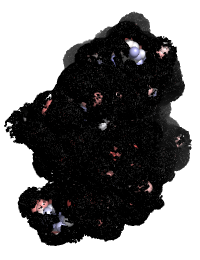  Contour cutoff = 0.0005 | 8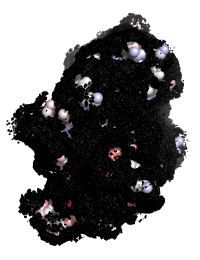  Contour cutoff = 0.0005 | 9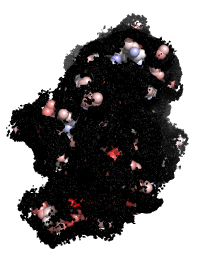  Contour cutoff = 0.0005 |

| 10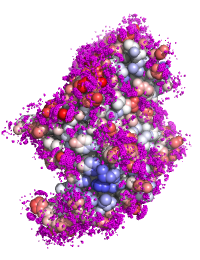 Contour cutoff = 0.0005 | 11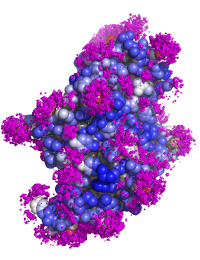 Contour cutoff = 0.0005 | 12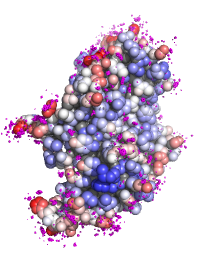 Contour cutoff = 0.0005 |
| --- | --- | --- |
| 13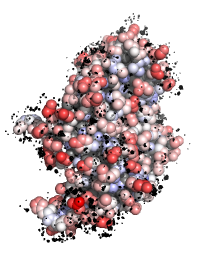 Contour cutoff = 0.0005 | 14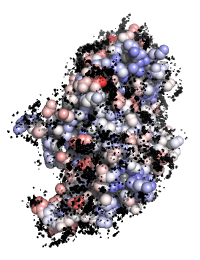 Contour cutoff = 0.0005 | 15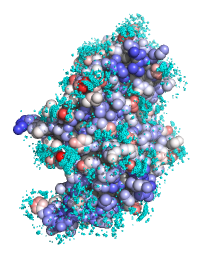 Contour cutoff = 0.0005 |
| 16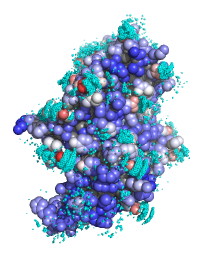 Contour cutoff = 0.0005 | 17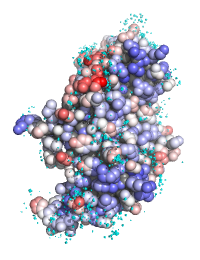 Contour cutoff = 0.0005 | 18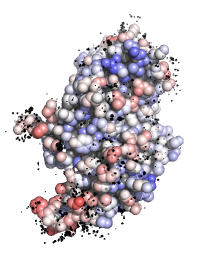 Contour cutoff = 0.0005 |

| 19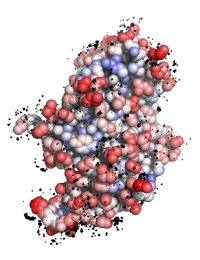 Contour cutoff = 0.0005 | 20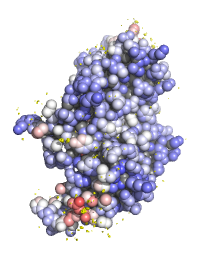 Contour cutoff = 0.0005 | 21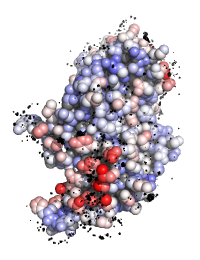 Contour cutoff = 0.0005 |
| --- | --- | --- |
| 22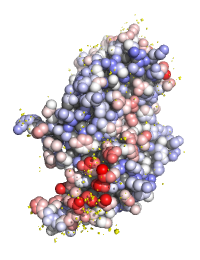 Contour cutoff = 0.0005 | 23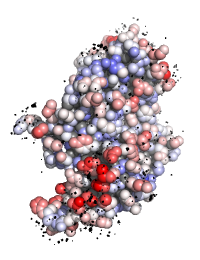 Contour cutoff = 0.0005 | 24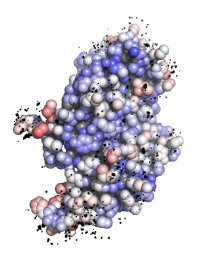 Contour cutoff = 0.0005 |
| 25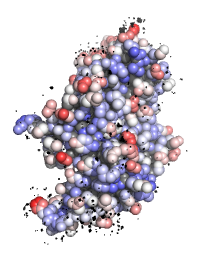 Contour cutoff = 0.0005 | 26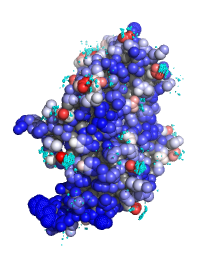 Contour cutoff = 0.0005 | 27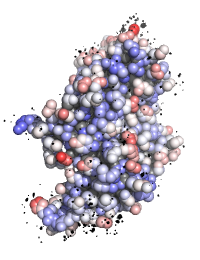 Contour cutoff = 0.0005 |

| 28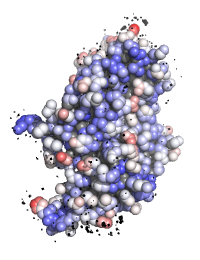 Contour cutoff = 0.0005 | 29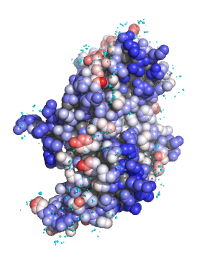 Contour cutoff = 0.0005 | 30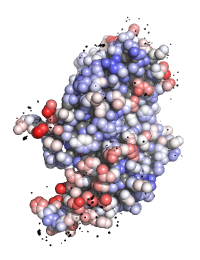 Contour cutoff = 0.0005 |
| --- | --- | --- |
| 31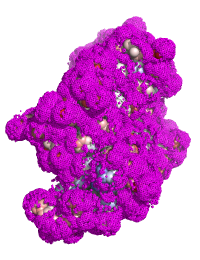 Contour cutoff = 0.0005 |  |  |

Color code of *a_i,j_* for surface atom

0
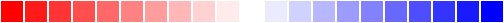
 1

**Figure S1.** Probability density maps and encoded features of human vascular endothelial growth factor A (VEGF). Structure of VEGF is extracted from PDB ID 2FJG chain V and W. Number 1 to 31 in each cell of the table corresponds to each of the interacting atom types defined in Table 1 of the main text. The PDMs are shown in contours colored according to the interacting atom type: cyan for nitrogen, black for carbon, and magenta for oxygen. The contour level is set to 0.0005. Color spectrum of protein atoms in each cell are based on the corresponding *a_i,j_* values (Equation (3) in the main text). Solvent inaccessible atoms are colored in gray. Interactive 3-D graphic presentation of the PDMs can be viewed from the web server <http://ismblab.genomics.sinica.edu.tw/> > gallery.
