# Supplementary material for: Protein-Protein Interaction Site Predictions with Three-Dimensional Probability Distributions of Interacting Atoms on Protein Surfaces
Source: PLoS One. 2012 Jun 6;7(6):e37706. doi: 10.1371/journal.pone.0037706 (PMC3368894; doi:10.1371/journal.pone.0037706)
Supplement: Figure S2 — Mmin,j (in square symbols) and Mmax,j (in diamond symbols) against the 32 attribute types. The maximum and minimum Ai,j values were derived from each protein in S432 and the medians of the maximum (Mmax,j j = 1∼32, shown in diamond symbols) and the minimum (Mmin,j j = 1∼32, shown in square symbols) are plotted against the attribute index. These values were used for normalization of Ai,j (Equation (3) in the main text). (DOCX) [file pone.0037706.s002.docx]

**Figure S2.**

**Figure S2.** *M_min,j_* (in square symbols) and *M_max,j_* (in diamond symbols) against the 32 attribute types. The maximum and minimum *A_i,j_* values were derived from each protein in S432 and the medians of the maximum (*M_max,j_* *j*=1~32, shown in diamond symbols) and the minimum (*M_min,j_* *j*=1~32, shown in square symbols) are plotted against the attribute index. These values were used for normalization of *A_i,j_* (Equation (3) in the main text).
