# Supplementary material for: Protein-Protein Interaction Site Predictions with Three-Dimensional Probability Distributions of Interacting Atoms on Protein Surfaces
Source: PLoS One. 2012 Jun 6;7(6):e37706. doi: 10.1371/journal.pone.0037706 (PMC3368894; doi:10.1371/journal.pone.0037706)
Supplement: Figure S3 — Lookup charts converting output activity (probability) from the corresponding machine learning predictor to prediction confidence level. For each of the 30 protein atom types, the machine learning outputs from the validation sets were sorted into bins of interval 0.1. The confidence level of each of the bins was calculated as the fraction of true positive over the total number of predictions in the bin. The panels (a) and (b) are derived from ANN_BAGGING and SVM_BAGGING predictions respectively. In each of the panel, two sets of curves are shown; one set for the prediction confidence level described as above (i.e., the positive prediction confidence); the other set for the negative prediction confidence. The sum of the positive prediction confidence level and the negative prediction confidence level equals to one. (DOCX) [file pone.0037706.s003.docx]

**Figure S3.**

(a)


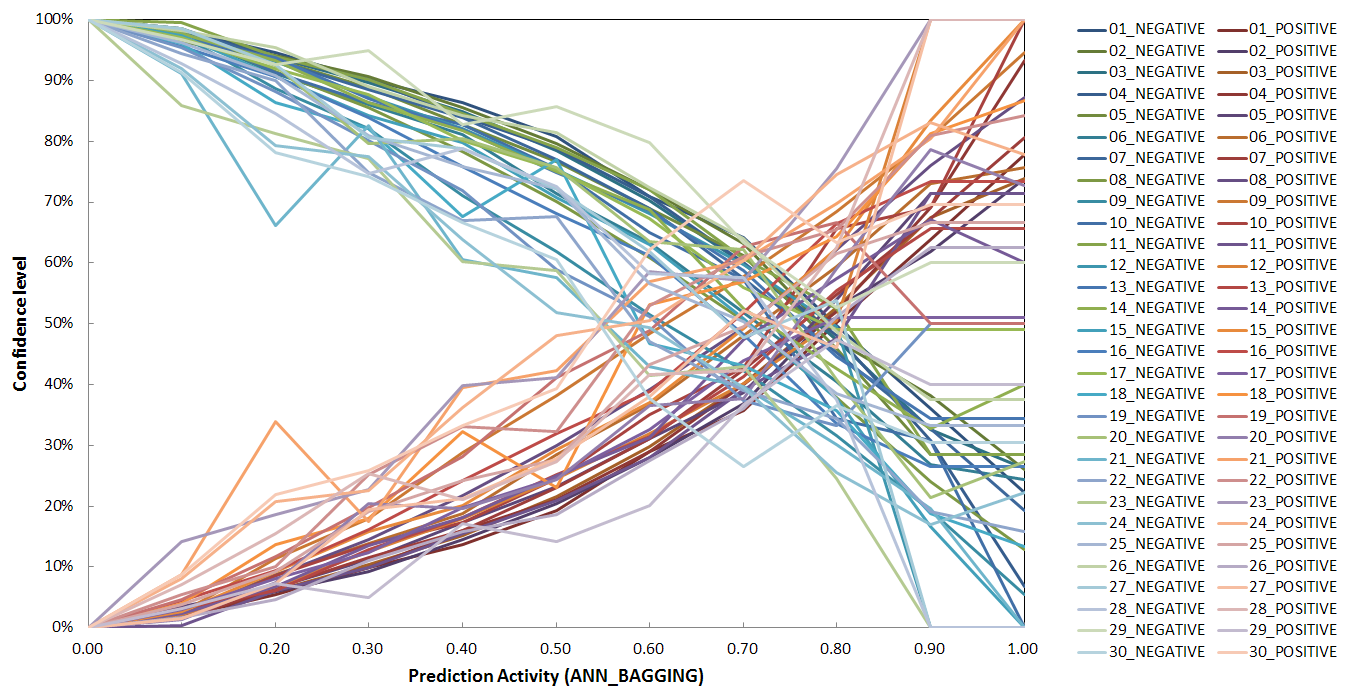


(b)


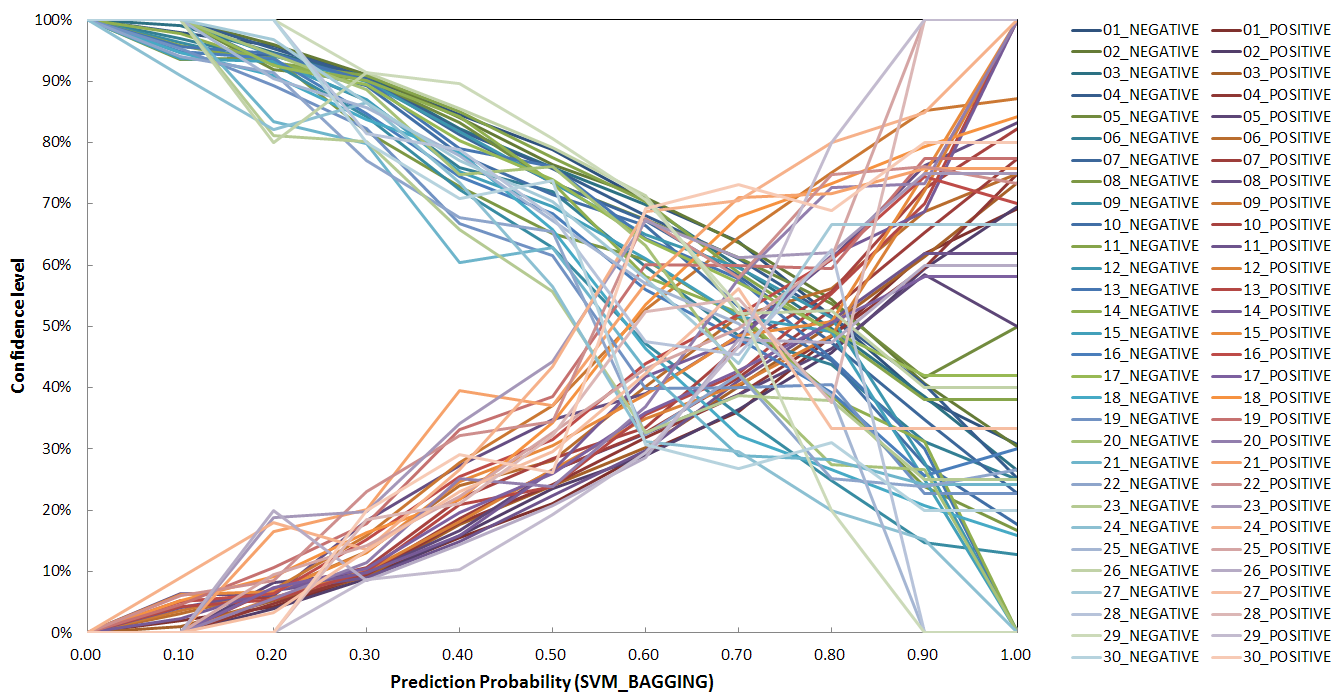


**Figure S3.** Lookup charts converting output activity (probability) from the corresponding machine learning predictor to prediction confidence level. For each of the 30 protein atom types, the machine learning outputs from the validation sets were sorted into bins of interval 0.1. The confidence level of each of the bins was calculated as the fraction of true positive over the total number of predictions in the bin. The panels (a) and (b) are derived from ANN_BAGGING and SVM_BAGGING predictions respectively. In each of the panel, two sets of curves are shown; one set for the prediction confidence level described as above (i.e., the positive prediction confidence); the other set for the negative prediction confidence. The sum of the positive prediction confidence level and the negative prediction confidence level equals to one.
