# Supplementary material for: Protein-Protein Interaction Site Predictions with Three-Dimensional Probability Distributions of Interacting Atoms on Protein Surfaces
Source: PLoS One. 2012 Jun 6;7(6):e37706. doi: 10.1371/journal.pone.0037706 (PMC3368894; doi:10.1371/journal.pone.0037706)
Supplement: Table S1 — A filter system used to eliminate non-interacting atomic pairs based on the work by McConkey et al. with modifications. During the construction of the PDMs, only the atom pairs with the matrix value less than −0.1 were included in the probability density maps. The atom pairs for which the matrix value colored in red were not included for PDM constructions. (DOCX) [file pone.0037706.s004.docx]

**Table S1**. **A filter system used to eliminate non-interacting atomic pairs.**


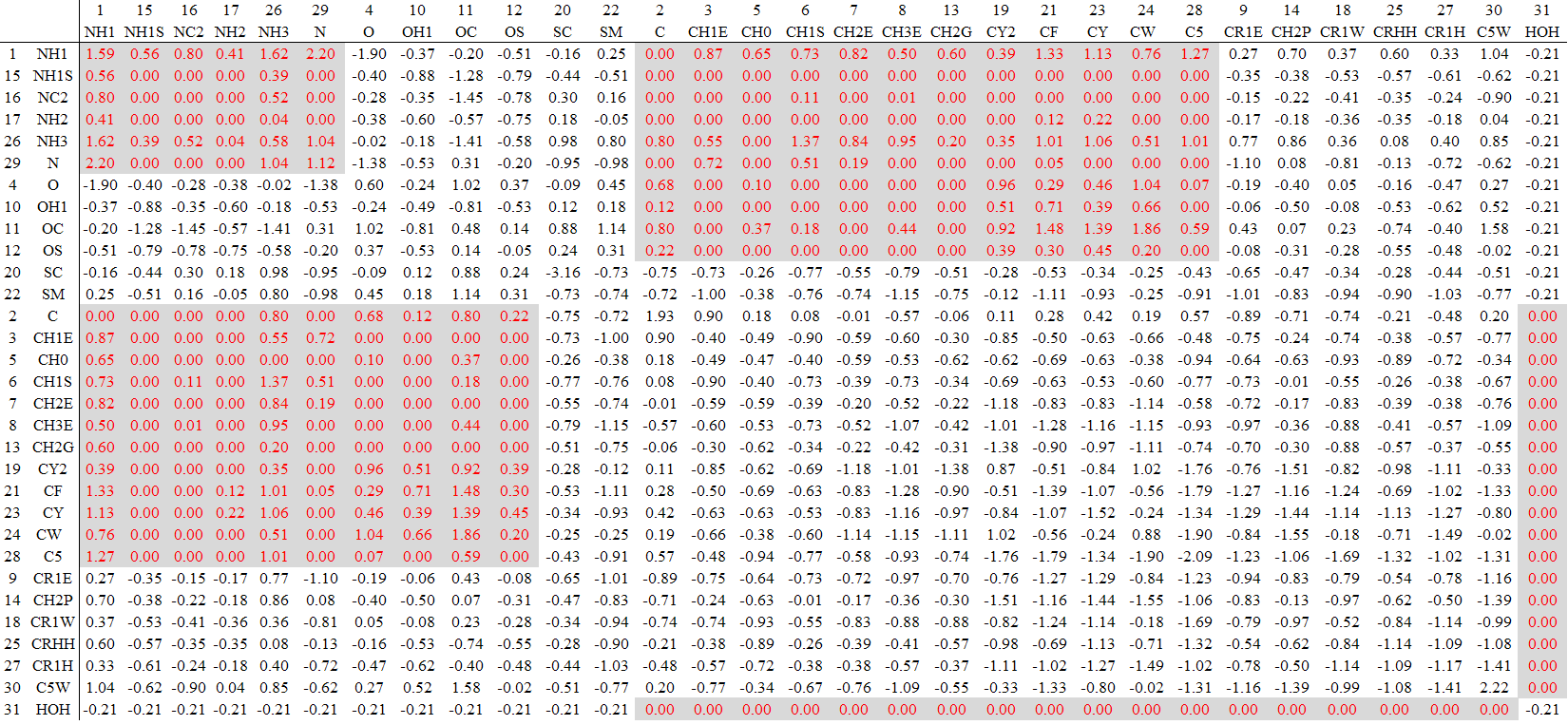


**Table S1:** A filter system used to eliminate non-interacting atomic pairs based on the work by McConkey et al. [[12](#_ENREF_12)] with modifications. During the construction of the PDMs, only the atom pairs with the matrix value less than -0.1 were included in the probability density maps. The atom pairs for which the matrix value colored in red were not included for PDM constructions.
