# Supplementary material for: Protein-Protein Interaction Site Predictions with Three-Dimensional Probability Distributions of Interacting Atoms on Protein Surfaces
Source: PLoS One. 2012 Jun 6;7(6):e37706. doi: 10.1371/journal.pone.0037706 (PMC3368894; doi:10.1371/journal.pone.0037706)
Supplement: Table S2 — Five-fold cross validation of ANN_BAGGING prediction accuracy benchmarks on the S432 dataset. The dataset, the 5-fold cross validation, and the benchmark measurements have been described in the main text. Matthews correlation coefficient (MCC), F-score(Fsc), Accuracy(Acc), Precision(Pre), Sensitivity(Sen) and Specificity(Spe) are shown in Equations (6)∼(11) in the main text. TP, FP, TN, and FN are true positive, false positive, true negative, and false negative respectively. The ratio of the number of predicted positive atoms against actual number of binding atoms for each protein is also listed. C1∼C4 represent PPI sites in each of the test proteins; different protein has different number of PPI sites. In these columns, the number of the predicted true positive atoms is shown over the actual number of atoms involving in the PPI site. Interactive examination of the prediction results for each of the proteins in the S432 dataset can be accessed from the web server: http://ismblab.genomics.sinica.edu.tw/> benchmark >protein-protein. (DOCX) [file pone.0037706.s005.docx]

**Table S2. Residue-based cross validation benchmarks of ANN_BAGGING with S432 dataset.**

|  | Residue Level Benchmark | | | | | | | | | | Predict positive atoms / Actual binding atoms | | | | |
| --- | --- | --- | --- | --- | --- | --- | --- | --- | --- | --- | --- | --- | --- | --- | --- |
| PDBID | Acc | Pre | Sen | Spe | MCC | Fsc | TP | TN | FP | FN | All | C1 | C2 | C3 | C4 |
| 1byfA | 0.918 | 0.818 | 0.783 | 0.954 | 0.749 | 0.8 | 18 | 83 | 4 | 5 | 71/91 | 71/91 |  |  |  |
| 1jtdB | 0.944 | 0.781 | 0.781 | 0.968 | 0.749 | 0.781 | 25 | 213 | 7 | 7 | 90/124 | 90/124 |  |  |  |
| 1n13B | 0.874 | 0.838 | 0.966 | 0.75 | 0.748 | 0.898 | 57 | 33 | 11 | 2 | 235/251 | 235/251 |  |  |  |
| 1h1yA | 0.934 | 0.778 | 0.75 | 0.964 | 0.725 | 0.764 | 21 | 162 | 6 | 7 | 67/100 | 67/100 |  |  |  |
| 1bykA | 0.914 | 0.689 | 0.861 | 0.925 | 0.721 | 0.765 | 31 | 172 | 14 | 5 | 111/145 | 111/145 |  |  |  |
| 1hynP | 0.885 | 0.649 | 0.926 | 0.874 | 0.709 | 0.763 | 50 | 188 | 27 | 4 | 215/244 | 215/244 |  |  |  |
| 1j79A | 0.949 | 0.759 | 0.71 | 0.975 | 0.706 | 0.733 | 22 | 278 | 7 | 9 | 74/118 | 74/118 |  |  |  |
| 12asA | 0.903 | 0.646 | 0.875 | 0.908 | 0.697 | 0.743 | 42 | 228 | 23 | 6 | 151/186 | 151/186 |  |  |  |
| 1k8kD | 0.873 | 0.829 | 0.725 | 0.936 | 0.688 | 0.773 | 58 | 175 | 12 | 22 | 252/364 | 252/364 |  |  |  |
| 1hn2A | 0.852 | 0.738 | 0.865 | 0.845 | 0.686 | 0.796 | 45 | 87 | 16 | 7 | 194/244 | 194/244 |  |  |  |
| 1k8kE | 0.921 | 0.593 | 0.889 | 0.925 | 0.686 | 0.711 | 16 | 136 | 11 | 2 | 83/94 | 83/94 |  |  |  |
| 1ig0A | 0.916 | 0.681 | 0.78 | 0.939 | 0.68 | 0.727 | 32 | 229 | 15 | 9 | 115/161 | 115/161 |  |  |  |
| 1b16A | 0.865 | 0.7 | 0.831 | 0.876 | 0.671 | 0.76 | 49 | 149 | 21 | 10 | 199/245 | 199/245 |  |  |  |
| 1k28A | 0.829 | 0.919 | 0.777 | 0.902 | 0.669 | 0.842 | 248 | 202 | 22 | 71 | 1383/1759 | 1383/1759 |  |  |  |
| 1cydA | 0.847 | 0.844 | 0.692 | 0.931 | 0.656 | 0.761 | 54 | 134 | 10 | 24 | 222/341 | 222/341 |  |  |  |
| 1mg2A | 0.86 | 0.785 | 0.723 | 0.917 | 0.656 | 0.753 | 73 | 221 | 20 | 28 | 280/422 | 280/422 |  |  |  |
| 1at3A | 0.884 | 0.568 | 0.893 | 0.882 | 0.651 | 0.694 | 25 | 142 | 19 | 3 | 103/118 | 103/118 |  |  |  |
| 1jeqB | 0.812 | 0.718 | 0.899 | 0.751 | 0.64 | 0.798 | 186 | 220 | 73 | 21 | 832/987 | 810/932 | 22/55 |  |  |
| 1h59B | 0.81 | 0.714 | 0.882 | 0.76 | 0.631 | 0.789 | 15 | 19 | 6 | 2 | 59/72 | 59/72 |  |  |  |
| 1jmvA | 0.805 | 0.529 | 1 | 0.75 | 0.63 | 0.692 | 27 | 72 | 24 | 0 | 109/120 | 109/120 |  |  |  |
| 1bouA | 0.808 | 0.75 | 0.879 | 0.746 | 0.626 | 0.81 | 51 | 50 | 17 | 7 | 205/245 | 205/245 |  |  |  |
| 1l2wA | 0.812 | 0.794 | 0.871 | 0.745 | 0.624 | 0.831 | 54 | 41 | 14 | 8 | 210/263 | 210/263 |  |  |  |
| 1rveA | 0.898 | 0.684 | 0.684 | 0.939 | 0.624 | 0.684 | 26 | 186 | 12 | 12 | 91/160 | 91/146 | 0/14 |  |  |
| 1bazA | 0.816 | 0.794 | 0.931 | 0.65 | 0.62 | 0.857 | 27 | 13 | 7 | 2 | 144/161 | 144/161 |  |  |  |
| 1lm8B | 0.78 | 0.551 | 1 | 0.699 | 0.62 | 0.711 | 27 | 51 | 22 | 0 | 112/119 | 112/119 |  |  |  |
| 1gcqC | 0.781 | 0.683 | 0.966 | 0.629 | 0.616 | 0.8 | 28 | 22 | 13 | 1 | 92/111 | 92/111 |  |  |  |
| 1aihA | 0.813 | 0.656 | 0.857 | 0.792 | 0.613 | 0.743 | 42 | 84 | 22 | 7 | 156/208 | 156/208 |  |  |  |
| 1euaA | 0.894 | 0.769 | 0.588 | 0.961 | 0.613 | 0.667 | 20 | 149 | 6 | 14 | 68/116 | 68/116 |  |  |  |
| 1jjuB | 0.889 | 0.655 | 0.692 | 0.928 | 0.606 | 0.673 | 36 | 244 | 19 | 16 | 132/202 | 132/202 |  |  |  |
| 1l1oA | 0.804 | 0.63 | 0.879 | 0.77 | 0.605 | 0.734 | 29 | 57 | 17 | 4 | 121/151 | 121/151 |  |  |  |
| 1avgI | 0.904 | 0.846 | 0.5 | 0.982 | 0.604 | 0.629 | 11 | 111 | 2 | 11 | 39/87 | 39/87 |  |  |  |
| 1ffgB | 0.841 | 0.667 | 0.75 | 0.872 | 0.6 | 0.706 | 12 | 41 | 6 | 4 | 53/65 | 53/65 |  |  |  |
| 1l6xB | 0.794 | 0.737 | 0.875 | 0.722 | 0.6 | 0.8 | 14 | 13 | 5 | 2 | 63/77 | 63/77 |  |  |  |
| 1dcuA | 0.838 | 0.753 | 0.663 | 0.91 | 0.596 | 0.705 | 55 | 183 | 18 | 28 | 211/342 | 211/342 |  |  |  |
| 1fcdC | 0.844 | 0.574 | 0.818 | 0.851 | 0.592 | 0.675 | 27 | 114 | 20 | 6 | 103/138 | 103/138 |  |  |  |
| 1fm0E | 0.799 | 0.83 | 0.672 | 0.895 | 0.589 | 0.743 | 39 | 68 | 8 | 19 | 157/241 | 157/241 |  |  |  |
| 1nvtA | 0.838 | 0.48 | 0.923 | 0.824 | 0.588 | 0.632 | 36 | 182 | 39 | 3 | 122/146 | 122/146 |  |  |  |
| 1g99A | 0.855 | 0.624 | 0.726 | 0.888 | 0.581 | 0.671 | 53 | 253 | 32 | 20 | 214/315 | 214/315 |  |  |  |
| 1j7dA | 0.822 | 0.425 | 1 | 0.795 | 0.581 | 0.596 | 17 | 89 | 23 | 0 | 67/79 | 67/79 |  |  |  |
| 1id1A | 0.796 | 0.614 | 0.854 | 0.771 | 0.58 | 0.714 | 35 | 74 | 22 | 6 | 130/162 | 130/162 |  |  |  |
| 1eaiC | 0.8 | 0.833 | 0.625 | 0.917 | 0.579 | 0.714 | 15 | 33 | 3 | 9 | 59/93 | 59/93 |  |  |  |
| 1ixsA | 0.795 | 0.611 | 0.846 | 0.774 | 0.576 | 0.71 | 11 | 24 | 7 | 2 | 52/66 | 52/66 |  |  |  |
| 1b8gA | 0.87 | 0.585 | 0.727 | 0.899 | 0.575 | 0.649 | 48 | 301 | 34 | 18 | 200/278 | 200/278 |  |  |  |
| 1ik9A | 0.755 | 0.565 | 0.953 | 0.664 | 0.574 | 0.709 | 61 | 93 | 47 | 3 | 274/312 | 274/312 |  |  |  |
| 1ax4A | 0.852 | 0.686 | 0.641 | 0.914 | 0.569 | 0.663 | 59 | 286 | 27 | 33 | 253/416 | 253/416 |  |  |  |
| 1kpsB | 0.786 | 0.464 | 0.963 | 0.746 | 0.567 | 0.627 | 26 | 88 | 30 | 1 | 113/134 | 113/134 |  |  |  |
| 1keyA | 0.789 | 0.731 | 0.74 | 0.821 | 0.56 | 0.735 | 57 | 96 | 21 | 20 | 225/332 | 225/332 |  |  |  |
| 1ituA | 0.894 | 0.743 | 0.51 | 0.967 | 0.559 | 0.605 | 26 | 261 | 9 | 25 | 90/213 | 90/213 |  |  |  |
| 3fivA | 0.77 | 0.691 | 0.864 | 0.696 | 0.559 | 0.768 | 38 | 39 | 17 | 6 | 154/185 | 154/185 |  |  |  |
| 1b0nB | 0.935 | 0.933 | 1 | 0.333 | 0.558 | 0.966 | 28 | 1 | 2 | 0 | 141/150 | 141/150 |  |  |  |
| 1k8kF | 0.769 | 0.915 | 0.714 | 0.873 | 0.558 | 0.802 | 75 | 48 | 7 | 30 | 336/517 | 336/517 |  |  |  |
| 1lk5A | 0.818 | 0.714 | 0.656 | 0.887 | 0.557 | 0.684 | 40 | 126 | 16 | 21 | 124/235 | 124/235 |  |  |  |
| 1lktA | 0.784 | 0.783 | 0.855 | 0.69 | 0.556 | 0.817 | 47 | 29 | 13 | 8 | 158/213 | 158/213 |  |  |  |
| 1m6pA | 0.857 | 0.667 | 0.621 | 0.919 | 0.554 | 0.643 | 18 | 102 | 9 | 11 | 60/109 | 60/109 |  |  |  |
| 1qq5A | 0.826 | 0.613 | 0.731 | 0.856 | 0.554 | 0.667 | 38 | 143 | 24 | 14 | 155/241 | 155/241 |  |  |  |
| 1apyB | 0.792 | 0.831 | 0.841 | 0.708 | 0.552 | 0.836 | 69 | 34 | 14 | 13 | 295/383 | 295/383 |  |  |  |
| 1go3F | 0.774 | 0.727 | 0.78 | 0.769 | 0.547 | 0.753 | 32 | 40 | 12 | 9 | 134/200 | 134/200 |  |  |  |
| 1i0dA | 0.877 | 0.482 | 0.771 | 0.891 | 0.546 | 0.593 | 27 | 237 | 29 | 8 | 97/152 | 97/152 |  |  |  |
| 1jyoA | 0.77 | 0.841 | 0.736 | 0.815 | 0.545 | 0.785 | 53 | 44 | 10 | 19 | 189/280 | 189/280 |  |  |  |
| 1jeqA | 0.775 | 0.702 | 0.778 | 0.772 | 0.543 | 0.738 | 165 | 237 | 70 | 47 | 741/1006 | 741/949 | 0/57 |  |  |
| 1l9wA | 0.857 | 0.396 | 0.913 | 0.851 | 0.543 | 0.553 | 21 | 183 | 32 | 2 | 82/93 | 82/93 |  |  |  |
| 1e44A | 0.775 | 0.686 | 0.774 | 0.776 | 0.54 | 0.727 | 24 | 38 | 11 | 7 | 106/146 | 106/146 |  |  |  |
| 1h7eA | 0.889 | 0.633 | 0.576 | 0.943 | 0.54 | 0.603 | 19 | 181 | 11 | 14 | 82/152 | 82/152 |  |  |  |
| 1ltsC | 0.927 | 0.947 | 0.973 | 0.5 | 0.539 | 0.96 | 36 | 2 | 2 | 1 | 206/227 | 206/227 |  |  |  |
| 1e2aA | 0.755 | 0.684 | 0.886 | 0.64 | 0.538 | 0.772 | 39 | 32 | 18 | 5 | 161/198 | 161/198 |  |  |  |
| 1k83K | 0.788 | 0.859 | 0.813 | 0.737 | 0.538 | 0.836 | 61 | 28 | 10 | 14 | 259/340 | 259/340 |  |  |  |
| 1axiB | 0.812 | 0.688 | 0.647 | 0.88 | 0.537 | 0.667 | 33 | 110 | 15 | 18 | 131/226 | 131/226 |  |  |  |
| 1g57A | 0.845 | 0.605 | 0.667 | 0.89 | 0.537 | 0.634 | 26 | 138 | 17 | 13 | 89/146 | 89/146 |  |  |  |
| 1gg2B | 0.791 | 0.706 | 0.686 | 0.847 | 0.536 | 0.696 | 72 | 166 | 30 | 33 | 213/365 | 213/365 |  |  |  |
| 1gvnA | 0.738 | 0.95 | 0.655 | 0.923 | 0.535 | 0.776 | 38 | 24 | 2 | 20 | 163/264 | 163/264 |  |  |  |
| 1avqA | 0.816 | 0.422 | 0.9 | 0.802 | 0.531 | 0.574 | 27 | 150 | 37 | 3 | 109/127 | 50/62 | 59/65 |  |  |
| 1m2dA | 0.739 | 0.415 | 1 | 0.68 | 0.531 | 0.586 | 17 | 51 | 24 | 0 | 55/62 | 55/62 |  |  |  |
| 1d9eA | 0.793 | 0.86 | 0.481 | 0.959 | 0.529 | 0.617 | 37 | 139 | 6 | 40 | 170/330 | 170/330 |  |  |  |
| 1udiI | 0.769 | 0.647 | 0.786 | 0.76 | 0.528 | 0.71 | 22 | 38 | 12 | 6 | 73/109 | 73/109 |  |  |  |
| 1k3bA | 0.766 | 0.755 | 0.725 | 0.8 | 0.527 | 0.74 | 37 | 48 | 12 | 14 | 144/219 | 144/219 |  |  |  |
| 1h21A | 0.752 | 0.675 | 0.856 | 0.669 | 0.526 | 0.755 | 83 | 81 | 40 | 14 | 340/445 | 340/445 |  |  |  |
| 1i1rB | 0.717 | 0.462 | 0.973 | 0.635 | 0.522 | 0.626 | 36 | 73 | 42 | 1 | 156/165 | 63/71 | 93/94 |  |  |
| 1e44B | 0.822 | 0.846 | 0.44 | 0.969 | 0.521 | 0.579 | 11 | 63 | 2 | 14 | 58/129 | 58/129 |  |  |  |
| 1eteA | 0.82 | 0.444 | 0.842 | 0.817 | 0.521 | 0.582 | 16 | 89 | 20 | 3 | 71/91 | 71/91 |  |  |  |
| 1e7lA | 0.763 | 0.731 | 0.731 | 0.788 | 0.52 | 0.731 | 49 | 67 | 18 | 18 | 234/336 | 234/336 |  |  |  |
| 1f8mA | 0.752 | 0.856 | 0.566 | 0.916 | 0.52 | 0.682 | 107 | 196 | 18 | 82 | 445/805 | 445/805 |  |  |  |
| 1poiB | 0.802 | 0.639 | 0.676 | 0.851 | 0.519 | 0.657 | 46 | 149 | 26 | 22 | 166/279 | 166/279 |  |  |  |
| 1d4xG | 0.812 | 0.613 | 0.679 | 0.857 | 0.518 | 0.644 | 19 | 72 | 12 | 9 | 72/103 | 72/103 |  |  |  |
| 1gz0A | 0.828 | 0.448 | 0.812 | 0.831 | 0.514 | 0.578 | 26 | 157 | 32 | 6 | 87/131 | 87/131 |  |  |  |
| 1n0wB | 0.788 | 0.826 | 0.864 | 0.636 | 0.513 | 0.844 | 19 | 7 | 4 | 3 | 82/93 | 82/93 |  |  |  |
| 1ei1A | 0.853 | 0.667 | 0.535 | 0.933 | 0.51 | 0.594 | 38 | 264 | 19 | 33 | 139/281 | 117/242 | 22/39 |  |  |
| 1k83H | 0.775 | 0.625 | 0.732 | 0.795 | 0.508 | 0.674 | 30 | 70 | 18 | 11 | 129/201 | 129/201 |  |  |  |
| 1l0oA | 0.773 | 0.727 | 0.64 | 0.854 | 0.508 | 0.681 | 32 | 70 | 12 | 18 | 109/196 | 109/196 |  |  |  |
| 1ireA | 0.733 | 0.881 | 0.547 | 0.924 | 0.507 | 0.675 | 52 | 85 | 7 | 43 | 227/440 | 227/440 |  |  |  |
| 1bjnA | 0.831 | 0.524 | 0.705 | 0.859 | 0.506 | 0.601 | 43 | 237 | 39 | 18 | 131/222 | 131/222 |  |  |  |
| 1dbfA | 0.758 | 0.735 | 0.692 | 0.809 | 0.505 | 0.713 | 36 | 55 | 13 | 16 | 150/212 | 150/212 |  |  |  |
| 1smtA | 0.732 | 0.638 | 0.881 | 0.618 | 0.504 | 0.74 | 37 | 34 | 21 | 5 | 161/200 | 161/200 |  |  |  |
| 1fuxA | 0.859 | 0.5 | 0.682 | 0.888 | 0.503 | 0.577 | 15 | 119 | 15 | 7 | 56/90 | 56/90 |  |  |  |
| 1jkeA | 0.774 | 0.612 | 0.732 | 0.793 | 0.503 | 0.667 | 30 | 73 | 19 | 11 | 119/184 | 119/184 |  |  |  |
| 1isiA | 0.879 | 0.5 | 0.643 | 0.912 | 0.499 | 0.563 | 18 | 186 | 18 | 10 | 91/131 | 74/87 | 17/44 |  |  |
| 1jr8A | 0.706 | 0.5 | 0.933 | 0.611 | 0.499 | 0.651 | 28 | 44 | 28 | 2 | 116/133 | 116/133 |  |  |  |
| 1ed9A | 0.835 | 0.768 | 0.448 | 0.957 | 0.498 | 0.566 | 43 | 290 | 13 | 53 | 150/405 | 150/405 |  |  |  |
| 1fcdA | 0.9 | 0.548 | 0.561 | 0.942 | 0.498 | 0.554 | 23 | 309 | 19 | 18 | 91/178 | 91/178 |  |  |  |
| 1fuiA | 0.848 | 0.584 | 0.596 | 0.904 | 0.496 | 0.59 | 59 | 397 | 42 | 40 | 239/416 | 239/416 |  |  |  |
| 1f34B | 0.741 | 0.532 | 0.846 | 0.698 | 0.495 | 0.653 | 33 | 67 | 29 | 6 | 119/157 | 119/157 |  |  |  |
| 1hq3A | 0.794 | 0.81 | 0.914 | 0.531 | 0.495 | 0.859 | 64 | 17 | 15 | 6 | 278/321 | 278/321 |  |  |  |
| 1j5sA | 0.837 | 0.719 | 0.479 | 0.944 | 0.494 | 0.575 | 46 | 302 | 18 | 50 | 192/434 | 192/434 |  |  |  |
| 1fm0D | 0.714 | 0.5 | 0.909 | 0.636 | 0.493 | 0.645 | 20 | 35 | 20 | 2 | 73/85 | 73/85 |  |  |  |
| 1ep3B | 0.861 | 0.524 | 0.629 | 0.901 | 0.492 | 0.571 | 22 | 183 | 20 | 13 | 92/138 | 92/138 |  |  |  |
| 1luaA | 0.863 | 0.449 | 0.71 | 0.883 | 0.491 | 0.55 | 22 | 204 | 27 | 9 | 82/117 | 82/117 |  |  |  |
| 1tyfA | 0.744 | 0.767 | 0.733 | 0.756 | 0.489 | 0.75 | 66 | 62 | 20 | 24 | 267/422 | 267/422 |  |  |  |
| 1n9rA | 0.733 | 0.676 | 0.862 | 0.613 | 0.488 | 0.758 | 25 | 19 | 12 | 4 | 116/134 | 116/134 |  |  |  |
| 1aonO | 0.742 | 0.717 | 0.809 | 0.674 | 0.487 | 0.76 | 38 | 31 | 15 | 9 | 151/195 | 39/40 | 112/155 |  |  |
| 1b33A | 0.753 | 0.603 | 0.76 | 0.75 | 0.487 | 0.673 | 38 | 75 | 25 | 12 | 155/205 | 155/205 |  |  |  |
| 1c28A | 0.748 | 0.708 | 0.708 | 0.778 | 0.486 | 0.708 | 34 | 49 | 14 | 14 | 137/216 | 137/216 |  |  |  |
| 1tdtA | 0.758 | 0.581 | 0.761 | 0.756 | 0.486 | 0.659 | 54 | 121 | 39 | 17 | 227/312 | 227/312 |  |  |  |
| 1dtwA | 0.747 | 0.743 | 0.66 | 0.817 | 0.485 | 0.699 | 101 | 156 | 35 | 52 | 419/659 | 419/659 |  |  |  |
| 1k8kG | 0.775 | 0.735 | 0.556 | 0.893 | 0.485 | 0.633 | 25 | 75 | 9 | 20 | 121/213 | 121/213 |  |  |  |
| 1jg5A | 0.741 | 0.762 | 0.744 | 0.737 | 0.48 | 0.753 | 32 | 28 | 10 | 11 | 129/192 | 129/192 |  |  |  |
| 1pvuA | 0.729 | 0.485 | 0.865 | 0.682 | 0.48 | 0.621 | 32 | 73 | 34 | 5 | 134/171 | 134/171 |  |  |  |
| 1kwsA | 0.823 | 0.636 | 0.549 | 0.905 | 0.479 | 0.589 | 28 | 153 | 16 | 23 | 109/212 | 109/212 |  |  |  |
| 1a2xA | 0.778 | 0.638 | 0.638 | 0.84 | 0.478 | 0.638 | 30 | 89 | 17 | 17 | 113/193 | 113/193 |  |  |  |
| 1k75A | 0.76 | 0.605 | 0.712 | 0.782 | 0.476 | 0.654 | 89 | 208 | 58 | 36 | 350/539 | 350/539 |  |  |  |
| 1ixmA | 0.694 | 0.456 | 0.929 | 0.612 | 0.475 | 0.612 | 26 | 49 | 31 | 2 | 118/128 | 118/128 |  |  |  |
| 1ld8A | 0.801 | 0.75 | 0.464 | 0.938 | 0.475 | 0.574 | 39 | 195 | 13 | 45 | 178/367 | 178/367 |  |  |  |
| 1ajsA | 0.813 | 0.622 | 0.567 | 0.891 | 0.473 | 0.593 | 51 | 254 | 31 | 39 | 185/345 | 185/345 |  |  |  |
| 1dj0A | 0.827 | 0.474 | 0.692 | 0.853 | 0.472 | 0.562 | 27 | 174 | 30 | 12 | 107/156 | 107/156 |  |  |  |
| 1fo0A | 0.7 | 0.483 | 0.903 | 0.62 | 0.472 | 0.629 | 28 | 49 | 30 | 3 | 107/131 | 107/131 |  |  |  |
| 1f3uB | 0.688 | 0.547 | 0.922 | 0.552 | 0.471 | 0.686 | 47 | 48 | 39 | 4 | 217/245 | 217/245 |  |  |  |
| 1diqC | 0.676 | 0.452 | 0.95 | 0.574 | 0.47 | 0.613 | 19 | 31 | 23 | 1 | 87/97 | 87/97 |  |  |  |
| 1jtgB | 0.786 | 0.5 | 0.727 | 0.802 | 0.469 | 0.593 | 24 | 97 | 24 | 9 | 117/153 | 117/153 |  |  |  |
| 1kb9F | 0.726 | 0.641 | 0.806 | 0.667 | 0.469 | 0.714 | 25 | 28 | 14 | 6 | 95/146 | 95/146 |  |  |  |
| 1a79A | 0.744 | 0.694 | 0.672 | 0.793 | 0.468 | 0.683 | 43 | 73 | 19 | 21 | 185/272 | 185/272 |  |  |  |
| 1adjA | 0.812 | 0.633 | 0.543 | 0.899 | 0.467 | 0.585 | 50 | 257 | 29 | 42 | 202/401 | 202/401 |  |  |  |
| 1dkgA | 0.727 | 0.681 | 0.838 | 0.618 | 0.467 | 0.752 | 62 | 47 | 29 | 12 | 249/309 | 249/309 |  |  |  |
| 1el6A | 0.734 | 0.725 | 0.775 | 0.691 | 0.467 | 0.749 | 79 | 67 | 30 | 23 | 288/417 | 288/417 |  |  |  |
| 1swuA | 0.707 | 0.875 | 0.483 | 0.931 | 0.463 | 0.622 | 28 | 54 | 4 | 30 | 104/225 | 104/225 |  |  |  |
| 1devB | 0.8 | 0.784 | 1 | 0.273 | 0.462 | 0.879 | 29 | 3 | 8 | 0 | 116/126 | 116/126 |  |  |  |
| 1e3vA | 0.714 | 0.464 | 0.867 | 0.663 | 0.461 | 0.605 | 26 | 59 | 30 | 4 | 82/118 | 82/118 |  |  |  |
| 1lh0A | 0.785 | 0.483 | 0.725 | 0.8 | 0.459 | 0.58 | 29 | 124 | 31 | 11 | 104/167 | 104/167 |  |  |  |
| 1g8mA | 0.782 | 0.535 | 0.68 | 0.814 | 0.458 | 0.599 | 85 | 323 | 74 | 40 | 365/573 | 365/573 |  |  |  |
| 1g0sA | 0.723 | 0.66 | 0.791 | 0.667 | 0.457 | 0.72 | 68 | 70 | 35 | 18 | 299/397 | 299/397 |  |  |  |
| 1tbrR | 0.723 | 0.627 | 0.78 | 0.683 | 0.456 | 0.696 | 32 | 41 | 19 | 9 | 125/173 | 125/173 |  |  |  |
| 1g64A | 0.708 | 0.566 | 0.839 | 0.633 | 0.455 | 0.676 | 47 | 62 | 36 | 9 | 181/237 | 181/237 |  |  |  |
| 1evxA | 0.724 | 0.6 | 0.776 | 0.694 | 0.454 | 0.677 | 45 | 68 | 30 | 13 | 206/269 | 206/269 |  |  |  |
| 1ynjA | 0.733 | 0.693 | 0.678 | 0.775 | 0.454 | 0.685 | 61 | 93 | 27 | 29 | 247/390 | 247/390 |  |  |  |
| 1n0wA | 0.702 | 0.341 | 0.967 | 0.652 | 0.453 | 0.504 | 29 | 105 | 56 | 1 | 118/128 | 118/128 |  |  |  |
| 1prtD | 0.714 | 0.814 | 0.614 | 0.833 | 0.453 | 0.7 | 35 | 40 | 8 | 22 | 144/238 | 144/238 |  |  |  |
| 1a88A | 0.845 | 0.5 | 0.59 | 0.892 | 0.451 | 0.541 | 23 | 189 | 23 | 16 | 99/163 | 99/163 |  |  |  |
| 1jqlB | 0.835 | 0.481 | 0.619 | 0.875 | 0.448 | 0.542 | 13 | 98 | 14 | 8 | 50/91 | 50/91 |  |  |  |
| 1g72A | 0.85 | 0.672 | 0.411 | 0.953 | 0.445 | 0.51 | 39 | 386 | 19 | 56 | 126/343 | 72/198 | 54/145 |  |  |
| 1l0oC | 0.722 | 0.741 | 0.714 | 0.731 | 0.445 | 0.727 | 20 | 19 | 7 | 8 | 72/107 | 72/107 |  |  |  |
| 1l6wA | 0.724 | 0.754 | 0.591 | 0.837 | 0.444 | 0.662 | 52 | 87 | 17 | 36 | 215/382 | 215/382 |  |  |  |
| 1mr1C | 0.688 | 0.364 | 0.941 | 0.632 | 0.443 | 0.525 | 16 | 48 | 28 | 1 | 77/89 | 77/89 |  |  |  |
| 1ynjK | 0.735 | 0.721 | 0.898 | 0.5 | 0.443 | 0.8 | 44 | 17 | 17 | 5 | 179/218 | 179/218 |  |  |  |
| 1azzC | 0.717 | 0.78 | 0.687 | 0.755 | 0.438 | 0.73 | 46 | 40 | 13 | 21 | 191/288 | 191/288 |  |  |  |
| 1jt6A | 0.756 | 0.452 | 0.757 | 0.755 | 0.437 | 0.566 | 28 | 105 | 34 | 9 | 104/161 | 104/161 |  |  |  |
| 1ad3A | 0.799 | 0.649 | 0.481 | 0.909 | 0.434 | 0.552 | 50 | 271 | 27 | 54 | 193/408 | 193/408 |  |  |  |
| 1bzyA | 0.767 | 0.635 | 0.559 | 0.858 | 0.434 | 0.595 | 33 | 115 | 19 | 26 | 108/264 | 108/264 |  |  |  |
| 1g0hA | 0.809 | 0.418 | 0.697 | 0.829 | 0.434 | 0.523 | 23 | 155 | 32 | 10 | 86/131 | 86/131 |  |  |  |
| 1h32B | 0.664 | 0.423 | 0.909 | 0.582 | 0.428 | 0.577 | 30 | 57 | 41 | 3 | 123/141 | 123/141 |  |  |  |
| 1fwxA | 0.777 | 0.716 | 0.433 | 0.926 | 0.426 | 0.54 | 68 | 336 | 27 | 89 | 264/665 | 264/665 |  |  |  |
| 1jzdA | 0.744 | 0.373 | 0.824 | 0.728 | 0.425 | 0.514 | 28 | 126 | 47 | 6 | 107/134 | 85/98 | 22/36 |  |  |
| 1f8uB | 0.69 | 0.833 | 0.5 | 0.893 | 0.424 | 0.625 | 15 | 25 | 3 | 15 | 69/146 | 69/146 |  |  |  |
| 1hg3A | 0.786 | 0.676 | 0.455 | 0.915 | 0.424 | 0.543 | 25 | 129 | 12 | 30 | 90/220 | 90/220 |  |  |  |
| 1kq4A | 0.688 | 0.578 | 0.84 | 0.586 | 0.424 | 0.685 | 63 | 65 | 46 | 12 | 246/327 | 246/327 |  |  |  |
| 1jw9B | 0.71 | 0.636 | 0.739 | 0.688 | 0.422 | 0.683 | 68 | 86 | 39 | 24 | 284/396 | 284/396 |  |  |  |
| 1bbhA | 0.659 | 0.339 | 0.955 | 0.594 | 0.421 | 0.5 | 21 | 60 | 41 | 1 | 83/86 | 83/86 |  |  |  |
| 1nksA | 0.765 | 0.456 | 0.703 | 0.782 | 0.421 | 0.553 | 26 | 111 | 31 | 11 | 103/152 | 103/152 |  |  |  |
| 1jjuA | 0.81 | 0.5 | 0.578 | 0.864 | 0.419 | 0.536 | 52 | 331 | 52 | 38 | 214/369 | 214/369 |  |  |  |
| 1gpwB | 0.815 | 0.395 | 0.68 | 0.836 | 0.418 | 0.5 | 17 | 133 | 26 | 8 | 76/117 | 76/117 |  |  |  |
| 1l1oC | 0.713 | 0.362 | 0.862 | 0.681 | 0.418 | 0.51 | 25 | 94 | 44 | 4 | 122/144 | 122/144 |  |  |  |
| 1fcjA | 0.84 | 0.5 | 0.524 | 0.9 | 0.416 | 0.512 | 22 | 198 | 22 | 20 | 89/188 | 89/188 |  |  |  |
| 1kkmA | 0.706 | 0.712 | 0.704 | 0.709 | 0.413 | 0.708 | 57 | 56 | 23 | 24 | 233/351 | 233/351 |  |  |  |
| 1nrjA | 0.777 | 0.421 | 0.696 | 0.794 | 0.411 | 0.525 | 16 | 85 | 22 | 7 | 65/113 | 65/113 |  |  |  |
| 1e5xA | 0.737 | 0.467 | 0.717 | 0.744 | 0.408 | 0.566 | 71 | 235 | 81 | 28 | 285/415 | 285/415 |  |  |  |
| 1gl4A | 0.798 | 0.3 | 0.808 | 0.797 | 0.407 | 0.438 | 21 | 192 | 49 | 5 | 89/114 | 89/114 |  |  |  |
| 1h0hB | 0.77 | 0.526 | 0.6 | 0.825 | 0.407 | 0.561 | 30 | 127 | 27 | 20 | 121/208 | 121/208 |  |  |  |
| 1kf6B | 0.671 | 0.847 | 0.48 | 0.897 | 0.407 | 0.613 | 61 | 96 | 11 | 66 | 227/499 | 227/499 |  |  |  |
| 1dowB | 0.774 | 0.8 | 0.909 | 0.444 | 0.406 | 0.851 | 20 | 4 | 5 | 2 | 93/114 | 93/114 |  |  |  |
| 1fqjC | 0.622 | 0.548 | 1 | 0.3 | 0.406 | 0.708 | 17 | 6 | 14 | 0 | 60/65 | 60/65 |  |  |  |
| 1jyoE | 0.755 | 0.739 | 0.986 | 0.273 | 0.406 | 0.845 | 68 | 9 | 24 | 1 | 313/332 | 313/332 |  |  |  |
| 1huxA | 0.79 | 0.233 | 0.933 | 0.78 | 0.403 | 0.373 | 14 | 163 | 46 | 1 | 46/57 | 46/57 |  |  |  |
| 1qo0A | 0.916 | 0.625 | 0.312 | 0.98 | 0.403 | 0.417 | 10 | 297 | 6 | 22 | 45/136 | 45/136 |  |  |  |
| 1jx2B | 0.807 | 0.224 | 0.938 | 0.799 | 0.402 | 0.361 | 15 | 207 | 52 | 1 | 56/67 | 56/67 |  |  |  |
| 1bvyF | 0.816 | 0.464 | 0.565 | 0.867 | 0.401 | 0.51 | 13 | 98 | 15 | 10 | 49/94 | 49/94 |  |  |  |
| 1lvoA | 0.816 | 0.446 | 0.581 | 0.862 | 0.4 | 0.505 | 25 | 193 | 31 | 18 | 86/139 | 86/139 |  |  |  |
| 1chmA | 0.808 | 0.63 | 0.41 | 0.928 | 0.398 | 0.496 | 34 | 256 | 20 | 49 | 154/364 | 154/364 |  |  |  |
| 1cg5A | 0.681 | 0.507 | 0.8 | 0.624 | 0.397 | 0.621 | 36 | 58 | 35 | 9 | 138/189 | 138/189 |  |  |  |
| 1li1A | 0.712 | 0.762 | 0.762 | 0.634 | 0.396 | 0.762 | 96 | 52 | 30 | 30 | 378/553 | 378/553 |  |  |  |
| 1n1jB | 0.711 | 0.689 | 0.933 | 0.387 | 0.396 | 0.792 | 42 | 12 | 19 | 3 | 206/216 | 206/216 |  |  |  |
| 1qfhA | 0.747 | 0.559 | 0.589 | 0.812 | 0.395 | 0.574 | 33 | 112 | 26 | 23 | 134/243 | 134/243 |  |  |  |
| 1h9sA | 0.758 | 0.594 | 0.528 | 0.852 | 0.394 | 0.559 | 19 | 75 | 13 | 17 | 67/151 | 67/151 |  |  |  |
| 1f75A | 0.722 | 0.359 | 0.8 | 0.706 | 0.392 | 0.496 | 28 | 120 | 50 | 7 | 133/182 | 133/182 |  |  |  |
| 1m4uA | 0.663 | 0.495 | 0.833 | 0.579 | 0.391 | 0.621 | 55 | 77 | 56 | 11 | 240/294 | 131/158 | 109/136 |  |  |
| 1ynjD | 0.738 | 0.547 | 0.614 | 0.789 | 0.391 | 0.579 | 202 | 626 | 167 | 127 | 843/1423 | 832/1409 | 11/14 |  |  |
| 1ld8B | 0.74 | 0.492 | 0.652 | 0.77 | 0.389 | 0.56 | 58 | 201 | 60 | 31 | 194/374 | 194/374 |  |  |  |
| 1nf3C | 0.605 | 0.343 | 0.958 | 0.511 | 0.389 | 0.505 | 23 | 46 | 44 | 1 | 105/116 | 105/116 |  |  |  |
| 1ia9A | 0.677 | 0.481 | 0.797 | 0.624 | 0.388 | 0.6 | 63 | 113 | 68 | 16 | 260/362 | 260/362 |  |  |  |
| 2vglA | 0.76 | 0.509 | 0.593 | 0.815 | 0.388 | 0.548 | 83 | 352 | 80 | 57 | 309/564 | 309/564 |  |  |  |
| 1e9gA | 0.891 | 0.379 | 0.524 | 0.923 | 0.387 | 0.44 | 11 | 217 | 18 | 10 | 65/112 | 65/112 |  |  |  |
| 1gl4B | 0.671 | 0.439 | 0.818 | 0.617 | 0.385 | 0.571 | 18 | 37 | 23 | 4 | 72/100 | 72/100 |  |  |  |
| 1ftrA | 0.715 | 0.663 | 0.539 | 0.826 | 0.383 | 0.595 | 55 | 133 | 28 | 47 | 200/410 | 200/410 |  |  |  |
| 1i9bA | 0.739 | 0.607 | 0.531 | 0.837 | 0.383 | 0.567 | 34 | 113 | 22 | 30 | 127/265 | 127/265 |  |  |  |
| 1mjgM | 0.91 | 0.44 | 0.423 | 0.953 | 0.383 | 0.431 | 22 | 566 | 28 | 30 | 80/223 | 80/223 |  |  |  |
| 1d7aA | 0.695 | 0.792 | 0.693 | 0.698 | 0.381 | 0.739 | 61 | 37 | 16 | 27 | 250/390 | 250/390 |  |  |  |
| 1tx4A | 0.797 | 0.419 | 0.6 | 0.836 | 0.38 | 0.493 | 18 | 127 | 25 | 12 | 65/113 | 65/113 |  |  |  |
| 1gpqA | 0.729 | 0.595 | 0.564 | 0.81 | 0.379 | 0.579 | 22 | 64 | 15 | 17 | 87/180 | 87/180 |  |  |  |
| 1hiaI | 0.553 | 0.417 | 1 | 0.344 | 0.378 | 0.588 | 15 | 11 | 21 | 0 | 76/77 | 76/77 |  |  |  |
| 1fxkA | 0.689 | 0.435 | 0.769 | 0.662 | 0.377 | 0.556 | 20 | 51 | 26 | 6 | 84/119 | 72/107 | 12/12 |  |  |
| 1icfI | 0.641 | 0.487 | 0.864 | 0.524 | 0.377 | 0.623 | 19 | 22 | 20 | 3 | 70/104 | 70/104 |  |  |  |
| 1lqpA | 0.685 | 0.654 | 0.785 | 0.585 | 0.377 | 0.713 | 51 | 38 | 27 | 14 | 226/276 | 226/276 |  |  |  |
| 1e0fI | 0.673 | 0.581 | 0.783 | 0.594 | 0.374 | 0.667 | 18 | 19 | 13 | 5 | 75/95 | 75/95 |  |  |  |
| 1hq3D | 0.765 | 0.764 | 0.965 | 0.292 | 0.373 | 0.853 | 55 | 7 | 17 | 2 | 252/276 | 252/276 |  |  |  |
| 1do8A | 0.834 | 0.31 | 0.66 | 0.852 | 0.372 | 0.422 | 31 | 396 | 69 | 16 | 127/225 | 127/225 |  |  |  |
| 1jv2A | 0.813 | 0.305 | 0.69 | 0.827 | 0.371 | 0.423 | 58 | 631 | 132 | 26 | 200/310 | 151/218 | 42/66 | 7/26 |  |
| 1prtF | 0.688 | 0.678 | 0.784 | 0.578 | 0.371 | 0.727 | 40 | 26 | 19 | 11 | 169/220 | 169/220 |  |  |  |
| 1h32A | 0.687 | 0.248 | 0.926 | 0.658 | 0.37 | 0.391 | 25 | 146 | 76 | 2 | 91/124 | 91/124 |  |  |  |
| 1lr5A | 0.669 | 0.4 | 0.811 | 0.625 | 0.37 | 0.536 | 30 | 75 | 45 | 7 | 100/134 | 100/134 |  |  |  |
| 1fltX | 0.656 | 0.364 | 0.842 | 0.606 | 0.366 | 0.508 | 16 | 43 | 28 | 3 | 60/81 | 60/81 |  |  |  |
| 1ldjB | 0.635 | 0.534 | 0.886 | 0.46 | 0.365 | 0.667 | 31 | 23 | 27 | 4 | 203/224 | 203/224 |  |  |  |
| 1qo0D | 0.659 | 0.564 | 0.814 | 0.546 | 0.364 | 0.667 | 57 | 53 | 44 | 13 | 235/316 | 235/316 |  |  |  |
| 1agqA | 0.6 | 0.493 | 0.943 | 0.382 | 0.363 | 0.647 | 33 | 21 | 34 | 2 | 118/140 | 118/140 |  |  |  |
| 1lj2A | 0.731 | 0.733 | 0.926 | 0.361 | 0.362 | 0.818 | 63 | 13 | 23 | 5 | 304/350 | 304/350 |  |  |  |
| 1g4yB | 0.775 | 0.828 | 0.883 | 0.45 | 0.361 | 0.855 | 53 | 9 | 11 | 7 | 251/309 | 251/309 |  |  |  |
| 1i4dA | 0.624 | 0.487 | 0.877 | 0.483 | 0.361 | 0.626 | 57 | 56 | 60 | 8 | 249/282 | 249/282 |  |  |  |
| 1k83C | 0.679 | 0.708 | 0.625 | 0.734 | 0.361 | 0.664 | 80 | 91 | 33 | 48 | 350/580 | 350/580 |  |  |  |
| 1ll0A | 0.715 | 0.307 | 0.794 | 0.702 | 0.36 | 0.443 | 27 | 144 | 61 | 7 | 102/130 | 102/130 |  |  |  |
| 1gh6B | 0.768 | 0.235 | 0.833 | 0.763 | 0.359 | 0.367 | 20 | 209 | 65 | 4 | 59/91 | 59/91 |  |  |  |
| 1d4fA | 0.735 | 0.56 | 0.53 | 0.822 | 0.358 | 0.545 | 61 | 222 | 48 | 54 | 253/507 | 253/507 |  |  |  |
| 1hulA | 0.774 | 0.807 | 0.91 | 0.393 | 0.356 | 0.855 | 71 | 11 | 17 | 7 | 315/378 | 315/378 |  |  |  |
| 1prtA | 0.76 | 0.481 | 0.542 | 0.825 | 0.352 | 0.51 | 26 | 132 | 28 | 22 | 107/200 | 107/200 |  |  |  |
| 1f45A | 0.773 | 0.247 | 0.783 | 0.772 | 0.35 | 0.375 | 18 | 186 | 55 | 5 | 77/104 | 77/104 |  |  |  |
| 1jxhA | 0.714 | 0.443 | 0.66 | 0.732 | 0.35 | 0.53 | 35 | 120 | 44 | 18 | 147/231 | 147/231 |  |  |  |
| 1jjuC | 0.776 | 0.929 | 0.8 | 0.636 | 0.349 | 0.86 | 52 | 7 | 4 | 13 | 203/311 | 203/311 |  |  |  |
| 1jy2P | 0.814 | 0.842 | 0.941 | 0.333 | 0.348 | 0.889 | 32 | 3 | 6 | 2 | 163/172 | 163/172 |  |  |  |
| 1k1dA | 0.846 | 0.424 | 0.446 | 0.907 | 0.346 | 0.435 | 25 | 331 | 34 | 31 | 88/222 | 50/144 | 38/78 |  |  |
| 1i2mB | 0.813 | 0.605 | 0.324 | 0.944 | 0.345 | 0.422 | 23 | 251 | 15 | 48 | 76/272 | 76/272 |  |  |  |
| 1ixsB | 0.783 | 0.205 | 0.842 | 0.779 | 0.345 | 0.33 | 16 | 219 | 62 | 3 | 53/66 | 53/66 |  |  |  |
| 1hx3A | 0.762 | 0.319 | 0.682 | 0.775 | 0.344 | 0.435 | 15 | 110 | 32 | 7 | 57/87 | 57/87 |  |  |  |
| 1k2fA | 0.644 | 0.274 | 0.885 | 0.604 | 0.344 | 0.418 | 23 | 93 | 61 | 3 | 81/117 | 81/117 |  |  |  |
| 1kxpD | 0.857 | 0.386 | 0.468 | 0.906 | 0.344 | 0.423 | 22 | 337 | 35 | 25 | 72/183 | 72/183 |  |  |  |
| 1h6kA | 0.854 | 0.216 | 0.727 | 0.86 | 0.343 | 0.333 | 24 | 536 | 87 | 9 | 77/131 | 77/131 |  |  |  |
| 1sgpE | 0.762 | 0.356 | 0.64 | 0.785 | 0.343 | 0.457 | 16 | 106 | 29 | 9 | 34/77 | 34/77 |  |  |  |
| 1hssA | 0.69 | 0.488 | 0.667 | 0.7 | 0.342 | 0.563 | 20 | 49 | 21 | 10 | 65/110 | 65/110 |  |  |  |
| 1jy2O | 0.784 | 0.854 | 0.875 | 0.455 | 0.341 | 0.864 | 35 | 5 | 6 | 5 | 169/198 | 169/198 |  |  |  |
| 2vglS | 0.619 | 0.854 | 0.482 | 0.857 | 0.341 | 0.617 | 41 | 42 | 7 | 44 | 160/354 | 160/354 |  |  |  |
| 1h4lD | 0.687 | 0.464 | 0.684 | 0.688 | 0.34 | 0.553 | 26 | 66 | 30 | 12 | 105/155 | 105/155 |  |  |  |
| 1g5hA | 0.703 | 0.393 | 0.695 | 0.705 | 0.339 | 0.502 | 57 | 210 | 88 | 25 | 221/333 | 221/333 |  |  |  |
| 1gt7A | 0.693 | 0.586 | 0.573 | 0.763 | 0.338 | 0.58 | 51 | 116 | 36 | 38 | 223/394 | 223/394 |  |  |  |
| 1a4xA | 0.818 | 0.393 | 0.5 | 0.871 | 0.337 | 0.44 | 11 | 115 | 17 | 11 | 41/85 | 41/85 |  |  |  |
| 1hzpA | 0.714 | 0.511 | 0.578 | 0.77 | 0.337 | 0.542 | 48 | 154 | 46 | 35 | 202/330 | 202/330 |  |  |  |
| 1klfB | 0.637 | 0.294 | 0.86 | 0.594 | 0.337 | 0.438 | 37 | 130 | 89 | 6 | 129/154 | 129/154 |  |  |  |
| 1l1oB | 0.658 | 0.548 | 0.739 | 0.606 | 0.337 | 0.63 | 34 | 43 | 28 | 12 | 137/201 | 137/201 |  |  |  |
| 1gl2A | 0.824 | 0.848 | 0.951 | 0.3 | 0.335 | 0.897 | 39 | 3 | 7 | 2 | 178/212 | 178/212 |  |  |  |
| 3ygsP | 0.713 | 0.8 | 0.258 | 0.964 | 0.334 | 0.39 | 8 | 54 | 2 | 23 | 40/152 | 40/152 |  |  |  |
| 1gh6A | 0.649 | 0.26 | 0.867 | 0.615 | 0.331 | 0.4 | 13 | 59 | 37 | 2 | 70/80 | 70/80 |  |  |  |
| 1d2zA | 0.737 | 0.5 | 0.52 | 0.814 | 0.33 | 0.51 | 13 | 57 | 13 | 12 | 44/104 | 44/104 |  |  |  |
| 1a38A | 0.716 | 0.394 | 0.651 | 0.733 | 0.329 | 0.491 | 28 | 118 | 43 | 15 | 78/145 | 53/97 | 25/48 |  |  |
| 1bh9B | 0.565 | 0.486 | 0.971 | 0.28 | 0.325 | 0.648 | 34 | 14 | 36 | 1 | 139/147 | 139/147 |  |  |  |
| 1k90A | 0.751 | 0.495 | 0.477 | 0.841 | 0.322 | 0.486 | 53 | 285 | 54 | 58 | 170/426 | 155/332 | 15/94 |  |  |
| 1e2tA | 0.722 | 0.326 | 0.683 | 0.729 | 0.32 | 0.441 | 28 | 156 | 58 | 13 | 115/174 | 115/174 |  |  |  |
| 1dqnA | 0.785 | 0.486 | 0.419 | 0.883 | 0.319 | 0.45 | 18 | 143 | 19 | 25 | 66/183 | 66/183 |  |  |  |
| 1k20A | 0.839 | 0.298 | 0.538 | 0.87 | 0.317 | 0.384 | 14 | 220 | 33 | 12 | 66/121 | 66/121 |  |  |  |
| 1jqlA | 0.841 | 0.271 | 0.571 | 0.865 | 0.316 | 0.368 | 16 | 275 | 43 | 12 | 64/109 | 64/109 |  |  |  |
| 1a0fA | 0.78 | 0.516 | 0.39 | 0.894 | 0.315 | 0.444 | 16 | 126 | 15 | 25 | 69/172 | 69/172 |  |  |  |
| 1dmlB | 0.8 | 0.794 | 1 | 0.125 | 0.315 | 0.885 | 27 | 1 | 7 | 0 | 121/125 | 121/125 |  |  |  |
| 1jy2N | 0.791 | 0.816 | 0.939 | 0.3 | 0.315 | 0.873 | 31 | 3 | 7 | 2 | 167/182 | 167/182 |  |  |  |
| 1bvnT | 0.642 | 0.486 | 0.739 | 0.591 | 0.314 | 0.586 | 17 | 26 | 18 | 6 | 89/116 | 89/116 |  |  |  |
| 1ci6B | 0.644 | 0.619 | 1 | 0.158 | 0.313 | 0.765 | 26 | 3 | 16 | 0 | 107/125 | 107/125 |  |  |  |
| 1he1A | 0.768 | 0.522 | 0.4 | 0.884 | 0.313 | 0.453 | 12 | 84 | 11 | 18 | 42/105 | 42/105 |  |  |  |
| 1kjyB | 0.829 | 0.871 | 0.931 | 0.333 | 0.313 | 0.9 | 27 | 2 | 4 | 2 | 122/150 | 122/150 |  |  |  |
| 1nmmB | 0.758 | 0.188 | 0.812 | 0.754 | 0.312 | 0.306 | 13 | 172 | 56 | 3 | 51/68 | 51/68 |  |  |  |
| 1jb0D | 0.623 | 0.78 | 0.444 | 0.845 | 0.309 | 0.566 | 32 | 49 | 9 | 40 | 133/306 | 133/306 |  |  |  |
| 1mjhA | 0.761 | 0.382 | 0.542 | 0.809 | 0.309 | 0.448 | 13 | 89 | 21 | 11 | 58/108 | 58/108 |  |  |  |
| 1jkgB | 0.632 | 0.44 | 0.755 | 0.579 | 0.307 | 0.556 | 40 | 70 | 51 | 13 | 142/203 | 142/203 |  |  |  |
| 1kshB | 0.757 | 0.361 | 0.565 | 0.796 | 0.307 | 0.441 | 13 | 90 | 23 | 10 | 47/88 | 47/88 |  |  |  |
| 1cmxA | 0.693 | 0.432 | 0.615 | 0.72 | 0.304 | 0.508 | 32 | 108 | 42 | 20 | 112/211 | 112/211 |  |  |  |
| 1o6sA | 0.87 | 0.5 | 0.269 | 0.96 | 0.302 | 0.35 | 14 | 334 | 14 | 38 | 55/183 | 55/183 |  |  |  |
| 1bd3A | 0.667 | 0.589 | 0.558 | 0.739 | 0.3 | 0.573 | 43 | 85 | 30 | 34 | 157/331 | 157/331 |  |  |  |
| 1cruA | 0.908 | 0.4 | 0.303 | 0.96 | 0.299 | 0.345 | 10 | 363 | 15 | 23 | 31/124 | 31/124 |  |  |  |
| 1k3sA | 0.587 | 0.387 | 0.828 | 0.493 | 0.293 | 0.527 | 24 | 37 | 38 | 5 | 96/117 | 96/117 |  |  |  |
| 1nbwA | 0.74 | 0.323 | 0.596 | 0.767 | 0.291 | 0.419 | 53 | 365 | 111 | 36 | 182/341 | 108/201 | 74/140 |  |  |
| 3c98A | 0.826 | 0.35 | 0.438 | 0.882 | 0.291 | 0.389 | 28 | 390 | 52 | 36 | 113/272 | 38/57 | 75/215 |  |  |
| 1lm8C | 0.655 | 0.661 | 0.787 | 0.486 | 0.288 | 0.718 | 37 | 18 | 19 | 10 | 163/224 | 163/224 |  |  |  |
| 1m3eA | 0.799 | 0.337 | 0.483 | 0.849 | 0.288 | 0.397 | 29 | 321 | 57 | 31 | 90/241 | 90/241 |  |  |  |
| 1dowA | 0.643 | 0.303 | 0.75 | 0.62 | 0.286 | 0.432 | 27 | 101 | 62 | 9 | 82/129 | 82/129 |  |  |  |
| 2rspA | 0.56 | 0.362 | 0.862 | 0.45 | 0.286 | 0.51 | 25 | 36 | 44 | 4 | 109/143 | 109/143 |  |  |  |
| 1f80A | 0.641 | 0.577 | 0.667 | 0.621 | 0.285 | 0.619 | 30 | 36 | 22 | 15 | 119/202 | 119/202 |  |  |  |
| 1k28D | 0.652 | 0.358 | 0.701 | 0.638 | 0.285 | 0.474 | 54 | 171 | 97 | 23 | 191/321 | 191/321 |  |  |  |
| 1bdmA | 0.818 | 0.348 | 0.432 | 0.877 | 0.282 | 0.386 | 16 | 213 | 30 | 21 | 76/171 | 76/171 |  |  |  |
| 1o6sB | 0.7 | 0.56 | 0.424 | 0.836 | 0.282 | 0.483 | 14 | 56 | 11 | 19 | 46/128 | 46/128 |  |  |  |
| 1jpyA | 0.683 | 0.683 | 0.92 | 0.289 | 0.277 | 0.784 | 69 | 13 | 32 | 6 | 295/357 | 295/357 |  |  |  |
| 1flmA | 0.664 | 0.391 | 0.643 | 0.671 | 0.275 | 0.486 | 18 | 57 | 28 | 10 | 61/113 | 61/113 |  |  |  |
| 1dekA | 0.757 | 0.233 | 0.636 | 0.77 | 0.273 | 0.341 | 14 | 154 | 46 | 8 | 50/84 | 50/84 |  |  |  |
| 1hyhA | 0.692 | 0.529 | 0.451 | 0.807 | 0.27 | 0.487 | 37 | 138 | 33 | 45 | 126/356 | 126/356 |  |  |  |
| 1ihoA | 0.798 | 0.288 | 0.5 | 0.838 | 0.27 | 0.366 | 15 | 191 | 37 | 15 | 64/130 | 64/130 |  |  |  |
| 1gyxA | 0.662 | 0.661 | 0.886 | 0.333 | 0.268 | 0.757 | 39 | 10 | 20 | 5 | 155/192 | 155/192 |  |  |  |
| 1kmiZ | 0.599 | 0.567 | 0.967 | 0.2 | 0.264 | 0.715 | 89 | 17 | 68 | 3 | 338/385 | 316/358 | 22/27 |  |  |
| 1o94D | 0.652 | 0.455 | 0.6 | 0.676 | 0.259 | 0.517 | 30 | 75 | 36 | 20 | 116/219 | 116/219 |  |  |  |
| 1bo1A | 0.782 | 0.246 | 0.548 | 0.809 | 0.258 | 0.34 | 17 | 220 | 52 | 14 | 69/130 | 69/130 |  |  |  |
| 1mpyA | 0.688 | 0.448 | 0.506 | 0.759 | 0.256 | 0.476 | 39 | 151 | 48 | 38 | 164/329 | 164/329 |  |  |  |
| 1tiiD | 0.649 | 0.667 | 0.8 | 0.436 | 0.254 | 0.727 | 44 | 17 | 22 | 11 | 179/243 | 179/243 |  |  |  |
| 3sdhA | 0.729 | 0.3 | 0.545 | 0.763 | 0.248 | 0.387 | 12 | 90 | 28 | 10 | 51/93 | 51/93 |  |  |  |
| 1jv2B | 0.779 | 0.317 | 0.446 | 0.836 | 0.247 | 0.371 | 33 | 362 | 71 | 41 | 126/295 | 62/197 | 39/73 | 25/25 |  |
| 1miuB | 0.744 | 0.964 | 0.75 | 0.667 | 0.247 | 0.844 | 27 | 2 | 1 | 9 | 165/236 | 165/236 |  |  |  |
| 1b34B | 0.634 | 0.659 | 0.725 | 0.516 | 0.246 | 0.69 | 29 | 16 | 15 | 11 | 105/163 | 105/163 |  |  |  |
| 1otgA | 0.629 | 0.639 | 0.73 | 0.509 | 0.246 | 0.681 | 46 | 27 | 26 | 17 | 197/278 | 197/278 |  |  |  |
| 1nbaA | 0.602 | 0.719 | 0.357 | 0.856 | 0.244 | 0.477 | 41 | 95 | 16 | 74 | 165/517 | 165/517 |  |  |  |
| 2eboA | 0.712 | 0.73 | 0.92 | 0.261 | 0.244 | 0.814 | 46 | 6 | 17 | 4 | 208/249 | 208/249 |  |  |  |
| 1mbxC | 0.45 | 0.295 | 0.947 | 0.295 | 0.242 | 0.45 | 18 | 18 | 43 | 1 | 68/77 | 68/77 |  |  |  |
| 1jv1A | 0.783 | 0.304 | 0.438 | 0.838 | 0.239 | 0.359 | 28 | 332 | 64 | 36 | 92/262 | 92/262 |  |  |  |
| 1g31A | 0.57 | 0.478 | 0.825 | 0.4 | 0.238 | 0.606 | 33 | 24 | 36 | 7 | 113/156 | 113/156 |  |  |  |
| 1h0hA | 0.915 | 0.326 | 0.246 | 0.963 | 0.238 | 0.28 | 14 | 758 | 29 | 43 | 61/221 | 61/221 |  |  |  |
| 1juhA | 0.749 | 0.352 | 0.439 | 0.819 | 0.237 | 0.391 | 25 | 208 | 46 | 32 | 102/244 | 93/234 | 9/10 |  |  |
| 2ccyA | 0.625 | 0.245 | 0.722 | 0.608 | 0.237 | 0.366 | 13 | 62 | 40 | 5 | 41/84 | 41/84 |  |  |  |
| 1fo0B | 0.62 | 0.36 | 0.667 | 0.605 | 0.236 | 0.468 | 18 | 49 | 32 | 9 | 92/140 | 92/140 |  |  |  |
| 1gx1A | 0.639 | 0.492 | 0.558 | 0.684 | 0.236 | 0.523 | 29 | 65 | 30 | 23 | 91/205 | 91/205 |  |  |  |
| 1lhpA | 0.732 | 0.32 | 0.49 | 0.783 | 0.234 | 0.387 | 24 | 184 | 51 | 25 | 105/209 | 105/209 |  |  |  |
| 1ek9A | 0.659 | 0.424 | 0.542 | 0.706 | 0.233 | 0.476 | 64 | 209 | 87 | 54 | 247/517 | 247/517 |  |  |  |
| 1qgwA | 0.797 | 0.859 | 0.902 | 0.308 | 0.233 | 0.88 | 55 | 4 | 9 | 6 | 244/307 | 244/307 |  |  |  |
| 1t83C | 0.648 | 0.2 | 0.722 | 0.639 | 0.232 | 0.313 | 13 | 92 | 52 | 5 | 62/93 | 62/93 |  |  |  |
| 1m7gA | 0.741 | 0.216 | 0.579 | 0.759 | 0.23 | 0.314 | 11 | 126 | 40 | 8 | 34/82 | 34/82 |  |  |  |
| 1mg2D | 0.732 | 0.195 | 0.615 | 0.744 | 0.229 | 0.296 | 8 | 96 | 33 | 5 | 31/53 | 31/53 |  |  |  |
| 1b6sA | 0.86 | 0.333 | 0.278 | 0.932 | 0.227 | 0.303 | 10 | 273 | 20 | 26 | 30/132 | 30/132 |  |  |  |
| 1ewyC | 0.67 | 0.588 | 0.294 | 0.883 | 0.222 | 0.392 | 10 | 53 | 7 | 24 | 40/137 | 40/137 |  |  |  |
| 1abrB | 0.636 | 0.26 | 0.659 | 0.632 | 0.218 | 0.372 | 27 | 132 | 77 | 14 | 112/183 | 112/183 |  |  |  |
| 1ihrA | 0.836 | 0.855 | 0.967 | 0.167 | 0.218 | 0.908 | 59 | 2 | 10 | 2 | 299/333 | 299/333 |  |  |  |
| 1ci6A | 0.577 | 0.537 | 0.88 | 0.296 | 0.216 | 0.667 | 22 | 8 | 19 | 3 | 98/118 | 98/118 |  |  |  |
| 1jnrB | 0.649 | 0.735 | 0.735 | 0.48 | 0.215 | 0.735 | 72 | 24 | 26 | 26 | 351/494 | 351/494 |  |  |  |
| 1mt5A | 0.717 | 0.182 | 0.615 | 0.727 | 0.213 | 0.281 | 24 | 287 | 108 | 15 | 97/172 | 97/172 |  |  |  |
| 1f3vA | 0.836 | 0.333 | 0.278 | 0.918 | 0.212 | 0.303 | 5 | 112 | 10 | 13 | 17/79 | 17/79 |  |  |  |
| 1k83L | 0.523 | 0.5 | 1 | 0.087 | 0.209 | 0.667 | 21 | 2 | 21 | 0 | 116/121 | 116/121 |  |  |  |
| 1nbwB | 0.65 | 0.378 | 0.538 | 0.689 | 0.207 | 0.444 | 14 | 51 | 23 | 12 | 66/127 | 66/127 |  |  |  |
| 1mz9A | 0.841 | 0.857 | 0.973 | 0.143 | 0.203 | 0.911 | 36 | 1 | 6 | 1 | 178/192 | 178/192 |  |  |  |
| 1fyhB | 0.647 | 0.233 | 0.63 | 0.65 | 0.201 | 0.34 | 17 | 104 | 56 | 10 | 50/99 | 50/99 |  |  |  |
| 1bi7B | 0.636 | 0.514 | 0.439 | 0.754 | 0.2 | 0.474 | 18 | 52 | 17 | 23 | 65/152 | 65/152 |  |  |  |
| 1aa7A | 0.715 | 0.297 | 0.458 | 0.77 | 0.195 | 0.361 | 11 | 87 | 26 | 13 | 47/108 | 47/108 |  |  |  |
| 1ktzB | 0.667 | 0.333 | 0.5 | 0.714 | 0.189 | 0.4 | 11 | 55 | 22 | 11 | 45/100 | 45/100 |  |  |  |
| 1gl2C | 0.717 | 0.809 | 0.826 | 0.357 | 0.188 | 0.817 | 38 | 5 | 9 | 8 | 172/225 | 172/225 |  |  |  |
| 1fs1B | 0.604 | 0.42 | 0.583 | 0.613 | 0.185 | 0.488 | 21 | 46 | 29 | 15 | 85/146 | 76/85 | 9/61 |  |  |
| 1gl2D | 0.769 | 0.792 | 0.95 | 0.167 | 0.184 | 0.864 | 38 | 2 | 10 | 2 | 173/195 | 173/195 |  |  |  |
| 1hq3C | 0.667 | 0.679 | 0.917 | 0.212 | 0.184 | 0.78 | 55 | 7 | 26 | 5 | 255/298 | 255/298 |  |  |  |
| 1cd9B | 0.684 | 0.234 | 0.536 | 0.708 | 0.182 | 0.326 | 15 | 119 | 49 | 13 | 62/118 | 40/92 | 22/26 |  |  |
| 1dbqA | 0.782 | 0.308 | 0.316 | 0.868 | 0.182 | 0.312 | 12 | 178 | 27 | 26 | 49/168 | 49/168 |  |  |  |
| 1f9aA | 0.658 | 0.462 | 0.383 | 0.788 | 0.18 | 0.419 | 18 | 78 | 21 | 29 | 76/187 | 76/187 |  |  |  |
| 1clvI | 0.645 | 0.696 | 0.8 | 0.364 | 0.179 | 0.744 | 16 | 4 | 7 | 4 | 77/98 | 77/98 |  |  |  |
| 1tocR | 0.59 | 0.474 | 0.6 | 0.583 | 0.178 | 0.529 | 27 | 42 | 30 | 18 | 92/179 | 92/179 |  |  |  |
| 1gc1C | 0.798 | 0.292 | 0.292 | 0.882 | 0.174 | 0.292 | 7 | 127 | 17 | 17 | 27/98 | 27/98 |  |  |  |
| 1e5dA | 0.728 | 0.234 | 0.449 | 0.771 | 0.172 | 0.308 | 22 | 243 | 72 | 27 | 96/196 | 96/196 |  |  |  |
| 1nrjB | 0.8 | 0.318 | 0.259 | 0.899 | 0.172 | 0.286 | 7 | 133 | 15 | 20 | 32/101 | 32/101 |  |  |  |
| 1azsA | 0.558 | 0.367 | 0.673 | 0.509 | 0.167 | 0.475 | 33 | 59 | 57 | 16 | 143/225 | 130/204 | 13/21 |  |  |
| 1gl0I | 0.613 | 0.625 | 0.833 | 0.308 | 0.166 | 0.714 | 15 | 4 | 9 | 3 | 68/80 | 68/80 |  |  |  |
| 1k8rB | 0.5 | 0.213 | 0.769 | 0.448 | 0.163 | 0.333 | 10 | 30 | 37 | 3 | 30/56 | 30/56 |  |  |  |
| 1go3E | 0.53 | 0.4 | 0.745 | 0.418 | 0.161 | 0.521 | 38 | 41 | 57 | 13 | 143/215 | 143/215 |  |  |  |
| 1jmaA | 0.664 | 0.212 | 0.529 | 0.685 | 0.156 | 0.303 | 18 | 146 | 67 | 16 | 62/130 | 62/130 |  |  |  |
| 1f39A | 0.576 | 0.469 | 0.59 | 0.567 | 0.153 | 0.523 | 23 | 34 | 26 | 16 | 85/146 | 85/146 |  |  |  |
| 1gl2B | 0.814 | 0.825 | 0.979 | 0.091 | 0.151 | 0.895 | 47 | 1 | 10 | 1 | 221/239 | 221/239 |  |  |  |
| 1kacB | 0.733 | 0.333 | 0.292 | 0.848 | 0.147 | 0.311 | 7 | 78 | 14 | 17 | 24/90 | 24/90 |  |  |  |
| 1eerA | 0.658 | 0.327 | 0.432 | 0.727 | 0.146 | 0.372 | 16 | 88 | 33 | 21 | 61/168 | 61/168 |  |  |  |
| 1hx1B | 0.699 | 0.348 | 0.333 | 0.81 | 0.146 | 0.34 | 8 | 64 | 15 | 16 | 23/95 | 23/95 |  |  |  |
| 1im3D | 0.473 | 0.237 | 0.778 | 0.4 | 0.146 | 0.364 | 14 | 30 | 45 | 4 | 49/68 | 49/68 |  |  |  |
| 1ghqB | 0.555 | 0.343 | 0.639 | 0.522 | 0.145 | 0.447 | 23 | 48 | 44 | 13 | 73/143 | 51/94 | 22/49 |  |  |
| 1im9D | 0.679 | 0.167 | 0.526 | 0.697 | 0.145 | 0.253 | 10 | 115 | 50 | 9 | 43/75 | 43/75 |  |  |  |
| 1miuA | 0.648 | 0.258 | 0.491 | 0.683 | 0.142 | 0.338 | 57 | 354 | 164 | 59 | 221/454 | 214/395 | 7/59 |  |  |
| 1e96B | 0.671 | 0.164 | 0.529 | 0.687 | 0.14 | 0.25 | 9 | 101 | 46 | 8 | 25/65 | 25/65 |  |  |  |
| 1preA | 0.759 | 0.247 | 0.323 | 0.833 | 0.14 | 0.28 | 20 | 305 | 61 | 42 | 93/257 | 0/11 | 0/64 | 66/120 | 27/62 |
| 1kilE | 0.575 | 0.5 | 0.529 | 0.609 | 0.137 | 0.514 | 9 | 14 | 9 | 8 | 50/78 | 50/78 |  |  |  |
| 1f02T | 0.547 | 0.714 | 0.488 | 0.652 | 0.135 | 0.58 | 20 | 15 | 8 | 21 | 57/163 | 57/163 |  |  |  |
| 1gzsB | 0.609 | 0.362 | 0.488 | 0.657 | 0.135 | 0.416 | 21 | 71 | 37 | 22 | 60/161 | 60/161 |  |  |  |
| 1l2wI | 0.789 | 0.8 | 0.978 | 0.083 | 0.135 | 0.88 | 44 | 1 | 11 | 1 | 201/215 | 201/215 |  |  |  |
| 1jthA | 0.793 | 0.863 | 0.898 | 0.222 | 0.134 | 0.88 | 44 | 2 | 7 | 5 | 213/249 | 213/249 |  |  |  |
| 1foeA | 0.763 | 0.181 | 0.361 | 0.81 | 0.128 | 0.241 | 13 | 251 | 59 | 23 | 43/168 | 43/168 |  |  |  |
| 1fjrA | 0.648 | 0.226 | 0.483 | 0.68 | 0.126 | 0.308 | 14 | 102 | 48 | 15 | 39/91 | 39/91 |  |  |  |
| 1g73C | 0.6 | 0.444 | 0.414 | 0.706 | 0.122 | 0.429 | 12 | 36 | 15 | 17 | 38/113 | 38/113 |  |  |  |
| 1gl1I | 0.576 | 0.619 | 0.684 | 0.429 | 0.116 | 0.65 | 13 | 6 | 8 | 6 | 52/80 | 52/80 |  |  |  |
| 1eerB | 0.632 | 0.171 | 0.52 | 0.648 | 0.114 | 0.257 | 13 | 116 | 63 | 12 | 44/104 | 44/104 |  |  |  |
| 1kveB | 0.724 | 0.797 | 0.864 | 0.235 | 0.114 | 0.829 | 51 | 4 | 13 | 8 | 192/257 | 192/257 |  |  |  |
| 1afrA | 0.755 | 0.367 | 0.155 | 0.924 | 0.113 | 0.218 | 11 | 232 | 19 | 60 | 43/289 | 43/289 |  |  |  |
| 2trcP | 0.564 | 0.321 | 0.562 | 0.565 | 0.113 | 0.409 | 27 | 74 | 57 | 21 | 115/219 | 115/219 |  |  |  |
| 1ev7A | 0.818 | 0.194 | 0.233 | 0.886 | 0.11 | 0.212 | 7 | 226 | 29 | 23 | 20/136 | 20/136 |  |  |  |
| 1ospO | 0.859 | 0.19 | 0.182 | 0.925 | 0.109 | 0.186 | 4 | 210 | 17 | 18 | 11/82 | 11/82 |  |  |  |
| 1fm9D | 0.661 | 0.241 | 0.413 | 0.716 | 0.107 | 0.304 | 19 | 151 | 60 | 27 | 61/183 | 61/183 |  |  |  |
| 1g73A | 0.458 | 0.321 | 0.778 | 0.327 | 0.104 | 0.455 | 35 | 36 | 74 | 10 | 121/169 | 96/144 | 25/25 |  |  |
| 1nbfA | 0.772 | 0.25 | 0.226 | 0.873 | 0.104 | 0.238 | 12 | 248 | 36 | 41 | 52/219 | 52/219 |  |  |  |
| 1m2oA | 0.767 | 0.101 | 0.385 | 0.79 | 0.099 | 0.16 | 15 | 501 | 133 | 24 | 63/179 | 63/179 |  |  |  |
| 1dm5A | 0.761 | 0.368 | 0.108 | 0.947 | 0.093 | 0.167 | 7 | 216 | 12 | 58 | 13/224 | 13/224 |  |  |  |
| 1lm7A | 0.818 | 0.192 | 0.2 | 0.895 | 0.093 | 0.196 | 5 | 179 | 21 | 20 | 16/90 | 16/90 |  |  |  |
| 1qbkB | 0.612 | 0.102 | 0.55 | 0.617 | 0.089 | 0.171 | 33 | 470 | 292 | 27 | 103/235 | 57/133 | 46/102 |  |  |
| 1d3bB | 0.538 | 0.488 | 0.6 | 0.488 | 0.088 | 0.538 | 21 | 21 | 22 | 14 | 69/141 | 69/141 |  |  |  |
| 1g8eA | 0.589 | 0.591 | 0.945 | 0.1 | 0.086 | 0.727 | 52 | 4 | 36 | 3 | 221/252 | 221/252 |  |  |  |
| 1hcfX | 0.516 | 0.34 | 0.621 | 0.47 | 0.084 | 0.439 | 18 | 31 | 35 | 11 | 67/116 | 67/116 |  |  |  |
| 1jzdC | 0.607 | 0.316 | 0.4 | 0.683 | 0.078 | 0.353 | 12 | 56 | 26 | 18 | 46/103 | 46/68 | 0/35 |  |  |
| 1ev2E | 0.544 | 0.4 | 0.517 | 0.559 | 0.073 | 0.451 | 30 | 57 | 45 | 28 | 95/229 | 95/229 |  |  |  |
| 1xdtR | 0.595 | 0.606 | 0.909 | 0.133 | 0.067 | 0.727 | 20 | 2 | 13 | 2 | 77/95 | 77/95 |  |  |  |
| 1jmaB | 0.5 | 0.468 | 0.8 | 0.255 | 0.064 | 0.59 | 36 | 14 | 41 | 9 | 106/147 | 60/99 | 46/48 |  |  |
| 1h2iA | 0.615 | 0.728 | 0.734 | 0.32 | 0.054 | 0.731 | 91 | 16 | 34 | 33 | 372/595 | 372/595 |  |  |  |
| 1cjxA | 0.81 | 0.098 | 0.217 | 0.854 | 0.05 | 0.135 | 5 | 268 | 46 | 18 | 26/91 | 26/91 |  |  |  |
| 1zbdB | 0.517 | 0.356 | 0.525 | 0.513 | 0.036 | 0.424 | 21 | 40 | 38 | 19 | 80/188 | 80/188 |  |  |  |
| 1m1eB | 0.525 | 0.537 | 0.71 | 0.321 | 0.034 | 0.611 | 22 | 9 | 19 | 9 | 95/136 | 53/79 | 42/57 |  |  |
| 1ccwB | 0.788 | 0.088 | 0.2 | 0.835 | 0.025 | 0.122 | 6 | 314 | 62 | 24 | 21/124 | 21/124 |  |  |  |
| 1icwA | 0.485 | 0.375 | 0.6 | 0.419 | 0.018 | 0.462 | 15 | 18 | 25 | 10 | 62/114 | 62/114 |  |  |  |
| 1dqsA | 0.733 | 0.145 | 0.205 | 0.815 | 0.017 | 0.17 | 9 | 233 | 53 | 35 | 36/178 | 36/178 |  |  |  |
| 1ldjA | 0.787 | 0.109 | 0.164 | 0.854 | 0.015 | 0.131 | 11 | 527 | 90 | 56 | 49/290 | 49/290 |  |  |  |
| 1ebdC | 0.378 | 0.3 | 0.818 | 0.192 | 0.012 | 0.439 | 9 | 5 | 21 | 2 | 32/54 | 32/54 |  |  |  |
| 1aroP | 0.741 | 0.045 | 0.258 | 0.762 | 0.009 | 0.077 | 8 | 538 | 168 | 23 | 30/130 | 30/130 |  |  |  |
| 1ay7A | 0 | 0 | 0 | 0 | 0 | 0 | 0 | 73 | 0 | 18 | 0/69 | 0/69 |  |  |  |
| 1jb0K | 0 | 0 | 0 | 0 | 0 | 0 | 0 | 7 | 0 | 5 | 0/0 |  |  |  |  |
| 1mbxA | 0.566 | 0.18 | 0.375 | 0.61 | -0.012 | 0.243 | 9 | 64 | 41 | 15 | 39/102 | 39/102 |  |  |  |
| 1gvnB | 0.716 | 0.12 | 0.171 | 0.802 | -0.023 | 0.141 | 6 | 178 | 44 | 29 | 29/151 | 29/151 |  |  |  |
| 1euvB | 0.543 | 0.333 | 0.28 | 0.689 | -0.033 | 0.304 | 7 | 31 | 14 | 18 | 38/108 | 38/108 |  |  |  |
| 1lqsR | 0.579 | 0.108 | 0.32 | 0.616 | -0.044 | 0.162 | 8 | 106 | 66 | 17 | 28/95 | 28/95 |  |  |  |
| 1nmuA | 0.908 | 0 | 0 | 0.94 | -0.047 | 0 | 0 | 314 | 20 | 12 | 0/43 | 0/43 |  |  |  |
| 1gpwA | 0.747 | 0.091 | 0.091 | 0.853 | -0.056 | 0.091 | 3 | 174 | 30 | 30 | 4/131 | 4/131 |  |  |  |
| 1hc9A | 0.507 | 0.226 | 0.389 | 0.547 | -0.056 | 0.286 | 7 | 29 | 24 | 11 | 29/71 | 29/71 |  |  |  |
| 1h6kX | 0.471 | 0.394 | 0.448 | 0.487 | -0.064 | 0.419 | 13 | 19 | 20 | 16 | 46/134 | 46/134 |  |  |  |
| 1fskA | 0.636 | 0.091 | 0.2 | 0.701 | -0.073 | 0.125 | 4 | 94 | 40 | 16 | 13/82 | 13/82 |  |  |  |
| 1tiiC | 0.806 | 0.829 | 0.967 | 0 | -0.076 | 0.892 | 29 | 0 | 6 | 1 | 158/168 | 158/168 |  |  |  |
| 1go4E | 0.552 | 0.603 | 0.815 | 0.121 | -0.084 | 0.693 | 44 | 4 | 29 | 10 | 198/284 | 198/284 |  |  |  |
| 1kg0C | 0.364 | 0.104 | 0.5 | 0.343 | -0.111 | 0.172 | 8 | 36 | 69 | 8 | 20/61 | 20/61 |  |  |  |
| 1l6rA | 0.738 | 0.034 | 0.043 | 0.833 | -0.112 | 0.038 | 1 | 140 | 28 | 22 | 3/78 | 3/78 |  |  |  |
| 1ghqA | 0.762 | 0 | 0 | 0.816 | -0.12 | 0 | 0 | 199 | 45 | 17 | 0/79 | 0/79 |  |  |  |
| 1cseE | 0.778 | 0 | 0 | 0.893 | -0.123 | 0 | 0 | 175 | 21 | 29 | 0/91 | 0/91 |  |  |  |
| 1fp3A | 0.748 | 0 | 0 | 0.811 | -0.134 | 0 | 0 | 258 | 60 | 27 | 0/126 | 0/126 |  |  |  |
| 1qa9B | 0.444 | 0.133 | 0.353 | 0.466 | -0.142 | 0.194 | 6 | 34 | 39 | 11 | 13/70 | 13/70 |  |  |  |
| 1dx5I | 0.243 | 0.147 | 0.7 | 0.147 | -0.153 | 0.243 | 14 | 14 | 81 | 6 | 53/82 | 53/82 |  |  |  |
| 1g3jA | 0.587 | 0.087 | 0.123 | 0.696 | -0.16 | 0.102 | 10 | 240 | 105 | 71 | 25/279 | 25/279 |  |  |  |
| 1qa9A | 0.667 | 0 | 0 | 0.815 | -0.199 | 0 | 0 | 66 | 15 | 18 | 0/68 | 0/68 |  |  |  |
| 1d4vA | 0.391 | 0.21 | 0.382 | 0.395 | -0.204 | 0.271 | 13 | 32 | 49 | 21 | 52/138 | 52/138 |  |  |  |
| 1hqrD | 0.618 | 0 | 0 | 0.704 | -0.22 | 0 | 0 | 107 | 45 | 21 | 0/85 | 0/85 |  |  |  |
| 1g3jB | 0.576 | 0.864 | 0.633 | 0 | -0.224 | 0.731 | 19 | 0 | 3 | 11 | 106/177 | 106/177 |  |  |  |
| 1be3I | 0.969 | 0.969 | 1 | 0 | NaN | 0.984 | 31 | 0 | 1 | 0 | 155/156 | 155/156 |  |  |  |
| 1g2yA | 0.759 | 0.759 | 1 | 0 | NaN | 0.863 | 22 | 0 | 7 | 0 | 95/98 | 95/98 |  |  |  |
| 1jb0M | 0.7 | 0.7 | 1 | 0 | NaN | 0.824 | 21 | 0 | 9 | 0 | 94/94 | 94/94 |  |  |  |
| 1kveA | 0.754 | 0.754 | 1 | 0 | NaN | 0.86 | 46 | 0 | 15 | 0 | 225/229 | 225/229 |  |  |  |
| 1n13A | 0.911 | 0.911 | 1 | 0 | NaN | 0.953 | 41 | 0 | 4 | 0 | 219/228 | 219/228 |  |  |  |
| 1svfB | 0.73 | 0.73 | 1 | 0 | NaN | 0.844 | 27 | 0 | 10 | 0 | 123/123 | 123/123 |  |  |  |
| Total | 0.759 | 0.512 | 0.662 | 0.791 | 0.42 | 0.578 | 13970 | 50458 | 13300 | 7118 | - |  |  |  |  |

**Table S2:** Five-fold cross validation of ANN_BAGGING prediction accuracy benchmarks on the S432 dataset. The dataset, the 5-fold cross validation, and the benchmark measurements have been described in the main text. Matthews correlation coefficient (MCC), F-score(Fsc), Accuracy(Acc), Precision(Pre), Sensitivity(Sen) and Specificity(Spe) are shown in Equations (6)~(11) in the main text. TP, FP, TN, and FN are true positive, false positive, true negative, and false negative respectively. The ratio of the number of predicted positive atoms against actual number of binding atoms for each protein is also listed. C1~C4 represent PPI sites in each of the test proteins; different protein has different number of PPI sites. In these columns, the number of the predicted true positive atoms is shown over the actual number of atoms involving in the PPI site. Interactive examination of the prediction results for each of the proteins in the S432 dataset can be accessed from the web server: <http://ismblab.genomics.sinica.edu.tw/>> benchmark > protein-protein.
