# Supplementary material for: Protein-Protein Interaction Site Predictions with Three-Dimensional Probability Distributions of Interacting Atoms on Protein Surfaces
Source: PLoS One. 2012 Jun 6;7(6):e37706. doi: 10.1371/journal.pone.0037706 (PMC3368894; doi:10.1371/journal.pone.0037706)
Supplement: Table S3 — Five-fold cross validation of SVM_BAGGING prediction accuracy benchmarks on the S432 dataset. The dataset, the 5-fold cross validation, and the benchmark measurements have been described in the main text. Matthews correlation coefficient (MCC), F-score(Fsc), Accuracy(Acc), Precision(Pre), Sensitivity(Sen) and Specificity(Spe) are shown in Equations (6)∼(11) in the main text. TP, FP, TN, and FN are true positive, false positive, true negative, and false negative respectively. The ratio of the number of predicted positive atoms against actual number of binding atoms for each protein is also listed. C1∼C4 represent PPI sites in each of the test proteins; different protein has different number of PPI sites. In these columns, the number of the predicted true positive atoms is shown over the actual number of atoms involving in the PPI site. Interactive examination of the prediction results for each of the proteins in the S432 dataset can be accessed from the web server: http://ismblab.genomics.sinica.edu.tw/> benchmark >protein-protein. (DOCX) [file pone.0037706.s006.docx]

**Table S3. Residue-based cross validation benchmarks of SVM_BAGGING with S432 dataset.**

|  | Residue Level Benchmark | | | | | | | | | | Predict positive atoms / Actual binding atoms | | | | |
| --- | --- | --- | --- | --- | --- | --- | --- | --- | --- | --- | --- | --- | --- | --- | --- |
| PDBID | Acc | Pre | Sen | Spe | MCC | Fsc | TP | TN | FP | FN | All | C1 | C2 | C3 | C4 |
| 1jtdB | 0.95 | 0.77 | 0.84 | 0.96 | 0.777 | 0.81 | 27 | 212 | 8 | 5 | 87/124 | 87/124 |  |  |  |
| 1euaA | 0.93 | 0.84 | 0.77 | 0.97 | 0.76 | 0.8 | 26 | 150 | 5 | 8 | 77/116 | 77/116 |  |  |  |
| 1kwsA | 0.9 | 0.76 | 0.82 | 0.92 | 0.728 | 0.79 | 42 | 156 | 13 | 9 | 147/212 | 147/212 |  |  |  |
| 1bazA | 0.86 | 0.84 | 0.93 | 0.75 | 0.703 | 0.89 | 27 | 15 | 5 | 2 | 142/161 | 142/161 |  |  |  |
| 1n13B | 0.85 | 0.81 | 0.95 | 0.71 | 0.688 | 0.88 | 56 | 31 | 13 | 3 | 223/251 | 223/251 |  |  |  |
| 1e44B | 0.88 | 0.89 | 0.64 | 0.97 | 0.682 | 0.74 | 16 | 63 | 2 | 9 | 72/129 | 72/129 |  |  |  |
| 1hynP | 0.86 | 0.6 | 0.93 | 0.84 | 0.664 | 0.73 | 50 | 181 | 34 | 4 | 209/244 | 209/244 |  |  |  |
| 1k8kD | 0.86 | 0.8 | 0.73 | 0.92 | 0.663 | 0.76 | 58 | 172 | 15 | 22 | 252/364 | 252/364 |  |  |  |
| 1jmvA | 0.85 | 0.6 | 0.93 | 0.82 | 0.654 | 0.73 | 25 | 79 | 17 | 2 | 98/120 | 98/120 |  |  |  |
| 1n1jB | 0.83 | 0.8 | 0.96 | 0.65 | 0.651 | 0.87 | 43 | 20 | 11 | 2 | 198/216 | 198/216 |  |  |  |
| 1b16A | 0.84 | 0.65 | 0.86 | 0.84 | 0.644 | 0.74 | 51 | 142 | 28 | 8 | 184/245 | 184/245 |  |  |  |
| 1apyB | 0.83 | 0.87 | 0.87 | 0.77 | 0.637 | 0.87 | 71 | 37 | 11 | 11 | 297/383 | 297/383 |  |  |  |
| 1jjuB | 0.9 | 0.69 | 0.71 | 0.94 | 0.637 | 0.7 | 37 | 246 | 17 | 15 | 124/202 | 124/202 |  |  |  |
| 1ffgB | 0.86 | 0.71 | 0.75 | 0.89 | 0.631 | 0.73 | 12 | 42 | 5 | 4 | 50/65 | 50/65 |  |  |  |
| 12asA | 0.87 | 0.56 | 0.88 | 0.87 | 0.63 | 0.68 | 42 | 218 | 33 | 6 | 136/186 | 136/186 |  |  |  |
| 1ig0A | 0.89 | 0.59 | 0.81 | 0.91 | 0.628 | 0.68 | 33 | 221 | 23 | 8 | 106/161 | 106/161 |  |  |  |
| 1c28A | 0.81 | 0.76 | 0.83 | 0.79 | 0.622 | 0.79 | 40 | 50 | 13 | 8 | 144/216 | 144/216 |  |  |  |
| 1k8kG | 0.83 | 0.77 | 0.73 | 0.88 | 0.621 | 0.75 | 33 | 74 | 10 | 12 | 149/213 | 149/213 |  |  |  |
| 1eaiC | 0.82 | 0.88 | 0.63 | 0.94 | 0.619 | 0.73 | 15 | 34 | 2 | 9 | 64/93 | 64/93 |  |  |  |
| 1k8kF | 0.79 | 0.95 | 0.72 | 0.93 | 0.618 | 0.82 | 76 | 51 | 4 | 29 | 330/517 | 330/517 |  |  |  |
| 1k28A | 0.81 | 0.85 | 0.84 | 0.78 | 0.617 | 0.84 | 267 | 175 | 49 | 52 | 1436/1759 | 1436/1759 | |  |  |
| 1mg2A | 0.84 | 0.71 | 0.75 | 0.87 | 0.614 | 0.73 | 76 | 210 | 31 | 25 | 280/422 | 280/422 |  |  |  |
| 1ep3B | 0.9 | 0.68 | 0.66 | 0.95 | 0.61 | 0.67 | 23 | 192 | 11 | 12 | 87/138 | 87/138 |  |  |  |
| 1bzyA | 0.84 | 0.85 | 0.58 | 0.96 | 0.604 | 0.69 | 34 | 128 | 6 | 25 | 111/264 | 111/264 |  |  |  |
| 1jeqA | 0.79 | 0.7 | 0.87 | 0.74 | 0.597 | 0.77 | 184 | 227 | 80 | 28 | 769/1006 | 738/949 | 31/57 |  |  |
| 1b8gA | 0.87 | 0.59 | 0.76 | 0.9 | 0.593 | 0.66 | 50 | 300 | 35 | 16 | 196/278 | 196/278 |  |  |  |
| 1fo0A | 0.78 | 0.57 | 0.94 | 0.72 | 0.593 | 0.71 | 29 | 57 | 22 | 2 | 99/131 | 99/131 |  |  |  |
| 1f8mA | 0.79 | 0.85 | 0.68 | 0.89 | 0.592 | 0.76 | 129 | 191 | 23 | 60 | 504/805 | 504/805 |  |  |  |
| 1h59B | 0.79 | 0.68 | 0.88 | 0.72 | 0.592 | 0.77 | 15 | 18 | 7 | 2 | 57/72 | 57/72 |  |  |  |
| 1g57A | 0.87 | 0.66 | 0.69 | 0.91 | 0.591 | 0.68 | 27 | 141 | 14 | 12 | 87/146 | 87/146 |  |  |  |
| 1bouA | 0.79 | 0.74 | 0.85 | 0.75 | 0.59 | 0.79 | 49 | 50 | 17 | 9 | 195/245 | 195/245 |  |  |  |
| 1m6pA | 0.87 | 0.72 | 0.62 | 0.94 | 0.59 | 0.67 | 18 | 104 | 7 | 11 | 55/109 | 55/109 |  |  |  |
| 1j7dA | 0.82 | 0.43 | 1 | 0.8 | 0.581 | 0.6 | 17 | 89 | 23 | 0 | 61/79 | 61/79 |  |  |  |
| 1gpqA | 0.81 | 0.72 | 0.72 | 0.86 | 0.579 | 0.72 | 28 | 68 | 11 | 11 | 98/180 | 98/180 |  |  |  |
| 1ax4A | 0.85 | 0.67 | 0.67 | 0.9 | 0.578 | 0.67 | 62 | 283 | 30 | 30 | 246/416 | 246/416 |  |  |  |
| 1lktA | 0.79 | 0.81 | 0.84 | 0.74 | 0.578 | 0.82 | 46 | 31 | 11 | 9 | 165/213 | 165/213 |  |  |  |
| 1jeqB | 0.78 | 0.7 | 0.85 | 0.74 | 0.577 | 0.76 | 175 | 217 | 76 | 32 | 774/987 | 774/932 | 0/55 |  |  |
| 1hq3A | 0.82 | 0.84 | 0.91 | 0.63 | 0.574 | 0.88 | 64 | 20 | 12 | 6 | 274/321 | 274/321 |  |  |  |
| 1aihA | 0.77 | 0.6 | 0.9 | 0.72 | 0.572 | 0.72 | 44 | 76 | 30 | 5 | 175/208 | 175/208 |  |  |  |
| 1gg2B | 0.8 | 0.69 | 0.77 | 0.81 | 0.569 | 0.73 | 81 | 159 | 37 | 24 | 230/365 | 230/365 |  |  |  |
| 1e44A | 0.76 | 0.64 | 0.9 | 0.67 | 0.565 | 0.75 | 28 | 33 | 16 | 3 | 105/146 | 105/146 |  |  |  |
| 1go3F | 0.77 | 0.7 | 0.85 | 0.71 | 0.563 | 0.77 | 35 | 37 | 15 | 6 | 147/200 | 147/200 |  |  |  |
| 1lm8B | 0.75 | 0.52 | 0.96 | 0.67 | 0.563 | 0.68 | 26 | 49 | 24 | 1 | 105/119 | 105/119 |  |  |  |
| 1dmlB | 0.86 | 0.84 | 1 | 0.38 | 0.562 | 0.92 | 27 | 3 | 5 | 0 | 113/125 | 113/125 |  |  |  |
| 1dcuA | 0.82 | 0.69 | 0.69 | 0.87 | 0.557 | 0.69 | 57 | 175 | 26 | 26 | 211/342 | 211/342 |  |  |  |
| 1h1yA | 0.88 | 0.58 | 0.68 | 0.92 | 0.557 | 0.62 | 19 | 154 | 14 | 9 | 61/100 | 61/100 |  |  |  |
| 1e2aA | 0.77 | 0.7 | 0.89 | 0.66 | 0.556 | 0.78 | 39 | 33 | 17 | 5 | 159/198 | 159/198 |  |  |  |
| 1m2dA | 0.76 | 0.44 | 1 | 0.71 | 0.555 | 0.61 | 17 | 53 | 22 | 0 | 57/62 | 57/62 |  |  |  |
| 1f8uB | 0.78 | 0.76 | 0.83 | 0.71 | 0.553 | 0.79 | 25 | 20 | 8 | 5 | 102/146 | 102/146 |  |  |  |
| 1d2zA | 0.79 | 0.57 | 0.84 | 0.77 | 0.552 | 0.68 | 21 | 54 | 16 | 4 | 52/104 | 52/104 |  |  |  |
| 1kb9F | 0.78 | 0.74 | 0.74 | 0.81 | 0.551 | 0.74 | 23 | 34 | 8 | 8 | 81/146 | 81/146 |  |  |  |
| 1j5sA | 0.84 | 0.66 | 0.65 | 0.9 | 0.55 | 0.65 | 62 | 288 | 32 | 34 | 239/434 | 239/434 |  |  |  |
| 1bykA | 0.82 | 0.48 | 0.86 | 0.82 | 0.549 | 0.61 | 31 | 152 | 34 | 5 | 106/145 | 106/145 |  |  |  |
| 1k8kE | 0.86 | 0.42 | 0.89 | 0.85 | 0.547 | 0.57 | 16 | 125 | 22 | 2 | 80/94 | 80/94 |  |  |  |
| 1ik9A | 0.72 | 0.53 | 0.98 | 0.59 | 0.544 | 0.69 | 63 | 83 | 57 | 1 | 279/312 | 279/312 |  |  |  |
| 1poiB | 0.81 | 0.65 | 0.71 | 0.85 | 0.544 | 0.68 | 48 | 149 | 26 | 20 | 156/279 | 156/279 |  |  |  |
| 1g0sA | 0.77 | 0.72 | 0.8 | 0.74 | 0.542 | 0.76 | 69 | 78 | 27 | 17 | 282/397 | 282/397 |  |  |  |
| 1rveA | 0.87 | 0.58 | 0.66 | 0.91 | 0.54 | 0.62 | 25 | 180 | 18 | 13 | 86/160 | 86/146 | 0/14 |  |  |
| 1ltsC | 0.93 | 0.95 | 0.97 | 0.5 | 0.539 | 0.96 | 36 | 2 | 2 | 1 | 190/227 | 190/227 |  |  |  |
| 1j79A | 0.91 | 0.51 | 0.68 | 0.93 | 0.538 | 0.58 | 21 | 265 | 20 | 10 | 72/118 | 72/118 |  |  |  |
| 1l2wA | 0.77 | 0.76 | 0.82 | 0.71 | 0.536 | 0.79 | 51 | 39 | 16 | 11 | 204/263 | 204/263 |  |  |  |
| 1a0fA | 0.83 | 0.61 | 0.68 | 0.87 | 0.534 | 0.64 | 28 | 123 | 18 | 13 | 94/172 | 94/172 |  |  |  |
| 1ireA | 0.75 | 0.89 | 0.58 | 0.92 | 0.534 | 0.7 | 55 | 85 | 7 | 40 | 233/440 | 233/440 |  |  |  |
| 1g99A | 0.84 | 0.59 | 0.69 | 0.88 | 0.532 | 0.63 | 50 | 250 | 35 | 23 | 199/315 | 199/315 |  |  |  |
| 1cydA | 0.79 | 0.71 | 0.67 | 0.85 | 0.529 | 0.69 | 52 | 123 | 21 | 26 | 222/341 | 222/341 |  |  |  |
| 1ed9A | 0.84 | 0.76 | 0.5 | 0.95 | 0.528 | 0.6 | 48 | 288 | 15 | 48 | 154/405 | 154/405 |  |  |  |
| 1l1oA | 0.74 | 0.55 | 0.91 | 0.66 | 0.528 | 0.68 | 30 | 49 | 25 | 3 | 121/151 | 121/151 |  |  |  |
| 1ynjA | 0.77 | 0.72 | 0.76 | 0.78 | 0.528 | 0.74 | 68 | 93 | 27 | 22 | 259/390 | 259/390 |  |  |  |
| 1gcqC | 0.73 | 0.64 | 0.93 | 0.57 | 0.527 | 0.76 | 27 | 20 | 15 | 2 | 83/111 | 83/111 |  |  |  |
| 1dkgA | 0.75 | 0.7 | 0.88 | 0.63 | 0.525 | 0.78 | 65 | 48 | 28 | 9 | 248/309 | 248/309 |  |  |  |
| 1nvtA | 0.81 | 0.44 | 0.87 | 0.8 | 0.524 | 0.58 | 34 | 177 | 44 | 5 | 113/146 | 113/146 |  |  |  |
| 1jyoA | 0.76 | 0.82 | 0.75 | 0.78 | 0.523 | 0.78 | 54 | 42 | 12 | 18 | 184/280 | 184/280 |  |  |  |
| 1e9gA | 0.91 | 0.48 | 0.67 | 0.94 | 0.522 | 0.56 | 14 | 220 | 15 | 7 | 70/112 | 70/112 |  |  |  |
| 1i1rB | 0.73 | 0.47 | 0.95 | 0.66 | 0.521 | 0.63 | 35 | 76 | 39 | 2 | 155/165 | 63/71 | 92/94 |  |  |
| 1l1oC | 0.78 | 0.44 | 0.9 | 0.76 | 0.521 | 0.59 | 26 | 105 | 33 | 3 | 119/144 | 119/144 |  |  |  |
| 1ei1A | 0.85 | 0.66 | 0.56 | 0.93 | 0.519 | 0.61 | 40 | 262 | 21 | 31 | 150/281 | 117/242 | 33/39 |  |  |
| 1li1A | 0.77 | 0.79 | 0.86 | 0.65 | 0.519 | 0.82 | 108 | 53 | 29 | 18 | 415/553 | 415/553 |  |  |  |
| 1fwxA | 0.81 | 0.74 | 0.55 | 0.92 | 0.518 | 0.64 | 87 | 333 | 30 | 70 | 332/665 | 332/665 |  |  |  |
| 1l1oB | 0.76 | 0.67 | 0.78 | 0.75 | 0.518 | 0.72 | 36 | 53 | 18 | 10 | 138/201 | 138/201 |  |  |  |
| 1hn2A | 0.74 | 0.58 | 0.87 | 0.68 | 0.515 | 0.69 | 45 | 70 | 33 | 7 | 194/244 | 194/244 |  |  |  |
| 1jkeA | 0.78 | 0.63 | 0.73 | 0.8 | 0.515 | 0.67 | 30 | 74 | 18 | 11 | 117/184 | 117/184 |  |  |  |
| 1keyA | 0.76 | 0.69 | 0.73 | 0.79 | 0.51 | 0.71 | 56 | 92 | 25 | 21 | 206/332 | 206/332 |  |  |  |
| 1g0hA | 0.86 | 0.52 | 0.67 | 0.89 | 0.509 | 0.59 | 22 | 167 | 20 | 11 | 80/131 | 80/131 |  |  |  |
| 1el6A | 0.75 | 0.71 | 0.85 | 0.64 | 0.505 | 0.78 | 87 | 62 | 35 | 15 | 307/417 | 307/417 |  |  |  |
| 1f3vA | 0.87 | 0.5 | 0.67 | 0.9 | 0.505 | 0.57 | 12 | 110 | 12 | 6 | 43/79 | 43/79 |  |  |  |
| 1fuiA | 0.84 | 0.55 | 0.66 | 0.88 | 0.502 | 0.6 | 65 | 386 | 53 | 34 | 230/416 | 230/416 |  |  |  |
| 1i0dA | 0.84 | 0.4 | 0.83 | 0.84 | 0.501 | 0.54 | 29 | 223 | 43 | 6 | 96/152 | 96/152 |  |  |  |
| 1d7aA | 0.76 | 0.83 | 0.77 | 0.74 | 0.499 | 0.8 | 68 | 39 | 14 | 20 | 254/390 | 254/390 |  |  |  |
| 1at3A | 0.8 | 0.41 | 0.86 | 0.79 | 0.498 | 0.56 | 24 | 127 | 34 | 4 | 90/118 | 90/118 |  |  |  |
| 1f80A | 0.75 | 0.69 | 0.78 | 0.72 | 0.498 | 0.73 | 35 | 42 | 16 | 10 | 127/202 | 127/202 |  |  |  |
| 1fcdA | 0.85 | 0.42 | 0.78 | 0.86 | 0.498 | 0.54 | 32 | 283 | 45 | 9 | 114/178 | 114/178 |  |  |  |
| 1fuxA | 0.8 | 0.4 | 0.86 | 0.79 | 0.497 | 0.55 | 19 | 106 | 28 | 3 | 57/90 | 57/90 |  |  |  |
| 1a2xA | 0.78 | 0.65 | 0.66 | 0.84 | 0.496 | 0.65 | 31 | 89 | 17 | 16 | 102/193 | 102/193 |  |  |  |
| 1smtA | 0.71 | 0.61 | 0.93 | 0.55 | 0.496 | 0.74 | 39 | 30 | 25 | 3 | 167/200 | 167/200 |  |  |  |
| 1kpsB | 0.79 | 0.46 | 0.82 | 0.78 | 0.492 | 0.59 | 22 | 92 | 26 | 5 | 103/134 | 103/134 |  |  |  |
| 1hg3A | 0.8 | 0.67 | 0.58 | 0.89 | 0.489 | 0.62 | 32 | 125 | 16 | 23 | 113/220 | 113/220 |  |  |  |
| 3fivA | 0.74 | 0.67 | 0.8 | 0.7 | 0.489 | 0.73 | 35 | 39 | 17 | 9 | 143/185 | 143/185 |  |  |  |
| 1gpwB | 0.86 | 0.5 | 0.64 | 0.9 | 0.488 | 0.56 | 16 | 143 | 16 | 9 | 75/117 | 75/117 |  |  |  |
| 1h21A | 0.73 | 0.65 | 0.86 | 0.63 | 0.488 | 0.74 | 83 | 76 | 45 | 14 | 314/445 | 314/445 |  |  |  |
| 1icfI | 0.66 | 0.5 | 1 | 0.48 | 0.488 | 0.67 | 22 | 20 | 22 | 0 | 87/104 | 87/104 |  |  |  |
| 1adjA | 0.81 | 0.61 | 0.62 | 0.87 | 0.487 | 0.61 | 57 | 249 | 37 | 35 | 225/401 | 225/401 |  |  |  |
| 1aonO | 0.74 | 0.72 | 0.81 | 0.67 | 0.487 | 0.76 | 38 | 31 | 15 | 9 | 140/195 | 39/40 | 101/155 |  |  |
| 1fm0D | 0.73 | 0.51 | 0.86 | 0.67 | 0.485 | 0.64 | 19 | 37 | 18 | 3 | 70/85 | 70/85 |  |  |  |
| 1k83K | 0.76 | 0.84 | 0.79 | 0.71 | 0.484 | 0.81 | 59 | 27 | 11 | 16 | 240/340 | 240/340 |  |  |  |
| 1bjnA | 0.82 | 0.51 | 0.69 | 0.85 | 0.483 | 0.58 | 42 | 235 | 41 | 19 | 123/222 | 123/222 |  |  |  |
| 1f3uB | 0.67 | 0.53 | 0.98 | 0.48 | 0.483 | 0.69 | 50 | 42 | 45 | 1 | 229/245 | 229/245 |  |  |  |
| 1k3bA | 0.74 | 0.69 | 0.78 | 0.7 | 0.483 | 0.73 | 40 | 42 | 18 | 11 | 151/219 | 151/219 |  |  |  |
| 1k83H | 0.76 | 0.6 | 0.73 | 0.77 | 0.482 | 0.66 | 30 | 68 | 20 | 11 | 123/201 | 123/201 |  |  |  |
| 1l6xB | 0.74 | 0.68 | 0.81 | 0.67 | 0.482 | 0.74 | 13 | 12 | 6 | 3 | 60/77 | 60/77 |  |  |  |
| 2eboA | 0.78 | 0.76 | 1 | 0.3 | 0.48 | 0.86 | 50 | 7 | 16 | 0 | 214/249 | 214/249 |  |  |  |
| 1k75A | 0.75 | 0.59 | 0.75 | 0.75 | 0.478 | 0.66 | 94 | 200 | 66 | 31 | 361/539 | 361/539 |  |  |  |
| 1tdtA | 0.75 | 0.56 | 0.79 | 0.73 | 0.478 | 0.66 | 56 | 116 | 44 | 15 | 224/312 | 224/312 |  |  |  |
| 1ixmA | 0.71 | 0.47 | 0.89 | 0.65 | 0.476 | 0.62 | 25 | 52 | 28 | 3 | 111/128 | 111/128 |  |  |  |
| 1ad3A | 0.8 | 0.62 | 0.6 | 0.87 | 0.475 | 0.61 | 62 | 260 | 38 | 42 | 248/408 | 248/408 |  |  |  |
| 1jtgB | 0.75 | 0.45 | 0.85 | 0.72 | 0.475 | 0.59 | 28 | 87 | 34 | 5 | 119/153 | 119/153 |  |  |  |
| 1ewyC | 0.76 | 0.66 | 0.68 | 0.8 | 0.474 | 0.67 | 23 | 48 | 12 | 11 | 80/137 | 80/137 |  |  |  |
| 1lqpA | 0.73 | 0.69 | 0.85 | 0.62 | 0.474 | 0.76 | 55 | 40 | 25 | 10 | 224/276 | 224/276 |  |  |  |
| 1udiI | 0.74 | 0.62 | 0.75 | 0.74 | 0.474 | 0.68 | 21 | 37 | 13 | 7 | 74/109 | 74/109 |  |  |  |
| 1jw9B | 0.73 | 0.66 | 0.77 | 0.7 | 0.47 | 0.71 | 71 | 88 | 37 | 21 | 277/396 | 277/396 |  |  |  |
| 1a88A | 0.85 | 0.52 | 0.59 | 0.9 | 0.468 | 0.55 | 23 | 191 | 21 | 16 | 84/163 | 84/163 |  |  |  |
| 1e5xA | 0.76 | 0.5 | 0.78 | 0.75 | 0.468 | 0.61 | 77 | 238 | 78 | 22 | 283/415 | 283/415 |  |  |  |
| 1fcjA | 0.84 | 0.51 | 0.62 | 0.89 | 0.468 | 0.56 | 26 | 195 | 25 | 16 | 93/188 | 93/188 |  |  |  |
| 1ld8A | 0.79 | 0.67 | 0.55 | 0.89 | 0.466 | 0.6 | 46 | 185 | 23 | 38 | 201/367 | 201/367 |  |  |  |
| 1lk5A | 0.78 | 0.64 | 0.61 | 0.85 | 0.465 | 0.62 | 37 | 121 | 21 | 24 | 125/235 | 125/235 |  |  |  |
| 1tyfA | 0.73 | 0.74 | 0.74 | 0.72 | 0.464 | 0.74 | 67 | 59 | 23 | 23 | 282/422 | 282/422 |  |  |  |
| 1kkmA | 0.73 | 0.74 | 0.72 | 0.75 | 0.463 | 0.73 | 58 | 59 | 20 | 23 | 228/351 | 228/351 |  |  |  |
| 1id1A | 0.75 | 0.55 | 0.76 | 0.74 | 0.462 | 0.64 | 31 | 71 | 25 | 10 | 113/162 | 113/162 |  |  |  |
| 1ajsA | 0.8 | 0.56 | 0.63 | 0.85 | 0.461 | 0.6 | 57 | 241 | 44 | 33 | 204/345 | 204/345 |  |  |  |
| 1bbhA | 0.7 | 0.37 | 0.96 | 0.64 | 0.46 | 0.53 | 21 | 65 | 36 | 1 | 81/86 | 81/86 |  |  |  |
| 1avqA | 0.74 | 0.34 | 0.93 | 0.71 | 0.459 | 0.5 | 28 | 133 | 54 | 2 | 100/127 | 45/62 | 55/65 |  |  |
| 1g8mA | 0.78 | 0.52 | 0.7 | 0.8 | 0.459 | 0.6 | 88 | 317 | 80 | 37 | 354/573 | 354/573 |  |  |  |
| 1fm0E | 0.73 | 0.68 | 0.72 | 0.74 | 0.458 | 0.7 | 42 | 56 | 20 | 16 | 161/241 | 161/241 |  |  |  |
| 1m4uA | 0.69 | 0.52 | 0.89 | 0.59 | 0.457 | 0.66 | 59 | 78 | 55 | 7 | 245/294 | 143/158 | 102/136 |  |  |
| 1nrjA | 0.78 | 0.43 | 0.78 | 0.78 | 0.456 | 0.55 | 18 | 83 | 24 | 5 | 77/113 | 77/113 |  |  |  |
| 1a79A | 0.73 | 0.66 | 0.72 | 0.74 | 0.453 | 0.69 | 46 | 68 | 24 | 18 | 186/272 | 186/272 |  |  |  |
| 1byfA | 0.77 | 0.47 | 0.74 | 0.78 | 0.451 | 0.58 | 17 | 68 | 19 | 6 | 58/91 | 58/91 |  |  |  |
| 1gvnA | 0.7 | 0.9 | 0.64 | 0.85 | 0.448 | 0.75 | 37 | 22 | 4 | 21 | 148/264 | 148/264 |  |  |  |
| 1jqlB | 0.84 | 0.48 | 0.62 | 0.88 | 0.448 | 0.54 | 13 | 98 | 14 | 8 | 52/91 | 52/91 |  |  |  |
| 1n0wA | 0.71 | 0.35 | 0.93 | 0.67 | 0.445 | 0.51 | 28 | 108 | 53 | 2 | 106/128 | 106/128 |  |  |  |
| 1qo0D | 0.71 | 0.61 | 0.83 | 0.62 | 0.445 | 0.7 | 58 | 60 | 37 | 12 | 227/316 | 227/316 |  |  |  |
| 1dtwA | 0.73 | 0.71 | 0.66 | 0.78 | 0.444 | 0.68 | 101 | 149 | 42 | 52 | 387/659 | 387/659 |  |  |  |
| 1h32A | 0.78 | 0.32 | 0.89 | 0.77 | 0.442 | 0.47 | 24 | 170 | 52 | 3 | 80/124 | 80/124 |  |  |  |
| 1ynjK | 0.74 | 0.74 | 0.86 | 0.56 | 0.441 | 0.79 | 42 | 19 | 15 | 7 | 172/218 | 172/218 |  |  |  |
| 1lm8C | 0.73 | 0.72 | 0.83 | 0.6 | 0.44 | 0.77 | 39 | 22 | 15 | 8 | 175/224 | 175/224 |  |  |  |
| 1h32B | 0.72 | 0.47 | 0.82 | 0.68 | 0.439 | 0.59 | 27 | 67 | 31 | 6 | 104/141 | 104/141 |  |  |  |
| 1lh0A | 0.77 | 0.46 | 0.73 | 0.78 | 0.437 | 0.56 | 29 | 121 | 34 | 11 | 101/167 | 101/167 |  |  |  |
| 1ihrA | 0.86 | 0.89 | 0.95 | 0.42 | 0.436 | 0.92 | 58 | 5 | 7 | 3 | 289/333 | 289/333 |  |  |  |
| 1e7lA | 0.72 | 0.66 | 0.73 | 0.71 | 0.434 | 0.7 | 49 | 60 | 25 | 18 | 229/336 | 229/336 |  |  |  |
| 1h0hB | 0.77 | 0.52 | 0.68 | 0.79 | 0.434 | 0.59 | 34 | 122 | 32 | 16 | 117/208 | 117/208 |  |  |  |
| 1mr1C | 0.68 | 0.36 | 0.94 | 0.62 | 0.433 | 0.52 | 16 | 47 | 29 | 1 | 75/89 | 75/89 |  |  |  |
| 1nksA | 0.75 | 0.44 | 0.76 | 0.75 | 0.433 | 0.56 | 28 | 107 | 35 | 9 | 104/152 | 104/152 |  |  |  |
| 1ftrA | 0.73 | 0.68 | 0.6 | 0.82 | 0.429 | 0.64 | 61 | 132 | 29 | 41 | 205/410 | 205/410 |  |  |  |
| 1l9wA | 0.79 | 0.3 | 0.87 | 0.78 | 0.428 | 0.44 | 20 | 168 | 47 | 3 | 80/93 | 80/93 |  |  |  |
| 1chmA | 0.79 | 0.54 | 0.59 | 0.85 | 0.425 | 0.56 | 49 | 234 | 42 | 34 | 175/364 | 175/364 |  |  |  |
| 1kq4A | 0.69 | 0.58 | 0.84 | 0.59 | 0.424 | 0.69 | 63 | 65 | 46 | 12 | 251/327 | 251/327 |  |  |  |
| 1l0oA | 0.74 | 0.67 | 0.58 | 0.83 | 0.424 | 0.62 | 29 | 68 | 14 | 21 | 100/196 | 100/196 |  |  |  |
| 1hx3A | 0.79 | 0.36 | 0.77 | 0.79 | 0.423 | 0.49 | 17 | 112 | 30 | 5 | 66/87 | 66/87 |  |  |  |
| 1i9bA | 0.76 | 0.65 | 0.53 | 0.87 | 0.423 | 0.59 | 34 | 117 | 18 | 30 | 116/265 | 116/265 |  |  |  |
| 1azzC | 0.71 | 0.77 | 0.69 | 0.74 | 0.42 | 0.72 | 46 | 39 | 14 | 21 | 189/288 | 189/288 |  |  |  |
| 1n9rA | 0.7 | 0.65 | 0.83 | 0.58 | 0.42 | 0.73 | 24 | 18 | 13 | 5 | 101/134 | 101/134 |  |  |  |
| 1nbaA | 0.7 | 0.77 | 0.6 | 0.81 | 0.42 | 0.67 | 69 | 90 | 21 | 46 | 257/517 | 257/517 |  |  |  |
| 1d9eA | 0.75 | 0.68 | 0.52 | 0.87 | 0.419 | 0.59 | 40 | 126 | 19 | 37 | 169/330 | 169/330 |  |  |  |
| 1jjuA | 0.78 | 0.46 | 0.67 | 0.81 | 0.419 | 0.54 | 60 | 311 | 72 | 30 | 227/369 | 227/369 |  |  |  |
| 1n0wB | 0.76 | 0.77 | 0.91 | 0.46 | 0.419 | 0.83 | 20 | 5 | 6 | 2 | 84/93 | 84/93 |  |  |  |
| 1ld8B | 0.73 | 0.49 | 0.73 | 0.74 | 0.417 | 0.58 | 65 | 192 | 69 | 24 | 212/374 | 212/374 |  |  |  |
| 1lj2A | 0.75 | 0.76 | 0.91 | 0.44 | 0.415 | 0.83 | 62 | 16 | 20 | 6 | 305/350 | 305/350 |  |  |  |
| 1hcfX | 0.68 | 0.49 | 0.83 | 0.62 | 0.414 | 0.62 | 24 | 41 | 25 | 5 | 82/116 | 82/116 |  |  |  |
| 1ldjA | 0.83 | 0.33 | 0.73 | 0.84 | 0.414 | 0.46 | 49 | 519 | 98 | 18 | 176/290 | 176/290 |  |  |  |
| 1dbqA | 0.75 | 0.37 | 0.79 | 0.75 | 0.412 | 0.5 | 30 | 153 | 52 | 8 | 106/168 | 106/168 |  |  |  |
| 1diqC | 0.62 | 0.41 | 0.95 | 0.5 | 0.412 | 0.58 | 19 | 27 | 27 | 1 | 82/97 | 82/97 |  |  |  |
| 1miuB | 0.87 | 0.97 | 0.89 | 0.67 | 0.41 | 0.93 | 32 | 2 | 1 | 4 | 183/236 | 183/236 |  |  |  |
| 1f75A | 0.74 | 0.37 | 0.8 | 0.72 | 0.409 | 0.51 | 28 | 123 | 47 | 7 | 131/182 | 131/182 |  |  |  |
| 1jzdA | 0.73 | 0.36 | 0.82 | 0.71 | 0.409 | 0.5 | 28 | 123 | 50 | 6 | 99/134 | 80/98 | 19/36 |  |  |
| 1b33A | 0.69 | 0.52 | 0.8 | 0.63 | 0.406 | 0.63 | 40 | 63 | 37 | 10 | 157/205 | 157/205 |  |  |  |
| 1jyoE | 0.76 | 0.74 | 0.99 | 0.27 | 0.406 | 0.85 | 68 | 9 | 24 | 1 | 314/332 | 314/332 |  |  |  |
| 1jg5A | 0.7 | 0.69 | 0.79 | 0.61 | 0.404 | 0.74 | 34 | 23 | 15 | 9 | 136/192 | 136/192 |  |  |  |
| 1dbfA | 0.71 | 0.67 | 0.64 | 0.77 | 0.403 | 0.65 | 33 | 52 | 16 | 19 | 136/212 | 136/212 |  |  |  |
| 1eteA | 0.73 | 0.33 | 0.84 | 0.71 | 0.403 | 0.48 | 16 | 77 | 32 | 3 | 65/91 | 65/91 |  |  |  |
| 1l6wA | 0.7 | 0.68 | 0.66 | 0.74 | 0.401 | 0.67 | 58 | 77 | 27 | 30 | 243/382 | 243/382 |  |  |  |
| 1gl4A | 0.79 | 0.29 | 0.81 | 0.79 | 0.398 | 0.43 | 21 | 190 | 51 | 5 | 82/114 | 82/114 |  |  |  |
| 1tx4A | 0.8 | 0.42 | 0.63 | 0.83 | 0.398 | 0.51 | 19 | 126 | 26 | 11 | 68/113 | 68/113 |  |  |  |
| 3ygsP | 0.74 | 0.83 | 0.32 | 0.96 | 0.398 | 0.47 | 10 | 54 | 2 | 21 | 46/152 | 46/152 |  |  |  |
| 1hzpA | 0.74 | 0.55 | 0.63 | 0.79 | 0.397 | 0.58 | 52 | 157 | 43 | 31 | 200/330 | 200/330 |  |  |  |
| 1ia9A | 0.69 | 0.49 | 0.79 | 0.65 | 0.397 | 0.61 | 62 | 117 | 64 | 17 | 241/362 | 241/362 |  |  |  |
| 1isiA | 0.84 | 0.39 | 0.61 | 0.87 | 0.395 | 0.47 | 17 | 177 | 27 | 11 | 74/131 | 69/87 | 5/44 |  |  |
| 1g72A | 0.83 | 0.59 | 0.4 | 0.94 | 0.394 | 0.48 | 38 | 379 | 26 | 57 | 105/343 | 41/198 | 64/145 |  |  |
| 1gz0A | 0.77 | 0.35 | 0.75 | 0.77 | 0.394 | 0.48 | 24 | 145 | 44 | 8 | 79/131 | 79/131 |  |  |  |
| 1i2mB | 0.83 | 0.7 | 0.32 | 0.96 | 0.393 | 0.44 | 23 | 256 | 10 | 48 | 70/272 | 70/272 |  |  |  |
| 1bh9B | 0.61 | 0.52 | 0.97 | 0.36 | 0.392 | 0.67 | 34 | 18 | 32 | 1 | 134/147 | 134/147 |  |  |  |
| 1luaA | 0.82 | 0.36 | 0.65 | 0.85 | 0.392 | 0.47 | 20 | 196 | 35 | 11 | 80/117 | 80/117 |  |  |  |
| 1f34B | 0.68 | 0.47 | 0.8 | 0.64 | 0.39 | 0.59 | 31 | 61 | 35 | 8 | 111/157 | 111/157 |  |  |  |
| 1k83C | 0.69 | 0.7 | 0.69 | 0.7 | 0.389 | 0.7 | 88 | 87 | 37 | 40 | 369/580 | 369/580 |  |  |  |
| 1jqlA | 0.85 | 0.31 | 0.68 | 0.87 | 0.386 | 0.42 | 19 | 275 | 43 | 9 | 68/109 | 68/109 |  |  |  |
| 1fcdC | 0.71 | 0.38 | 0.79 | 0.69 | 0.385 | 0.52 | 26 | 92 | 42 | 7 | 100/138 | 100/138 |  |  |  |
| 1jb0D | 0.69 | 0.74 | 0.68 | 0.71 | 0.385 | 0.71 | 49 | 41 | 17 | 23 | 181/306 | 181/306 |  |  |  |
| 1k1dA | 0.83 | 0.41 | 0.57 | 0.87 | 0.385 | 0.47 | 32 | 318 | 47 | 24 | 102/222 | 54/144 | 48/78 |  |  |
| 1lvoA | 0.78 | 0.39 | 0.65 | 0.81 | 0.382 | 0.49 | 28 | 181 | 43 | 15 | 91/139 | 91/139 |  |  |  |
| 1k83L | 0.61 | 0.55 | 1 | 0.26 | 0.38 | 0.71 | 21 | 6 | 17 | 0 | 112/121 | 112/121 |  |  |  |
| 1mpyA | 0.75 | 0.54 | 0.57 | 0.81 | 0.38 | 0.56 | 44 | 162 | 37 | 33 | 180/329 | 180/329 |  |  |  |
| 1dj0A | 0.77 | 0.38 | 0.69 | 0.78 | 0.379 | 0.49 | 27 | 159 | 45 | 12 | 97/156 | 97/156 |  |  |  |
| 1jv2A | 0.77 | 0.28 | 0.81 | 0.77 | 0.378 | 0.41 | 68 | 584 | 179 | 16 | 223/310 | 167/218 | 39/66 | 17/26 |  |
| 1ynjD | 0.73 | 0.53 | 0.63 | 0.77 | 0.377 | 0.57 | 206 | 609 | 184 | 123 | 833/1423 | 821/1409 | 12/14 |  |  |
| 1bdmA | 0.8 | 0.36 | 0.65 | 0.82 | 0.374 | 0.46 | 24 | 200 | 43 | 13 | 114/171 | 114/171 |  |  |  |
| 1e0fI | 0.67 | 0.58 | 0.78 | 0.59 | 0.374 | 0.67 | 18 | 19 | 13 | 5 | 72/95 | 72/95 |  |  |  |
| 1gl2D | 0.81 | 0.83 | 0.95 | 0.33 | 0.374 | 0.88 | 38 | 4 | 8 | 2 | 171/195 | 171/195 |  |  |  |
| 3c98A | 0.85 | 0.43 | 0.48 | 0.91 | 0.373 | 0.46 | 31 | 401 | 41 | 33 | 113/272 | 33/57 | 80/215 |  |  |
| 1kf6B | 0.67 | 0.77 | 0.56 | 0.8 | 0.37 | 0.65 | 71 | 86 | 21 | 56 | 260/499 | 260/499 |  |  |  |
| 1lr5A | 0.67 | 0.4 | 0.81 | 0.63 | 0.37 | 0.54 | 30 | 75 | 45 | 7 | 89/134 | 89/134 |  |  |  |
| 1qq5A | 0.71 | 0.44 | 0.71 | 0.71 | 0.37 | 0.54 | 37 | 119 | 48 | 15 | 139/241 | 139/241 |  |  |  |
| 1l2wI | 0.83 | 0.82 | 1 | 0.17 | 0.369 | 0.9 | 45 | 2 | 10 | 0 | 210/215 | 210/215 |  |  |  |
| 1d4xG | 0.71 | 0.44 | 0.71 | 0.7 | 0.368 | 0.55 | 20 | 59 | 25 | 8 | 71/103 | 71/103 |  |  |  |
| 1e3vA | 0.7 | 0.44 | 0.73 | 0.69 | 0.368 | 0.55 | 22 | 61 | 28 | 8 | 76/118 | 76/118 |  |  |  |
| 1gt7A | 0.7 | 0.59 | 0.63 | 0.74 | 0.368 | 0.61 | 56 | 113 | 39 | 33 | 222/394 | 222/394 |  |  |  |
| 1hulA | 0.77 | 0.81 | 0.9 | 0.43 | 0.367 | 0.85 | 70 | 12 | 16 | 8 | 305/378 | 305/378 |  |  |  |
| 1cg5A | 0.67 | 0.49 | 0.78 | 0.61 | 0.366 | 0.6 | 35 | 57 | 36 | 10 | 130/189 | 130/189 |  |  |  |
| 1prtD | 0.69 | 0.71 | 0.72 | 0.65 | 0.366 | 0.71 | 41 | 31 | 17 | 16 | 159/238 | 159/238 |  |  |  |
| 1bd3A | 0.69 | 0.61 | 0.64 | 0.73 | 0.365 | 0.62 | 49 | 84 | 31 | 28 | 171/331 | 171/331 |  |  |  |
| 1jr8A | 0.59 | 0.41 | 0.93 | 0.44 | 0.365 | 0.57 | 28 | 32 | 40 | 2 | 114/133 | 114/133 |  |  |  |
| 1g4yB | 0.78 | 0.83 | 0.88 | 0.45 | 0.361 | 0.86 | 53 | 9 | 11 | 7 | 237/309 | 237/309 |  |  |  |
| 1ituA | 0.81 | 0.42 | 0.53 | 0.86 | 0.359 | 0.47 | 27 | 233 | 37 | 24 | 87/213 | 87/213 |  |  |  |
| 1bvnT | 0.67 | 0.52 | 0.74 | 0.64 | 0.357 | 0.61 | 17 | 28 | 16 | 6 | 79/116 | 79/116 |  |  |  |
| 1tbrR | 0.65 | 0.55 | 0.81 | 0.55 | 0.355 | 0.65 | 33 | 33 | 27 | 8 | 121/173 | 121/173 |  |  |  |
| 1tiiD | 0.69 | 0.72 | 0.78 | 0.56 | 0.355 | 0.75 | 43 | 22 | 17 | 12 | 171/243 | 171/243 |  |  |  |
| 1o6sB | 0.7 | 0.54 | 0.64 | 0.73 | 0.354 | 0.58 | 21 | 49 | 18 | 12 | 70/128 | 70/128 |  |  |  |
| 1hiaI | 0.62 | 0.45 | 0.87 | 0.5 | 0.352 | 0.59 | 13 | 16 | 16 | 2 | 72/77 | 72/77 |  |  |  |
| 1jt6A | 0.69 | 0.38 | 0.76 | 0.67 | 0.351 | 0.51 | 28 | 93 | 46 | 9 | 98/161 | 98/161 |  |  |  |
| 1mz9A | 0.86 | 0.86 | 1 | 0.14 | 0.351 | 0.93 | 37 | 1 | 6 | 0 | 184/192 | 184/192 |  |  |  |
| 1qo0A | 0.9 | 0.44 | 0.38 | 0.95 | 0.351 | 0.41 | 12 | 288 | 15 | 20 | 49/136 | 49/136 |  |  |  |
| 1a4xA | 0.81 | 0.39 | 0.55 | 0.86 | 0.35 | 0.45 | 12 | 113 | 19 | 10 | 38/85 | 38/85 |  |  |  |
| 1swuA | 0.67 | 0.71 | 0.59 | 0.76 | 0.35 | 0.64 | 34 | 44 | 14 | 24 | 118/225 | 118/225 |  |  |  |
| 1jy2P | 0.81 | 0.84 | 0.94 | 0.33 | 0.348 | 0.89 | 32 | 3 | 6 | 2 | 156/172 | 156/172 |  |  |  |
| 1fltX | 0.67 | 0.37 | 0.79 | 0.63 | 0.347 | 0.5 | 15 | 45 | 26 | 4 | 61/81 | 61/81 |  |  |  |
| 1qgwA | 0.81 | 0.89 | 0.89 | 0.46 | 0.347 | 0.89 | 54 | 6 | 7 | 7 | 254/307 | 254/307 |  |  |  |
| 1g64A | 0.65 | 0.51 | 0.79 | 0.57 | 0.346 | 0.62 | 44 | 56 | 42 | 12 | 170/237 | 170/237 |  |  |  |
| 1i4dA | 0.61 | 0.48 | 0.88 | 0.47 | 0.346 | 0.62 | 57 | 54 | 62 | 8 | 236/282 | 236/282 |  |  |  |
| 1hssA | 0.68 | 0.48 | 0.7 | 0.67 | 0.343 | 0.57 | 21 | 47 | 23 | 9 | 65/110 | 65/110 |  |  |  |
| 1qfhA | 0.67 | 0.46 | 0.73 | 0.65 | 0.343 | 0.56 | 41 | 89 | 49 | 15 | 146/243 | 146/243 |  |  |  |
| 1jy2O | 0.78 | 0.85 | 0.88 | 0.46 | 0.341 | 0.86 | 35 | 5 | 6 | 5 | 170/198 | 170/198 |  |  |  |
| 1pvuA | 0.64 | 0.4 | 0.81 | 0.58 | 0.341 | 0.54 | 30 | 62 | 45 | 7 | 124/171 | 124/171 |  |  |  |
| 1l0oC | 0.67 | 0.71 | 0.61 | 0.73 | 0.34 | 0.65 | 17 | 19 | 7 | 11 | 61/107 | 61/107 |  |  |  |
| 1o6sA | 0.87 | 0.49 | 0.35 | 0.95 | 0.338 | 0.4 | 18 | 329 | 19 | 34 | 68/183 | 68/183 |  |  |  |
| 1ixsA | 0.61 | 0.42 | 0.85 | 0.52 | 0.336 | 0.56 | 11 | 16 | 15 | 2 | 52/66 | 52/66 |  |  |  |
| 1nf3C | 0.54 | 0.31 | 0.96 | 0.43 | 0.335 | 0.47 | 23 | 39 | 51 | 1 | 106/116 | 106/116 |  |  |  |
| 3sdhA | 0.74 | 0.33 | 0.68 | 0.75 | 0.333 | 0.45 | 15 | 88 | 30 | 7 | 53/93 | 53/93 |  |  |  |
| 1k20A | 0.82 | 0.29 | 0.62 | 0.84 | 0.332 | 0.39 | 16 | 213 | 40 | 10 | 70/121 | 70/121 |  |  |  |
| 1hyhA | 0.71 | 0.56 | 0.52 | 0.8 | 0.331 | 0.54 | 43 | 137 | 34 | 39 | 149/356 | 149/356 |  |  |  |
| 1d4fA | 0.71 | 0.51 | 0.58 | 0.76 | 0.33 | 0.54 | 67 | 205 | 65 | 48 | 261/507 | 261/507 |  |  |  |
| 1kacB | 0.76 | 0.43 | 0.54 | 0.82 | 0.33 | 0.48 | 13 | 75 | 17 | 11 | 40/90 | 40/90 |  |  |  |
| 1hq3D | 0.75 | 0.75 | 0.97 | 0.25 | 0.329 | 0.85 | 55 | 6 | 18 | 2 | 248/276 | 248/276 |  |  |  |
| 1juhA | 0.74 | 0.38 | 0.61 | 0.77 | 0.326 | 0.47 | 35 | 196 | 58 | 22 | 116/244 | 107/234 | 9/10 |  |  |
| 1o94D | 0.7 | 0.51 | 0.6 | 0.74 | 0.325 | 0.55 | 30 | 82 | 29 | 20 | 120/219 | 120/219 |  |  |  |
| 1cmxA | 0.71 | 0.46 | 0.6 | 0.75 | 0.323 | 0.52 | 31 | 113 | 37 | 21 | 113/211 | 113/211 |  |  |  |
| 1jpyA | 0.7 | 0.7 | 0.91 | 0.36 | 0.323 | 0.79 | 68 | 16 | 29 | 7 | 289/357 | 289/357 |  |  |  |
| 1jv2B | 0.76 | 0.33 | 0.64 | 0.78 | 0.322 | 0.43 | 47 | 336 | 97 | 27 | 159/295 | 86/197 | 48/73 | 25/25 |  |
| 1mjgM | 0.88 | 0.32 | 0.46 | 0.92 | 0.322 | 0.38 | 24 | 544 | 50 | 28 | 86/223 | 86/223 |  |  |  |
| 1gl4B | 0.66 | 0.42 | 0.73 | 0.63 | 0.32 | 0.53 | 16 | 38 | 22 | 6 | 69/100 | 69/100 |  |  |  |
| 1avgI | 0.79 | 0.39 | 0.5 | 0.85 | 0.318 | 0.44 | 11 | 96 | 17 | 11 | 31/87 | 31/87 |  |  |  |
| 1gh6A | 0.63 | 0.25 | 0.87 | 0.59 | 0.315 | 0.39 | 13 | 57 | 39 | 2 | 67/80 | 67/80 |  |  |  |
| 1ihoA | 0.79 | 0.3 | 0.6 | 0.82 | 0.315 | 0.4 | 18 | 186 | 42 | 12 | 61/130 | 61/130 |  |  |  |
| 1jy2N | 0.79 | 0.82 | 0.94 | 0.3 | 0.315 | 0.87 | 31 | 3 | 7 | 2 | 161/182 | 161/182 |  |  |  |
| 1ci6A | 0.58 | 0.53 | 1 | 0.19 | 0.314 | 0.69 | 25 | 5 | 22 | 0 | 104/118 | 104/118 |  |  |  |
| 1g31A | 0.59 | 0.49 | 0.9 | 0.38 | 0.313 | 0.64 | 36 | 23 | 37 | 4 | 128/156 | 128/156 |  |  |  |
| 1kjyB | 0.83 | 0.87 | 0.93 | 0.33 | 0.313 | 0.9 | 27 | 2 | 4 | 2 | 119/150 | 119/150 |  |  |  |
| 1n13A | 0.91 | 0.93 | 0.98 | 0.25 | 0.312 | 0.95 | 40 | 1 | 3 | 1 | 206/228 | 206/228 |  |  |  |
| 1flmA | 0.72 | 0.44 | 0.57 | 0.77 | 0.311 | 0.5 | 16 | 65 | 20 | 12 | 59/113 | 59/113 |  |  |  |
| 1f9aA | 0.7 | 0.53 | 0.53 | 0.78 | 0.31 | 0.53 | 25 | 77 | 22 | 22 | 101/187 | 101/187 |  |  |  |
| 1h7eA | 0.78 | 0.35 | 0.55 | 0.82 | 0.309 | 0.42 | 18 | 158 | 34 | 15 | 80/152 | 80/152 |  |  |  |
| 1h9sA | 0.72 | 0.51 | 0.5 | 0.81 | 0.309 | 0.51 | 18 | 71 | 17 | 18 | 66/151 | 66/151 |  |  |  |
| 1mjhA | 0.76 | 0.38 | 0.54 | 0.81 | 0.309 | 0.45 | 13 | 89 | 21 | 11 | 53/108 | 53/108 |  |  |  |
| 1klfB | 0.62 | 0.28 | 0.84 | 0.58 | 0.306 | 0.42 | 36 | 126 | 93 | 7 | 124/154 | 124/154 |  |  |  |
| 1f39A | 0.64 | 0.53 | 0.74 | 0.57 | 0.305 | 0.62 | 29 | 34 | 26 | 10 | 99/146 | 99/146 |  |  |  |
| 1g5hA | 0.66 | 0.36 | 0.72 | 0.65 | 0.305 | 0.48 | 59 | 193 | 105 | 23 | 223/333 | 223/333 |  |  |  |
| 1prtA | 0.68 | 0.39 | 0.67 | 0.69 | 0.305 | 0.49 | 32 | 110 | 50 | 16 | 119/200 | 119/200 |  |  |  |
| 1m1eB | 0.64 | 0.61 | 0.87 | 0.39 | 0.303 | 0.72 | 27 | 11 | 17 | 4 | 110/136 | 69/79 | 41/57 |  |  |
| 1ll0A | 0.67 | 0.27 | 0.77 | 0.66 | 0.302 | 0.4 | 26 | 135 | 70 | 8 | 98/130 | 98/130 |  |  |  |
| 1otgA | 0.66 | 0.65 | 0.81 | 0.47 | 0.301 | 0.72 | 51 | 25 | 28 | 12 | 206/278 | 206/278 |  |  |  |
| 1fo0B | 0.64 | 0.39 | 0.74 | 0.61 | 0.3 | 0.51 | 20 | 49 | 32 | 7 | 93/140 | 93/140 |  |  |  |
| 1nrjB | 0.81 | 0.39 | 0.44 | 0.87 | 0.299 | 0.41 | 12 | 129 | 19 | 15 | 45/101 | 45/101 |  |  |  |
| 1dqnA | 0.76 | 0.44 | 0.47 | 0.84 | 0.297 | 0.45 | 20 | 136 | 26 | 23 | 67/183 | 67/183 |  |  |  |
| 1do8A | 0.81 | 0.26 | 0.57 | 0.84 | 0.296 | 0.36 | 27 | 389 | 76 | 20 | 110/225 | 110/225 |  |  |  |
| 1jx2B | 0.76 | 0.17 | 0.81 | 0.75 | 0.295 | 0.28 | 13 | 195 | 64 | 3 | 56/67 | 56/67 |  |  |  |
| 1nmmB | 0.66 | 0.16 | 0.94 | 0.65 | 0.295 | 0.27 | 15 | 147 | 81 | 1 | 51/68 | 51/68 |  |  |  |
| 1k3sA | 0.59 | 0.39 | 0.83 | 0.49 | 0.293 | 0.53 | 24 | 37 | 38 | 5 | 88/117 | 88/117 |  |  |  |
| 1axiB | 0.68 | 0.46 | 0.61 | 0.7 | 0.291 | 0.52 | 31 | 88 | 37 | 20 | 114/226 | 114/226 |  |  |  |
| 1gl2C | 0.78 | 0.81 | 0.94 | 0.29 | 0.291 | 0.87 | 43 | 4 | 10 | 3 | 184/225 | 184/225 |  |  |  |
| 1jv1A | 0.79 | 0.34 | 0.5 | 0.84 | 0.291 | 0.4 | 32 | 333 | 63 | 32 | 100/262 | 100/262 |  |  |  |
| 1mbxC | 0.5 | 0.32 | 0.95 | 0.36 | 0.29 | 0.47 | 18 | 22 | 39 | 1 | 64/77 | 64/77 |  |  |  |
| 1huxA | 0.65 | 0.15 | 0.93 | 0.63 | 0.288 | 0.26 | 14 | 132 | 77 | 1 | 42/57 | 42/57 |  |  |  |
| 1abrB | 0.68 | 0.3 | 0.71 | 0.67 | 0.286 | 0.42 | 29 | 140 | 69 | 12 | 129/183 | 129/183 |  |  |  |
| 1g73C | 0.64 | 0.5 | 0.69 | 0.61 | 0.286 | 0.58 | 20 | 31 | 20 | 9 | 51/113 | 51/113 |  |  |  |
| 1f45A | 0.71 | 0.2 | 0.78 | 0.7 | 0.285 | 0.32 | 18 | 168 | 73 | 5 | 69/104 | 69/104 |  |  |  |
| 1hq3C | 0.7 | 0.7 | 0.95 | 0.24 | 0.285 | 0.8 | 57 | 8 | 25 | 3 | 258/298 | 258/298 |  |  |  |
| 1m3eA | 0.76 | 0.3 | 0.58 | 0.78 | 0.285 | 0.4 | 35 | 296 | 82 | 25 | 116/241 | 116/241 |  |  |  |
| 1k2fA | 0.67 | 0.27 | 0.73 | 0.66 | 0.283 | 0.39 | 19 | 102 | 52 | 7 | 75/117 | 75/117 |  |  |  |
| 1lhpA | 0.74 | 0.35 | 0.55 | 0.78 | 0.283 | 0.43 | 27 | 184 | 51 | 22 | 99/209 | 99/209 |  |  |  |
| 2vglA | 0.7 | 0.42 | 0.56 | 0.75 | 0.282 | 0.48 | 78 | 324 | 108 | 62 | 292/564 | 292/564 |  |  |  |
| 1evxA | 0.6 | 0.48 | 0.81 | 0.47 | 0.281 | 0.6 | 47 | 46 | 52 | 11 | 205/269 | 205/269 |  |  |  |
| 1he1A | 0.69 | 0.4 | 0.6 | 0.72 | 0.281 | 0.48 | 18 | 68 | 27 | 12 | 46/105 | 46/105 |  |  |  |
| 1k28D | 0.64 | 0.35 | 0.73 | 0.61 | 0.28 | 0.47 | 56 | 163 | 105 | 21 | 188/321 | 188/321 |  |  |  |
| 1h4lD | 0.62 | 0.41 | 0.74 | 0.57 | 0.279 | 0.52 | 28 | 55 | 41 | 10 | 103/155 | 103/155 |  |  |  |
| 1b34B | 0.65 | 0.67 | 0.73 | 0.55 | 0.277 | 0.7 | 29 | 17 | 14 | 11 | 104/163 | 104/163 |  |  |  |
| 1dqsA | 0.81 | 0.33 | 0.46 | 0.86 | 0.277 | 0.39 | 20 | 246 | 40 | 24 | 71/178 | 71/178 |  |  |  |
| 2vglS | 0.61 | 0.79 | 0.53 | 0.76 | 0.277 | 0.63 | 45 | 37 | 12 | 40 | 164/354 | 164/354 |  |  |  |
| 1ek9A | 0.66 | 0.43 | 0.64 | 0.66 | 0.276 | 0.52 | 76 | 195 | 101 | 42 | 271/517 | 271/517 |  |  |  |
| 1bvyF | 0.71 | 0.32 | 0.61 | 0.74 | 0.275 | 0.42 | 14 | 83 | 30 | 9 | 48/94 | 48/94 |  |  |  |
| 1devB | 0.75 | 0.77 | 0.93 | 0.27 | 0.275 | 0.84 | 27 | 3 | 8 | 2 | 116/126 | 116/126 |  |  |  |
| 1e2tA | 0.71 | 0.3 | 0.63 | 0.72 | 0.275 | 0.41 | 26 | 154 | 60 | 15 | 110/174 | 110/174 |  |  |  |
| 1fqjC | 0.54 | 0.5 | 1 | 0.15 | 0.274 | 0.67 | 17 | 3 | 17 | 0 | 57/65 | 57/65 |  |  |  |
| 1tocR | 0.62 | 0.51 | 0.71 | 0.57 | 0.274 | 0.59 | 32 | 41 | 31 | 13 | 104/179 | 104/179 |  |  |  |
| 1jmaA | 0.63 | 0.24 | 0.79 | 0.6 | 0.273 | 0.37 | 27 | 128 | 85 | 7 | 83/130 | 83/130 |  |  |  |
| 1jxhA | 0.65 | 0.38 | 0.66 | 0.65 | 0.272 | 0.48 | 35 | 107 | 57 | 18 | 140/231 | 140/231 |  |  |  |
| 1dowB | 0.74 | 0.75 | 0.96 | 0.22 | 0.271 | 0.84 | 21 | 2 | 7 | 1 | 99/114 | 99/114 |  |  |  |
| 1sgpE | 0.68 | 0.28 | 0.68 | 0.68 | 0.271 | 0.4 | 17 | 92 | 43 | 8 | 45/77 | 45/77 |  |  |  |
| 1h0hA | 0.9 | 0.29 | 0.37 | 0.93 | 0.27 | 0.32 | 21 | 735 | 52 | 36 | 77/221 | 77/221 |  |  |  |
| 1aa7A | 0.75 | 0.35 | 0.5 | 0.81 | 0.269 | 0.41 | 12 | 91 | 22 | 12 | 51/108 | 51/108 |  |  |  |
| 1cseE | 0.73 | 0.27 | 0.62 | 0.75 | 0.267 | 0.37 | 18 | 146 | 50 | 11 | 49/91 | 49/91 |  |  |  |
| 1kxpD | 0.72 | 0.24 | 0.66 | 0.73 | 0.266 | 0.35 | 31 | 272 | 100 | 16 | 95/183 | 95/183 |  |  |  |
| 1h6kA | 0.8 | 0.16 | 0.7 | 0.81 | 0.265 | 0.26 | 23 | 502 | 121 | 10 | 77/131 | 77/131 |  |  |  |
| 1nbwB | 0.63 | 0.38 | 0.69 | 0.61 | 0.264 | 0.49 | 18 | 45 | 29 | 8 | 68/127 | 68/127 |  |  |  |
| 1prtF | 0.64 | 0.63 | 0.75 | 0.51 | 0.264 | 0.69 | 38 | 23 | 22 | 13 | 155/220 | 155/220 |  |  |  |
| 1bi7B | 0.63 | 0.5 | 0.66 | 0.61 | 0.258 | 0.57 | 27 | 42 | 27 | 14 | 87/152 | 87/152 |  |  |  |
| 1kveB | 0.78 | 0.82 | 0.92 | 0.29 | 0.258 | 0.86 | 54 | 5 | 12 | 5 | 189/257 | 189/257 |  |  |  |
| 1ixsB | 0.74 | 0.16 | 0.74 | 0.74 | 0.256 | 0.26 | 14 | 208 | 73 | 5 | 49/66 | 49/66 |  |  |  |
| 1jkgB | 0.59 | 0.41 | 0.76 | 0.52 | 0.256 | 0.53 | 40 | 63 | 58 | 13 | 141/203 | 141/203 |  |  |  |
| 1fs1B | 0.64 | 0.46 | 0.61 | 0.65 | 0.25 | 0.52 | 22 | 49 | 26 | 14 | 76/146 | 67/85 | 9/61 |  |  |
| 1mt5A | 0.73 | 0.2 | 0.67 | 0.73 | 0.25 | 0.31 | 26 | 290 | 105 | 13 | 97/172 | 97/172 |  |  |  |
| 1dekA | 0.76 | 0.22 | 0.59 | 0.78 | 0.249 | 0.33 | 13 | 155 | 45 | 9 | 46/84 | 46/84 |  |  |  |
| 1e5dA | 0.75 | 0.28 | 0.53 | 0.79 | 0.249 | 0.37 | 26 | 248 | 67 | 23 | 101/196 | 101/196 |  |  |  |
| 2ccyA | 0.6 | 0.24 | 0.78 | 0.57 | 0.248 | 0.37 | 14 | 58 | 44 | 4 | 47/84 | 47/84 |  |  |  |
| 1k90A | 0.72 | 0.43 | 0.43 | 0.81 | 0.247 | 0.43 | 48 | 276 | 63 | 63 | 176/426 | 166/332 | 10/94 |  |  |
| 1ccwB | 0.81 | 0.21 | 0.53 | 0.84 | 0.245 | 0.3 | 16 | 314 | 62 | 14 | 47/124 | 47/124 |  |  |  |
| 1xdtR | 0.65 | 0.64 | 0.96 | 0.2 | 0.244 | 0.76 | 21 | 3 | 12 | 1 | 74/95 | 74/95 |  |  |  |
| 1agqA | 0.57 | 0.47 | 0.83 | 0.4 | 0.241 | 0.6 | 29 | 22 | 33 | 6 | 103/140 | 103/140 |  |  |  |
| 1bo1A | 0.73 | 0.22 | 0.61 | 0.75 | 0.24 | 0.32 | 19 | 203 | 69 | 12 | 70/130 | 70/130 |  |  |  |
| 1gh6B | 0.75 | 0.19 | 0.63 | 0.76 | 0.238 | 0.29 | 15 | 209 | 65 | 9 | 55/91 | 55/91 |  |  |  |
| 1mg2D | 0.7 | 0.19 | 0.69 | 0.7 | 0.238 | 0.3 | 9 | 90 | 39 | 4 | 35/53 | 35/53 |  |  |  |
| 1b6sA | 0.84 | 0.3 | 0.36 | 0.89 | 0.234 | 0.33 | 13 | 262 | 31 | 23 | 30/132 | 30/132 |  |  |  |
| 1f02T | 0.63 | 0.74 | 0.63 | 0.61 | 0.234 | 0.68 | 26 | 14 | 9 | 15 | 74/163 | 74/163 |  |  |  |
| 1gl2A | 0.78 | 0.84 | 0.9 | 0.3 | 0.234 | 0.87 | 37 | 3 | 7 | 4 | 177/212 | 177/212 |  |  |  |
| 1gyxA | 0.65 | 0.65 | 0.91 | 0.27 | 0.234 | 0.76 | 40 | 8 | 22 | 4 | 153/192 | 153/192 |  |  |  |
| 1fxkA | 0.58 | 0.35 | 0.73 | 0.53 | 0.229 | 0.47 | 19 | 41 | 36 | 7 | 77/119 | 65/107 | 12/12 |  |  |
| 1kilE | 0.6 | 0.52 | 0.71 | 0.52 | 0.228 | 0.6 | 12 | 12 | 11 | 5 | 51/78 | 51/78 |  |  |  |
| 1a38A | 0.69 | 0.34 | 0.54 | 0.73 | 0.227 | 0.42 | 23 | 117 | 44 | 20 | 66/145 | 52/97 | 14/48 |  |  |
| 1jzdC | 0.65 | 0.4 | 0.57 | 0.68 | 0.227 | 0.47 | 17 | 56 | 26 | 13 | 57/103 | 42/68 | 15/35 |  |  |
| 1cjxA | 0.79 | 0.18 | 0.57 | 0.81 | 0.226 | 0.27 | 13 | 253 | 61 | 10 | 40/91 | 40/91 |  |  |  |
| 2trcP | 0.61 | 0.37 | 0.67 | 0.59 | 0.226 | 0.48 | 32 | 77 | 54 | 16 | 126/219 | 126/219 |  |  |  |
| 1nbwA | 0.71 | 0.28 | 0.54 | 0.74 | 0.223 | 0.37 | 48 | 353 | 123 | 41 | 169/341 | 102/201 | 67/140 |  |  |
| 1g73A | 0.5 | 0.36 | 0.87 | 0.36 | 0.221 | 0.5 | 39 | 39 | 71 | 6 | 132/169 | 108/144 | 24/25 |  |  |
| 1preA | 0.77 | 0.29 | 0.44 | 0.82 | 0.221 | 0.35 | 27 | 301 | 65 | 35 | 110/257 | 2/11 | 21/64 | 65/120 | 22/62 |
| 1g8eA | 0.63 | 0.62 | 0.93 | 0.23 | 0.219 | 0.75 | 51 | 9 | 31 | 4 | 208/252 | 208/252 |  |  |  |
| 1eerA | 0.61 | 0.33 | 0.65 | 0.6 | 0.214 | 0.44 | 24 | 73 | 48 | 13 | 83/168 | 83/168 |  |  |  |
| 1im3D | 0.58 | 0.28 | 0.72 | 0.55 | 0.212 | 0.4 | 13 | 41 | 34 | 5 | 48/68 | 48/68 |  |  |  |
| 1t83C | 0.62 | 0.19 | 0.72 | 0.61 | 0.212 | 0.3 | 13 | 88 | 56 | 5 | 65/93 | 65/93 |  |  |  |
| 1afrA | 0.74 | 0.4 | 0.35 | 0.85 | 0.21 | 0.37 | 25 | 213 | 38 | 46 | 64/289 | 64/289 |  |  |  |
| 1ci6B | 0.62 | 0.61 | 0.96 | 0.16 | 0.207 | 0.75 | 25 | 3 | 16 | 1 | 105/125 | 105/125 |  |  |  |
| 1kmiZ | 0.58 | 0.56 | 0.94 | 0.2 | 0.2 | 0.7 | 86 | 17 | 68 | 6 | 325/385 | 307/358 | 18/27 |  |  |
| 2rspA | 0.52 | 0.33 | 0.79 | 0.43 | 0.2 | 0.47 | 23 | 34 | 46 | 6 | 100/143 | 100/143 |  |  |  |
| 1dowA | 0.59 | 0.26 | 0.69 | 0.56 | 0.199 | 0.38 | 25 | 92 | 71 | 11 | 79/129 | 79/129 |  |  |  |
| 1eerB | 0.7 | 0.22 | 0.56 | 0.72 | 0.194 | 0.31 | 14 | 128 | 51 | 11 | 42/104 | 42/104 |  |  |  |
| 1jnrB | 0.66 | 0.71 | 0.82 | 0.36 | 0.194 | 0.76 | 80 | 18 | 32 | 18 | 373/494 | 373/494 |  |  |  |
| 1gvnB | 0.79 | 0.29 | 0.34 | 0.87 | 0.193 | 0.31 | 12 | 192 | 30 | 23 | 37/151 | 37/151 |  |  |  |
| 1cruA | 0.86 | 0.23 | 0.3 | 0.91 | 0.192 | 0.26 | 10 | 345 | 33 | 23 | 26/124 | 26/124 |  |  |  |
| 1azsA | 0.56 | 0.37 | 0.71 | 0.49 | 0.19 | 0.49 | 35 | 57 | 59 | 14 | 148/225 | 136/204 | 12/21 |  |  |
| 1m7gA | 0.73 | 0.2 | 0.53 | 0.75 | 0.19 | 0.29 | 10 | 125 | 41 | 9 | 36/82 | 36/82 |  |  |  |
| 1nbfA | 0.76 | 0.29 | 0.38 | 0.83 | 0.189 | 0.33 | 20 | 236 | 48 | 33 | 78/219 | 78/219 |  |  |  |
| 1ldjB | 0.53 | 0.46 | 0.86 | 0.3 | 0.182 | 0.6 | 30 | 15 | 35 | 5 | 203/224 | 203/224 |  |  |  |
| 1ev2E | 0.58 | 0.44 | 0.66 | 0.53 | 0.178 | 0.53 | 38 | 54 | 48 | 20 | 121/229 | 121/229 |  |  |  |
| 1gl0I | 0.61 | 0.64 | 0.78 | 0.39 | 0.177 | 0.7 | 14 | 5 | 8 | 4 | 65/80 | 65/80 |  |  |  |
| 1go3E | 0.54 | 0.41 | 0.73 | 0.45 | 0.17 | 0.52 | 37 | 44 | 54 | 14 | 122/215 | 122/215 |  |  |  |
| 1euvB | 0.61 | 0.46 | 0.48 | 0.69 | 0.167 | 0.47 | 12 | 31 | 14 | 13 | 58/108 | 58/108 |  |  |  |
| 1jthA | 0.81 | 0.87 | 0.92 | 0.22 | 0.167 | 0.89 | 45 | 2 | 7 | 4 | 208/249 | 208/249 |  |  |  |
| 1kshB | 0.68 | 0.26 | 0.48 | 0.73 | 0.165 | 0.34 | 11 | 82 | 31 | 12 | 40/88 | 40/88 |  |  |  |
| 1im9D | 0.59 | 0.16 | 0.68 | 0.58 | 0.163 | 0.26 | 13 | 96 | 69 | 6 | 45/75 | 45/75 |  |  |  |
| 1gx1A | 0.6 | 0.44 | 0.52 | 0.64 | 0.157 | 0.48 | 27 | 61 | 34 | 25 | 83/205 | 83/205 |  |  |  |
| 1hc9A | 0.52 | 0.31 | 0.72 | 0.45 | 0.155 | 0.43 | 13 | 24 | 29 | 5 | 42/71 | 42/71 |  |  |  |
| 1jjuC | 0.75 | 0.88 | 0.82 | 0.36 | 0.154 | 0.85 | 53 | 4 | 7 | 12 | 220/311 | 220/311 |  |  |  |
| 1gzsB | 0.54 | 0.35 | 0.67 | 0.49 | 0.15 | 0.46 | 29 | 53 | 55 | 14 | 84/161 | 84/161 |  |  |  |
| 1ktzB | 0.65 | 0.3 | 0.46 | 0.7 | 0.137 | 0.36 | 10 | 54 | 23 | 12 | 45/100 | 45/100 |  |  |  |
| 1m2oA | 0.74 | 0.11 | 0.49 | 0.76 | 0.133 | 0.18 | 19 | 482 | 152 | 20 | 78/179 | 78/179 |  |  |  |
| 1ev7A | 0.83 | 0.22 | 0.23 | 0.9 | 0.132 | 0.23 | 7 | 230 | 25 | 23 | 23/136 | 23/136 |  |  |  |
| 1ghqB | 0.53 | 0.33 | 0.67 | 0.48 | 0.131 | 0.44 | 24 | 44 | 48 | 12 | 79/143 | 52/94 | 27/49 |  |  |
| 1qbkB | 0.61 | 0.11 | 0.62 | 0.61 | 0.12 | 0.19 | 37 | 465 | 297 | 23 | 116/235 | 82/133 | 34/102 |  |  |
| 1foeA | 0.72 | 0.17 | 0.42 | 0.76 | 0.119 | 0.24 | 15 | 234 | 76 | 21 | 45/168 | 45/168 |  |  |  |
| 1d3bB | 0.55 | 0.5 | 0.63 | 0.49 | 0.117 | 0.56 | 22 | 21 | 22 | 13 | 70/141 | 70/141 |  |  |  |
| 1h2iA | 0.66 | 0.74 | 0.79 | 0.32 | 0.117 | 0.77 | 98 | 16 | 34 | 26 | 383/595 | 383/595 |  |  |  |
| 1gl1I | 0.58 | 0.62 | 0.68 | 0.43 | 0.116 | 0.65 | 13 | 6 | 8 | 6 | 60/80 | 60/80 |  |  |  |
| 1miuA | 0.6 | 0.24 | 0.53 | 0.61 | 0.115 | 0.33 | 62 | 317 | 201 | 54 | 218/454 | 206/395 | 12/59 |  |  |
| 1gc1C | 0.8 | 0.25 | 0.21 | 0.9 | 0.113 | 0.23 | 5 | 129 | 15 | 19 | 21/98 | 21/98 |  |  |  |
| 1lqsR | 0.6 | 0.17 | 0.56 | 0.61 | 0.111 | 0.26 | 14 | 104 | 68 | 11 | 48/95 | 48/95 |  |  |  |
| 1ay7A | 0.6 | 0.25 | 0.5 | 0.63 | 0.106 | 0.33 | 9 | 46 | 27 | 9 | 38/69 | 38/69 |  |  |  |
| 1cd9B | 0.6 | 0.19 | 0.54 | 0.61 | 0.106 | 0.28 | 15 | 103 | 65 | 13 | 62/118 | 41/92 | 21/26 |  |  |
| 1fm9D | 0.66 | 0.24 | 0.41 | 0.71 | 0.103 | 0.3 | 19 | 150 | 61 | 27 | 60/183 | 60/183 |  |  |  |
| 1e96B | 0.65 | 0.14 | 0.47 | 0.67 | 0.093 | 0.22 | 8 | 99 | 48 | 9 | 21/65 | 21/65 |  |  |  |
| 1dm5A | 0.74 | 0.32 | 0.17 | 0.9 | 0.089 | 0.22 | 11 | 205 | 23 | 54 | 34/224 | 34/224 |  |  |  |
| 1k8rB | 0.46 | 0.19 | 0.69 | 0.42 | 0.083 | 0.3 | 9 | 28 | 39 | 4 | 20/56 | 20/56 |  |  |  |
| 1aroP | 0.71 | 0.07 | 0.45 | 0.72 | 0.074 | 0.11 | 14 | 506 | 200 | 17 | 49/130 | 49/130 |  |  |  |
| 1qa9B | 0.52 | 0.22 | 0.59 | 0.51 | 0.074 | 0.32 | 10 | 37 | 36 | 7 | 24/70 | 24/70 |  |  |  |
| 1fyhB | 0.55 | 0.17 | 0.56 | 0.54 | 0.07 | 0.26 | 15 | 87 | 73 | 12 | 41/99 | 41/99 |  |  |  |
| 1zbdB | 0.48 | 0.36 | 0.68 | 0.39 | 0.059 | 0.47 | 27 | 30 | 48 | 13 | 99/188 | 99/188 |  |  |  |
| 1go4E | 0.62 | 0.63 | 0.96 | 0.06 | 0.055 | 0.76 | 52 | 2 | 31 | 2 | 239/284 | 239/284 |  |  |  |
| 1h6kX | 0.53 | 0.46 | 0.52 | 0.54 | 0.055 | 0.48 | 15 | 21 | 18 | 14 | 60/134 | 60/134 |  |  |  |
| 1lm7A | 0.76 | 0.14 | 0.24 | 0.82 | 0.048 | 0.18 | 6 | 164 | 36 | 19 | 22/90 | 22/90 |  |  |  |
| 1dx5I | 0.35 | 0.18 | 0.8 | 0.25 | 0.046 | 0.3 | 16 | 24 | 71 | 4 | 49/82 | 49/82 |  |  |  |
| 1mbxA | 0.57 | 0.21 | 0.46 | 0.6 | 0.046 | 0.29 | 11 | 63 | 42 | 13 | 38/102 | 38/102 |  |  |  |
| 1d4vA | 0.47 | 0.3 | 0.62 | 0.41 | 0.023 | 0.41 | 21 | 33 | 48 | 13 | 71/138 | 71/138 |  |  |  |
| 1jmaB | 0.49 | 0.46 | 0.71 | 0.31 | 0.022 | 0.56 | 32 | 17 | 38 | 13 | 91/147 | 49/99 | 42/48 |  |  |
| 1hx1B | 0.6 | 0.24 | 0.33 | 0.68 | 0.015 | 0.28 | 8 | 54 | 25 | 16 | 29/95 | 29/95 |  |  |  |
| 1clvI | 0.55 | 0.65 | 0.65 | 0.36 | 0.014 | 0.65 | 13 | 4 | 7 | 7 | 66/98 | 66/98 |  |  |  |
| 1fjrA | 0.59 | 0.17 | 0.38 | 0.63 | 0.01 | 0.23 | 11 | 95 | 55 | 18 | 32/91 | 32/91 |  |  |  |
| 1hqrD | 0.64 | 0.13 | 0.33 | 0.68 | 0.008 | 0.18 | 7 | 103 | 49 | 14 | 24/85 | 24/85 |  |  |  |
| 1jb0K | 0 | 0 | 0 | 0 | 0 | 0 | 0 | 7 | 0 | 5 | 0/0 |  |  |  |  |
| 1gpwA | 0.76 | 0.13 | 0.12 | 0.86 | -0.016 | 0.12 | 4 | 176 | 28 | 29 | 11/131 | 11/131 |  |  |  |
| 1g3jA | 0.61 | 0.18 | 0.3 | 0.68 | -0.017 | 0.22 | 24 | 236 | 109 | 57 | 64/279 | 64/279 |  |  |  |
| 1icwA | 0.46 | 0.36 | 0.6 | 0.37 | -0.028 | 0.45 | 15 | 16 | 27 | 10 | 52/114 | 52/114 |  |  |  |
| 1ospO | 0.83 | 0.04 | 0.05 | 0.9 | -0.05 | 0.04 | 1 | 205 | 22 | 21 | 2/82 | 2/82 |  |  |  |
| 1nmuA | 0.88 | 0 | 0 | 0.92 | -0.056 | 0 | 0 | 306 | 28 | 12 | 0/43 | 0/43 |  |  |  |
| 1kveA | 0.74 | 0.75 | 0.98 | 0 | -0.074 | 0.85 | 45 | 0 | 15 | 1 | 216/229 | 216/229 |  |  |  |
| 1tiiC | 0.81 | 0.83 | 0.97 | 0 | -0.076 | 0.89 | 29 | 0 | 6 | 1 | 160/168 | 160/168 |  |  |  |
| 1l6rA | 0.7 | 0.05 | 0.09 | 0.79 | -0.104 | 0.07 | 2 | 132 | 36 | 21 | 10/78 | 10/78 |  |  |  |
| 1ghqA | 0.77 | 0 | 0 | 0.82 | -0.119 | 0 | 0 | 200 | 44 | 17 | 0/79 | 0/79 |  |  |  |
| 1g3jB | 0.76 | 0.89 | 0.83 | 0 | -0.134 | 0.86 | 25 | 0 | 3 | 5 | 125/177 | 125/177 |  |  |  |
| 1fp3A | 0.72 | 0 | 0 | 0.78 | -0.147 | 0 | 0 | 248 | 70 | 27 | 0/126 | 0/126 |  |  |  |
| 1kg0C | 0.37 | 0.08 | 0.38 | 0.37 | -0.175 | 0.14 | 6 | 39 | 66 | 10 | 19/61 | 19/61 |  |  |  |
| 1ebdC | 0.3 | 0.26 | 0.73 | 0.12 | -0.195 | 0.38 | 8 | 3 | 23 | 3 | 31/54 | 31/54 |  |  |  |
| 1fskA | 0.58 | 0.02 | 0.05 | 0.66 | -0.214 | 0.03 | 1 | 88 | 46 | 19 | 3/82 | 3/82 |  |  |  |
| 1qa9A | 0.63 | 0 | 0 | 0.77 | -0.23 | 0 | 0 | 62 | 19 | 18 | 0/68 | 0/68 |  |  |  |
| 1b0nB | 0.9 | 0.9 | 1 | 0 | NaN | 0.95 | 28 | 0 | 3 | 0 | 134/150 | 134/150 |  |  |  |
| 1be3I | 0.97 | 0.97 | 1 | 0 | NaN | 0.98 | 31 | 0 | 1 | 0 | 155/156 | 155/156 |  |  |  |
| 1g2yA | 0.76 | 0.76 | 1 | 0 | NaN | 0.86 | 22 | 0 | 7 | 0 | 95/98 | 95/98 |  |  |  |
| 1gl2B | 0.81 | 0.81 | 1 | 0 | NaN | 0.9 | 48 | 0 | 11 | 0 | 217/239 | 217/239 |  |  |  |
| 1jb0M | 0.7 | 0.7 | 1 | 0 | NaN | 0.82 | 21 | 0 | 9 | 0 | 90/94 | 90/94 |  |  |  |
| 1svfB | 0.73 | 0.73 | 1 | 0 | NaN | 0.84 | 27 | 0 | 10 | 0 | 119/123 | 119/123 |  |  |  |
| Total | 0.75 | 0.5 | 0.71 | 0.76 | 0.424 | 0.58 | 14953 | 48528 | 15230 | 6135 |  |  |  |  |  |

**Table S3:** Five-fold cross validation of SVM_BAGGING prediction accuracy benchmarks on the S432 dataset. The dataset, the 5-fold cross validation, and the benchmark measurements have been described in the main text. Matthews correlation coefficient (MCC), F-score(Fsc), Accuracy(Acc), Precision(Pre), Sensitivity(Sen) and Specificity(Spe) are shown in Equations (6)~(11) in the main text. TP, FP, TN, and FN are true positive, false positive, true negative, and false negative respectively. The ratio of the number of predicted positive atoms against actual number of binding atoms for each protein is also listed. C1~C4 represent PPI sites in each of the test proteins; different protein has different number of PPI sites. In these columns, the number of the predicted true positive atoms is shown over the actual number of atoms involving in the PPI site. Interactive examination of the prediction results for each of the proteins in the S432 dataset can be accessed from the web server: <http://ismblab.genomics.sinica.edu.tw/>> benchmark > protein-protein.
