# Supplementary material for: Protein-Protein Interaction Site Predictions with Three-Dimensional Probability Distributions of Interacting Atoms on Protein Surfaces
Source: PLoS One. 2012 Jun 6;7(6):e37706. doi: 10.1371/journal.pone.0037706 (PMC3368894; doi:10.1371/journal.pone.0037706)
Supplement: Table S5 — Independent test of ANN_BAGGING prediction accuracy benchmarks on the S17a dataset. The dataset and the benchmark measurements have been described in the main text. Matthews correlation coefficient (MCC), F-score(Fsc), Accuracy(Acc), Precision(Pre), Sensitivity(Sen) and Specificity(Spe) are shown in Equations (6)∼(11) in the main text. TP, FP, TN, and FN are true positive, false positive, true negative, and false negative respectively. The ratio of the number of predicted positive atoms against actual number of binding atoms for each protein is also listed. C1∼C2 represent PPI sites in each of the test proteins. In these columns, the number of the predicted true positive atoms is shown over the actual number of atoms involving in the PPI site. Interactive examination of the prediction results for each of the proteins in the S17a dataset can be accessed from the web server: http://ismblab.genomics.sinica.edu.tw/> benchmark >protein-protein. (DOCX) [file pone.0037706.s008.docx]

**Table S5. Residue-based independent test benchmarks of ANN_BAGGING with S17a dataset.**

|  | Residue Level Benchmark | | | | | | | | | | Predict positive atoms / Actual binding atoms | | |
| --- | --- | --- | --- | --- | --- | --- | --- | --- | --- | --- | --- | --- | --- |
| PDBID | Acc | Pre | Sen | Spe | MCC | Fsc | TP | TN | FP | FN | All | C1 | C2 |
| 1x7nA | 0.833 | 0.63 | 0.773 | 0.853 | 0.587 | 0.694 | 34 | 116 | 20 | 10 | 138/197 | 138/197 |  |
| 1o5tA | 0.854 | 0.408 | 0.744 | 0.868 | 0.478 | 0.527 | 29 | 275 | 42 | 10 | 97/149 | 97/149 |  |
| 1z8gA | 0.657 | 0.388 | 0.76 | 0.625 | 0.329 | 0.514 | 19 | 50 | 30 | 6 | 82/124 | 82/124 |  |
| 1iniA | 0.695 | 0.36 | 0.692 | 0.696 | 0.319 | 0.474 | 27 | 110 | 48 | 12 | 109/161 | 109/161 |  |
| 1pq0A | 0.779 | 0.444 | 0.462 | 0.857 | 0.314 | 0.453 | 12 | 90 | 15 | 14 | 49/109 | 49/109 |  |
| 1onsA | 0.844 | 0.314 | 0.44 | 0.89 | 0.285 | 0.367 | 11 | 194 | 24 | 14 | 48/100 | 48/100 |  |
| 1oxzA | 0.615 | 0.155 | 0.9 | 0.592 | 0.264 | 0.265 | 9 | 71 | 49 | 1 | 35/48 | 35/48 |  |
| 1nyhA | 0.554 | 0.529 | 1 | 0.108 | 0.239 | 0.692 | 37 | 4 | 33 | 0 | 151/157 | 151/157 |  |
| 1accA | 0.906 | 0.182 | 0.417 | 0.925 | 0.232 | 0.253 | 10 | 558 | 45 | 14 | 38/94 | 38/94 |  |
| 1uw7A | 0.475 | 0.221 | 0.895 | 0.394 | 0.223 | 0.354 | 17 | 39 | 60 | 2 | 67/74 | 54/61 | 13/13 |
| 1rmrA | 0.597 | 0.455 | 0.682 | 0.55 | 0.222 | 0.545 | 15 | 22 | 18 | 7 | 47/84 | 17/30 | 30/54 |
| 1tk1A | 0.683 | 0.044 | 0.75 | 0.681 | 0.126 | 0.083 | 3 | 139 | 65 | 1 | 5/13 | 5/13 |  |
| 1shtX | 0.724 | 0.258 | 0.32 | 0.808 | 0.118 | 0.286 | 8 | 97 | 23 | 17 | 26/91 | 26/91 |  |
| 1kfnA | 0.585 | 0.743 | 0.667 | 0.357 | 0.022 | 0.703 | 26 | 5 | 9 | 13 | 93/166 | 93/166 |  |
| 1mn3A | 0.471 | 0.355 | 0.611 | 0.394 | 0.005 | 0.449 | 11 | 13 | 20 | 7 | 25/59 | 25/59 |  |
| 1kloA | 0.582 | 0.121 | 0.318 | 0.625 | -0.041 | 0.175 | 7 | 85 | 51 | 15 | 16/75 | 16/75 |  |
| 1krmA | 0.883 | 0 | 0 | 0.95 | -0.061 | 0 | 0 | 265 | 14 | 21 | 0/88 | 0/88 |  |
| Total | 0.767 | 0.327 | 0.626 | 0.79 | 0.326 | 0.43 | 275 | 2133 | 566 | 164 |  |  |  |

**Table S5:** Independent test of ANN_BAGGING prediction accuracy benchmarks on the S17a dataset. The dataset and the benchmark measurements have been described in the main text. Matthews correlation coefficient (MCC), F-score(Fsc), Accuracy(Acc), Precision(Pre), Sensitivity(Sen) and Specificity(Spe) are shown in Equations (6)~(11) in the main text. TP, FP, TN, and FN are true positive, false positive, true negative, and false negative respectively. The ratio of the number of predicted positive atoms against actual number of binding atoms for each protein is also listed. C1~C2 represent PPI sites in each of the test proteins. In these columns, the number of the predicted true positive atoms is shown over the actual number of atoms involving in the PPI site. Interactive examination of the prediction results for each of the proteins in the S17a dataset can be accessed from the web server: <http://ismblab.genomics.sinica.edu.tw/>> benchmark > protein-protein.
